# National and subnational trends of birthweight in Peru: Pooled analysis of 2,927,761 births between 2012 and 2019 from the national birth registry

**DOI:** 10.1016/j.lana.2021.100017

**Published:** 2021-09

**Authors:** Rodrigo M Carrillo-Larco, Kim N Cajachagua-Torres, Wilmer Cristobal Guzman-Vilca, Hugo G Quezada-Pinedo, Carla Tarazona-Meza, Luis Huicho

**Affiliations:** 1 CRONICAS Centre of Excellence in Chronic Diseases, Universidad Peruana Cayetano Heredia, Lima, Peru; 2 Department of Epidemiology and Biostatistics, School of Public Health, Imperial College London, London, UK; 3 The Generation R Study Group, Erasmus MC, University Medical Center Rotterdam, Rotterdam, The Netherlands; 4 The Department of Paediatrics, Erasmus MC, University Medical Center Rotterdam, Rotterdam, The Netherlands; 5 Centro de Investigación en Salud Materna e Infantil and Centro de Investigación para el Desarrollo Integral y Sostenible, Universidad Peruana Cayetano Heredia, Lima, Peru; 6 School of Medicine “Alberto Hurtado”, Universidad Peruana Cayetano Heredia, Lima, Peru; 7 Sociedad Científica de Estudiantes de Medicina Cayetano Heredia (SOCEMCH), Universidad Peruana Cayetano Heredia, Lima, Peru; 8 Center for Global Non-Communicable Disease Research and Training, Johns Hopkins University, Baltimore MD, USA; 9 Universidad Cientifica del Sur, Lima, Peru

**Keywords:** Newborns, child and maternal health, time trends, low- and middle-income countries

## Abstract

**Background:**

National and subnational characterization of birthweight profiles lacks in low- and middle-income countries, yet these are needed for monitoring the progress of national and global nutritional targets. We aimed to describe birthweight indicators at the national and subnational levels in Peru (2012-2019), and by selected correlates.

**Methods:**

We studied mean birthweight (g), low birthweight (<2,500 g) and small for gestational age (according to international growth curves) prevalences. We analysed the national birth registry and summarized the three birthweight indicators at the national, regional, and province level, also by geographic area (Coast, Highlands, and Amazon). With individual-level data from the mother, we described the birthweight indicators by age, educational level and healthcare provider. Following an ecological approach (province level), we described the birthweight indicators by human development index (HDI), altitude above sea level, proportion of the population living in poverty and proportion of rural population.

**Findings:**

Mean birthweight was always the lowest in the Highlands (2,954 g in 2019) yet the highest in the Coast (3,516 g in 2019). The same was observed for low birthweight and small for gestational age. In regions with Coast and Highlands, the birthweight indicators worsen from the Coast to the Highlands; the largest absolute difference in mean birthweight between Coast and Highlands in the same region was 367 g. All birthweight indicators were the worst in mothers with none/initial education, while they improved with higher HDI.

**Interpretation:**

This analysis suggests that interventions are needed at the province level, given the large differences observed between Coast and Highlands even in the same region.

**Funding:**

Wellcome Trust (214185/Z/18/Z)

**RESEARCH IN CONTEXT**

**Evidence before this study**

We searched PubMed on April 4^th^, 2021 with no language or date restrictions. The search strategy was: (("mean birthweight") OR ("low birthweight") OR ("small for gestational age")) AND ("national" OR "subnational") AND ("trends"). The search yielded 230 results, though we could not find publications which main or only objective was to study national and subnational time trends of mean birthweight, low birthweight prevalence and small for gestational age prevalence simultaneously. One global work studied the mean birthweight for all countries, and there were some relevant examples from a few countries like Chile and Brazil. National and subnational trends of birthweight indicators for Peru, and other low- and middle-income countries, were scarce.

**Added value of this study**

We analysed the Peruvian birth registry including observations between 2012 and 2019. We studied three birthweight indicators at the region and province levels in Peru; that is, we provided estimates with great granularity. We have highlighted strong subnational inequalities characterized by poor birthweight indicators in the Highlands, which also concentrates large poor and rural populations living at high altitude. None of the three indicators improved substantially between 2012-2019. These findings could also apply to other countries with a similar geographic, epidemiological, and sociodemographic profile, for example those in Andean Latin America. We expect that our work will spark interest in other countries and international health organizations to maximize the use of national birth registries to provide solid evidence to improve maternal and neonatal outcomes.

**Implications of all the available evidence**

Despite great improvement in maternal and neonatal health indicators globally (e.g., neonatal and under-five mortality), evidence on birthweight indicators at the national and subnational levels is limited and national time trends are harder to find. Our work contributes to close the gap, while signalling key areas in Peru where birthweight indicators need urgent attention, like places in the Highlands and with the worst socioeconomic indicators. Our results could apply to other countries and should receive attention by local and international health organizations.

Alt-text: Unlabelled box

## 1 Introduction

Even though low birthweight has been associated with poor sociodemographic and health outcomes during childhood and adolescence [1], [2], [3], [4], [5], [6], [7] as well as in adulthood, [6, [8], [9], [10], [11], [12], [13], [14], [15], [16], [17], [18], [19], [20], [21], [22], [23], [24], [25], [26], [27], [28]] mean birthweight and the prevalence of low birthweight and small for gestational age have not been well characterized globally nor at the national and subnational levels. Furthermore, their geographic and time trends have been poorly studied. Some countries have evidence from systematic reviews, [29], [30], [31] and others have studied few cities [32] or sub-populations [33] with interest on different outcomes and correlates according to data availability. [34], [35], [36], [37] There exists only one global endeavour that studied birthweight in all countries and territories; [38] nonetheless, evidence from low- and middle-income countries was limited precluding them to deliver solid evidence to monitor birthweight indicators along with time trends and subnational profiles.

Countries need accurate and current estimates of birthweight indicators to assess their progression towards global (e.g., WHO Global Nutrition Targets of 30% reduction in low birthweight by 2025 [39] and national goals. High-quality and granular data are needed to monitor the progress of low birthweight reduction globally, particularly to accurately assess the prevalence of new-born indicators in low- and middle-income countries. Evidence- and data-based interventions are available and frequently implemented to achieve the Global Nutrition World Health Assembly targets; however, these interventions may not work as expected in countries with diverse populations. Therefore, subnational trends can provide unique information to identify limitations on the implementation of current policies; similarly, subnational evidence offer the opportunity to focus resources to places where non-optimal indicators have remained steady for several years or may have worsened.

To provide this evidence for Peru, we analysed national birth registries between 2012 and 2019, and studied trends in mean birthweight and prevalence estimates of low birthweight and small for gestational age at the national and subnational levels; we also described the birthweight indicators by sociodemographic variables that have been well described in the literature. We provide unique evidence for national (e.g., Ministry of Health) and international (e.g., UNICEF) organizations working to improve birthweight in Peru, for which information at great granularity is needed and herein provided.

## 2 Methods

### 2.1 Data sources

This is a descriptive analysis. We analysed data of the Online Live Birth Certificate Registration System (*Sistema de Registro del Certificado de Nacido Vivo* in Spanish) in Peru between 2012 and 2019. [40, 41] This system was implemented in March 2012, covers all the country, and includes information from all healthcare systems (e.g., public and private). [40] It records all newborns right after birth and collects information about the mother (e.g., age and educational attainment), the newborn (e.g., sex and birthweight), and the pregnancy (e.g., gestational age). [40] The coverage of this system has improved since 2012, covering 12% in 2012, 37% in 2013, 53% in 2014, 72% in 2015, 80% in 2016, 84% in 2017 and 88% in 2018 of all projected births in Peru. [42] Data can be accessed upon request from the Ministry of Health.

### 2.2 Study setting

According to the World Bank as of 2019, Peru is an upper-middle income country with a gross domestic product of US$227 billion, a population of 32.5 million people and a life expectancy at birth of 76 years. Peru, located in South America, shares borders with the Pacific Ocean, Ecuador, Colombia, Brazil, Bolivia, and Chile. Peru is subdivided in three geographic levels: region > province > district. We summarised our findings at the region and province levels. Results were not summarised at the district level because in some of them there were very few births and there was large variability across districts, which would have prevented us from reaching meaningful conclusions. Peru is also divided in three natural geographic regions, namely Coast (next to the Pacific Ocean), Highlands (crossed by the Andes) and Amazon (rainforest next to Brazil). We also used these geographic areas to characterize the outcome variables. Peru has a tiered healthcare system. [43] The SIS (*Seguro Integral de Salud* in Spanish), which is run by the Ministry of Health, provides care for ~60% of the population and mainly targets people with limited financial resources; this is equivalent to the national funded model (Beveridge). [44] ESSALUD, which is run by the Ministry of Labour, provides care for people formally employed; this is equivalent to the social insurance model (Bismarck). [44] There are also private health insurance providers, which are commonly used by better-off people.

### 2.3 Study population

We conducted a complete-case analysis. In order to secure data quality, we applied the following cleaning criteria and plausibility ranges: i) excluded observations with birthweight below 500 g or above 5,500 g; ii) excluded observations with gestational age outside the range of 22-44 weeks; and iii) excluded observations in which maternal age was <9 years. When summarizing the results at the province level only, those provinces with fewer than 30 births were further excluded; we considered this threshold for data quality control because fewer than 30 births per year could be implausible at the province level, which is not the smallest administrative unit in Peru.

### 2.4 Definitions

We studied three outcomes: i) mean birthweight (in grams, g); ii) low birthweight (<2,500 g); and iii) small for gestational age (below the 10^th^ percentile). We used reference international growth curves. [45] These curves do not consider newborns <24 weeks of gestational age or ≥43 weeks; therefore, newborns outside this gestational age range were not included in the small for gestational age estimates. Birthweight and gestational age were recorded at the time of birth by health professionals.

We described the outcome variables in relation to time (i.e., year of birth) and geographic levels (i.e., region and province). Sex of the newborn was also used to describe the outcomes. Moreover, we presented the outcomes in relation to maternal educational attainment (none/initial, any primary education, incomplete secondary and complete secondary/higher education), and health insurance provider (SIS, ESSALUD and private).

Following an ecological approach at the region and province levels, we also described the outcome variables in relation to human development index (HDI, in 2019), [46] altitude above sea level (in meters), [46] proportion of the population living in poverty (presented as percentage, in 2018), [46] and proportion of people living in rural areas (presented as percentage, in 2017). [47] These four ecological variables were retrieved from national statistics at the province level. Because of lack of most recent data for population living in poverty and in rural areas, we assumed that these variables belonged to the year 2019.The ecological analysis was conducted for the year 2019 only.

### 2.5 Statistical analysis

First, birthweight was summarised as mean along with the 95% confidence interval (95% CI). The prevalence of low birthweight and small for gestational age was summarized as percentages, also with 95% CI. Clopper–Pearson exact CI was used for estimating the confidence intervals with PropCIs package within the R software, version 4.0.2. We used maps and time trend plots to characterize the spatial and temporal profiles of the three outcome variables. Second, we used equiplots to illustrate the differences in the outcome variables in terms of the three selected individual-level covariables: maternal education, age of the mother and health insurance provider. Third, we used scatterplots to show the relationship between the outcome variables and the selected determinants at the province level (ecological analysis); the scatterplots included the Pearson's correlation coefficient and p-value. The Pearson's correlation coefficient was not meant to inform about the magnitude of the association between the variables, but to rather serve as an aid to identify potential lineal patterns. Overall, this work is a descriptive analysis, thus the equiplots, scatterplots and the Pearson's correlation coefficients are used in a descriptive way rather than to signal or quantify the magnitude of the association between predictors and outcomes. We used R (version 4.0.2) for the analyses and figures.

### 2.6 Ethics

This work was waved of ethical approval by the Ethics Committee at Universidad Peruana Cayetano Heredia (UPCH), Lima, Peru.

### Role of funding source

The funder of the study had no role in study design, data collection, data analysis, data interpretation, or writing of the report. All authors had full access to the data in the study. All authors collectively had final responsibility for the decision to submit for publication and vouch for the data accuracy. The authors alone are responsible for the opinions in the manuscript, which do not necessarily represent those of their institutions.

## 3 Results

### 3.1 Study population

The registry originally included 2,933,482 births between 2012 and 2019. After applying our selection criteria, we included 2,927,761 births in the analysis (Supplementary Figure 1). Of these, 51.2% were boys and the mean gestational age was 38.7 weeks (standard deviation (SD) = 1.7). On average, mothers were below thirty years of age (mean = 27.7 years, SD = 6.9). Most births (98.2%) were singletons and 1.8% was double births. Similarly, 99.1% of all births occurred in a health facility and 0.6% were domiciliary (Table 1).

**Table 1:** Characteristics of the study population by year

Year	2012	2013	2014	2015	2016	2017	2018	2019
Sample size	72,849	214,687	307,833	416,995	459,355	479,186	492,827	484,029
Girls	48.6%	48.7%	48.7%	48.8%	48.8%	48.9%	48.9%	48.8%
Gestational age [mean (standard deviation)], weeks	38.8 (1.9)	38.8 (1.8)	38.8 (1.7)	38.7 (1.7)	38.7 (1.7)	38.7 (1.7)	38.7 (1.7)	38.6 (1.7)
Gestational age [10^th^ – 50^th^ – 90^th^ percentile], weeks	37-39-40	37-39-40	37-39-40	37-39-40	37-39-40	37-39-40	37-39-40	37-39-40
Maternal age [mean (standard deviation)], years	26.7 (6.9)	26.9 (6.9)	27.3 (6.9)	27.5 (6.9)	27.7 (6.9)	27.8 (6.9)	28.0 (6.9)	28.1 (6.9)
Singleton–multiple status								
Singleton	97.6%	97.8%	98.0%	98.1%	98.2%	98.3%	98.3%	98.3%
Double	2.3%	2.2%	2.0%	1.8%	1.8%	1.7%	1.7%	1.7%
Three or more	0.0%	0.0%	0.0%	0.0%	0.0%	0.0%	0.0%	0.0%
Place of birth								
House	0.2%	0.1%	0.2%	0.4%	0.6%	0.8%	0.7%	0.8%
Healthcare centre	99.7%	99.8%	99.6%	99.2%	99.0%	98.9%	98.9%	99.0%
Other	0.0%	0.1%	0.2%	0.4%	0.4%	0.4%	0.4%	0.2%
Mean birthweight (g)	3,287	3,273	3,270	3,260	3,258	3,261	3,268	3,262
Low birth weight prevalence (%)	6.9	6.7	6.4	6.4	6.2	6.1	6.0	6.2
Small for gestational age prevalence (%)	5.5	5.8	5.8	5.8	5.4	5.4	5.2	5.2

### 3.2 Geographical trends

**Figure 1:**
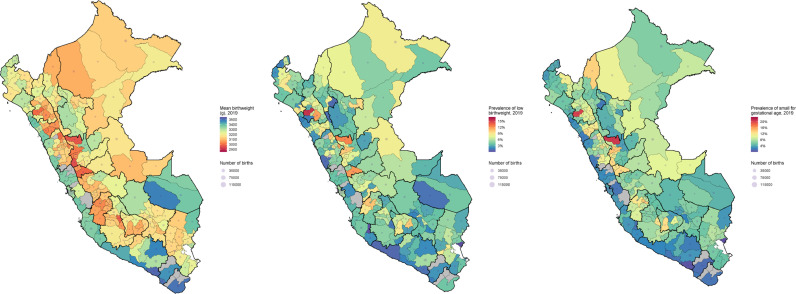
Geographic profile of mean birthweight (A), low birthweight prevalence (B) and small for gestational age prevalence (C) in Peru 2019. Colours indicate the mean or prevalence estimates. The size of the grey bubbles indicates the number of registered births, i.e., the study population or the denominator for prevalence estimates. Both colours and bubbles are at the province level. The dark black lines indicate the boundaries of the regions; NB Peru is divided in regions and these into provinces. Maps for all years in the study period are available in Supplementary Materials.

We observed consistently across years that mean birthweight was the lowest in the Highlands, while the highest means were found in the Coast (Figure 1, Supplementary Figure 6 and Table 2). Furthermore, in regions with both Coast and Highlands, the mean birthweight decreased from the Coast to the Highlands. For example, in 2019, in the Ancash region, Sihuas had the lowest mean birthweight (3,004 g (IC 95%: 2,954 g – 3,055 g); Highlands) whilst Huarmey had the highest (3,371 g (IC 95%: 3,319 g – 3,424 g); Coast); that is, a 367 g difference although both provinces belong to the same region (~354 km apart).

**Table 2:** Provinces with the lowest and highest mean birthweight across years and lowest and highest prevalence of low birthweight and small for gestational age across years.

	2012	2013	2014	2015	2016	2017	2018	2019
	**Mean birthweight (g)**							

Lowest	Cajamarca (Highlands) 2,919 (2,886–2,953)	Pasco (Highlands) 2,918 (2,823–3,013)	Hualgayoc (Highlands) 2,874 (2,697–3,051)	Sihuas (Highlands) 2,893 (2,831–2,956)	Antabamba (Highlands) 2,925 (2,787–3,063)	Castrovirreyna (Highlands) 2,903 (2,831–2,976)	Pasco (Highlands) 2,986 (2,967–3,005)	Lauricocha (Highlands) 2,954 (2,893–3,015)
Highest	Tacna (Coast) 3,427 (3,372–3,481)	Ilo (Coast) 3,537 (3,499–3,574)	Tacna (Coast) 3,533 (3,517–3,549)	Islay (Coast) 3,506 (3,467–3,545)	Islay (Coast) 3,545 (3,503–3,587)	Ilo (Coast) 3,507 (3,479–3,534)	Islay (Coast) 3,526 (3,479–3,573)	Islay (Coast) 3,516 (3,472–3,560)

	**Low birthweight prevalence (%)**							

Lowest	Ayabaca (Highlands) 1.9 (0.6–5.3)	Ascope (Coast) 0.6 (0.0–3.3)	Islay (Coast) 0.4 (0.0–2.1)	Caraveli (Coast) 1.1 (0.3–3.8)	Islay (Coast) 0.5 (0.1–1.7)	Caylloma (Highlands) 0.9 (0.6–1.6)	Islay (Coast) 0.8 (0.3–2.4)	Caraveli (Coast) 0.8 (0.0–4.6)
Highest	Cajamarca (Highlands) 16.6 (14.6–18.8)	Cajamarca (Highlands) 15.8 (14.7–17.0)	Datem del Marañon (Amazon) 14.1 (9.3–20.8)	Cajamarca (Highlands) 12.8 (12.0–13.6)	Pasco (Highlands) 12.3 (11.1–13.7)	Gran Chimu (Highlands) 12.8 (7.5–21.0)	Cajamarca (Highlands) 12.9 (12.1–13.6)	San Miguel (Highlands) 15.5 (10.6–22.2)

	**Small for gestational age (%)**							

Lowest	Tacna (Coast) 2.0 (1.1–3.8)	Bongara (Amazon) 1.3 (0.0–7.0)	Tacna (Coast) 1.7 (1.3–2.1)	Islay (Coast) 1.7 (0.9–3.4)	Huallaga (Amazon) 1.5 (0.5–4.3)	Manu (Amazon) 0.8 (0.0–4.5)	Huarmey (Coast) 0.9 (0.0–4.9)	Islay (Coast) 0.3 (0.0–1.6)
Highest	Ayabaca (Highlands) 15.5 (10.7–21.9)	Pasco (Highlands) 18.4 (12.1–27.0)	Hualgayoc (Highlands) 22.9 (12.1–39.0)	Sihuas (Highlands) 19.5 (14.7–25.4)	Sucre (Highlands) 19.2 (10.8–31.0)	Lauricocha (Highlands) 18.2 (13.5–24.0)	Grau (Highlands) 16.9 (10.5–26.0)	Marañon (Highlands) 20.8 (15.8–26.8)

Results are presented as mean or prevalence estimate along with the 95% confidence interval (95% CI)**.**

Regarding low birthweight prevalence, we observed a similar profile: higher prevalence estimates in the Highlands than in the Coast; also, in regions with both Coast and Highlands, provinces in the Highlands performed worse (Figure 1, Supplementary Figure 6 and Table 2). In regions with both Coast and Highlands, the region with the highest difference in low birthweight prevalence in 2019 was Ancash, where the lowest prevalence was in Huarmey (1.0% (IC 95%: 0.3% – 3.4%); Coast), whilst the highest was in Antonio Raymondi (11.4% (IC 95%: 5.9% – 21.0%); Highlands); although these are in the same region (~262 km apart), the prevalence was ten-fold (11.4% vs 1.0%) in Antonio Raymondi than in Huarmey.

Regarding the prevalence of small for gestational age, we observed a similar profile as for low birthweight; however, the magnitude of the prevalence estimates was higher for small for gestational age (Figure 1, Supplementary Figure 6 and Table 2). In some regions with both Coast and Highlands, the prevalence of small for gestational age was higher in the Highlands. For instance, in 2019 in La Libertad region the lowest prevalence was in Viru (2.1% (IC 95%: 1.4% – 3.0%); Coast), and the highest prevalence was in Julcan (13.0% (IC 95%: 9.3% – 17.9%); Highlands); despite being in the same region (~70 km apart), there was a 6-fold difference between these two provinces.

### 3.3 Time trends

**Figure 2:**
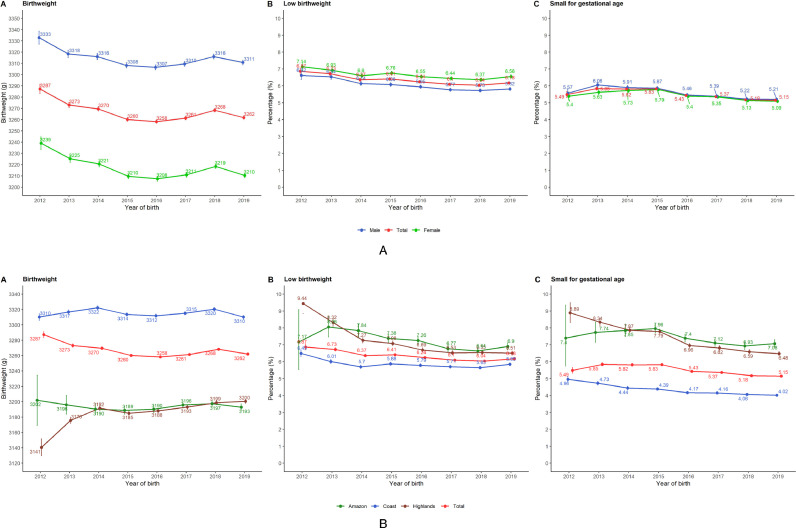
National time trends of mean birthweight, low birthweight prevalence and small for gestational age prevalence in Peru between 2012 and 2019, by sex (A) and natural region (B). Point estimates are shown along with the 95% confidence intervals.

Overall, the three birthweight indicators did not change substantially (Figure 2A and Table 1). Mean birthweight changed from 3,287 g in 2012 to 3,262 g in 2019. The prevalence of low birthweight went from 6.9% in 2012 to 6.2% in 2019; these numbers for small for gestational age were 5.5% and 5.2%, respectively. Mean birthweight was higher in boys, yet the prevalence of low birthweight was higher in girls; the prevalence of small for gestational age was virtually the same for boys and girls (Figure 2A).

When the mean birthweight, and prevalence of low birthweight and small for gestational age were split by natural region (i.e., Coast, Highlands and Amazon), the Coast had the best metrics across birthweight indicators and years (Figure 2B). Mean birthweight was consistently very similar between the Highlands and the Amazon, except for the last year when the Amazon performed slightly worse. Since 2016 the prevalence of low birth weight and small for gestational age was marginally higher in the Amazon than in the Highlands (Figure 2B).

The mean birthweight in most of the twenty-five regions in Peru showed a flat time trend, particularly in the last five years (Supplementary Figure 2). The top three largest increases were observed in Cajamarca (2,919 g in 2012 and 3,120 g in 2019), Pasco (2,918 g in 2013 and 3,079 g in 2019) and San Martin (3,148 g in 2012 and 3,231 g in 2019). Less often did we observe regions where the mean birthweight decreased: Tumbes (3,397 g in 2012 and 3,282 g in 2019), Ancash (3,322 g in 2013 and 3,238 g in 2019), and Ucayali (3,248 g in 2012 and 3,179 g in 2019). Across all twenty-five regions the mean birthweight was higher in boys (Supplementary Figure 2).

The prevalence of low birthweight remained stable or decreased slightly across the twenty-five regions (Supplementary Figure 3). The regions where we observed the largest decrease were Cajamarca (16.6% in 2012 to 8.2% in 2019), San Martin (12.0% in 2013 to 6.2% in 2019) and La Libertad (10.9% in 2012 to 6.2% in 2019). Conversely, we observed a marginal increase in Callao (4.9% in 2012 to 6.7% in 2019) and Ucayali (6.5% in 2013 to 7.7% in 2019). Although the difference was negligible, the prevalence of low birthweight was slightly higher in girls than boys (Supplementary Figure 3).

The prevalence of small for gestational age has not changed substantially throughout Peru (Supplementary Figure 4). The regions where we observed the largest decrease were Cajamarca (14.9% in 2012 to 9.1% in 2019), Junin (12.6% in 2013 to 8.0% in 2019) and San Martin (10.2% in 2013 to 5.8% in 2019). A negligible increase in the prevalence of small for gestational age was found in Ucayali (8.2% in 2013 to 8.7 % in 2019) and Ancash (5.2% in 2014 to 5.3% in 2019). The prevalence figures were almost identical in boys and girls (Supplementary Figure 4).

### 3.4 Equiplots and ecological analysis

**Figure 3:**
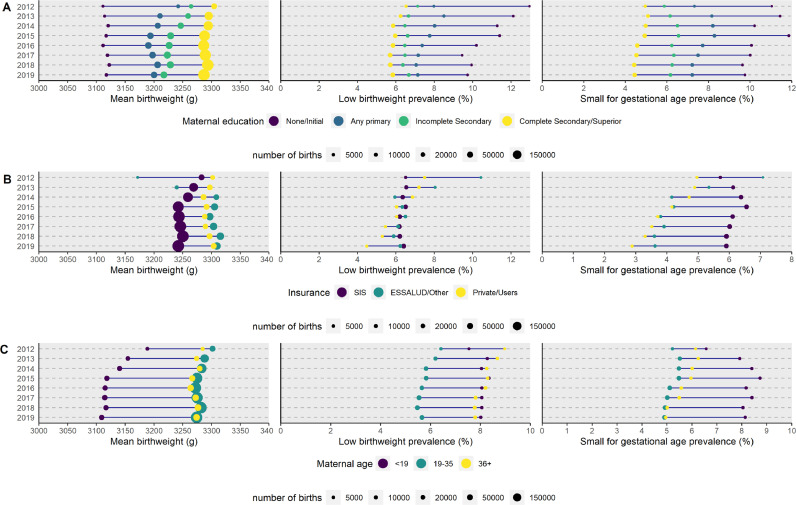
Equiplots of mean birthweight, low birthweight prevalence and small for gestational age prevalence according to maternal educational attainment (A), health insurance provider (B) and maternal age (C). The size of the bubbles is relative to the number of births.

We disaggregated the national information of three outcomes of our interest to examine the inequalities over time by maternal education, health insurance and age of the mother (Figure 3). Mothers with completed secondary education or higher education had babies with higher birthweight, while mothers with none or initial education had babies with lower birthweight across the study period (Figure 3A). Mothers with complete secondary or higher education had a smaller prevalence of babies with low birthweight and small for gestational weight (Figure 3A); in a similar vein, mothers who received private or ESSALUD care had babies with higher mean birthweight and experienced a smaller frequency of low birthweight and small for gestational age (Figure 3B). Across years and birthweight indicators, mothers who were <19 years of age had babies with the lowest mean birthweight and with the highest prevalence of small for gestational age; the prevalence of low birthweight was also high amongst these mother, though closely followed by mothers who were ≥36 years of age (Figure 3C).

The ecological analysis at the province level revealed similar findings. There was a positive correlation of mean birthweight with HDI, yet a negative correlation with altitude above sea level, poverty, and proportion of people living in rural areas (Supplementary Figure 5). The opposite pattern was observed with the prevalence estimates. For example, there was a negative correlation between small for gestational age and HDI, yet a positive correlation with altitude above sea level, poverty, and proportion of people living in rural areas (Supplementary Figure 5).

## 4 Discussion

### 4.1 Main results

In an analysis of eight years of the national birth registry in Peru, we did not observe substantial changes of time trends for mean birthweight, low birthweight prevalence and small for gestational age prevalence, at neither the national nor subnational levels. However, there were non-trivial geographic inequalities whereby provinces in the Highlands showed, consistently across years, worse birthweight indicators than provinces in the Coast. Our results pinpoint geographical disparities regarding birthweight indicators, which most likely occur in other low- and middle-income countries as well. Our work identified provinces that need urgent attention to improve birthweight indicators, i.e., those in the Highlands and with poor socioeconomic metrics. Interventions need to be tailored to meet the needs of each province while acknowledging the local cultural, health access and epidemiological profiles.

### 4.2 Strengths and limitations

We benefited from a national registry of births spanning eight years, which provided several relevant variables to describe trends in birthweight at the national and subnational levels in Peru. These births occurred in all regions and public and private health services in the country, allowing us to consolidate as much variability as possible in terms of geographical, cultural, sociodemographic and health access profiles. This work sets a milestone by providing useful estimates for researchers, local governments, policymakers, and organizations working to improve maternal and child health in Peru, especially in disadvantaged settings.

Nonetheless, we acknowledge limitations so that our findings can be interpreted accordingly. First, although all variables were registered by health professionals, we cannot guarantee that they all followed the same exact procedures and devices, for example, to estimate gestational age or to record birth weight, as it would have occurred in a controlled epidemiological study. This is a common limitation when analysing administrative data. This potential limitation could have implications for cross-province comparisons. If a province has only one health facility where the quality of information is poor, this province's estimates could be biased and affect comparative analyses across provinces. Another common limitation when working with administrative data is the lack of some variables. For example, we did not have information on whether the mother was a current/former smoker. Had we conducted an analytical work to quantify some associations and failed to adjusted for this (and other) variable, then this could have been an important limitation; nonetheless, this was a purely descriptive work in which the birthweight indicators were described by time, geography and some available socioeconomic variables. We do acknowledge there are some relevant correlates or risk factors that could not be used to stratify our results because of data availability. Second, we did not present information for all provinces across the observation period because the national registry has been implemented at a different pace throughout the country (hence the larger number of missing provinces in early years). This means that we cannot deliver estimates for all provinces in Peru, but, particularly for the last five years, we have covered most of the country pinpointing inequalities and signalling where interventions are most needed. It is also possible that some births that occurred at home, particularly in rural and remote areas, were missed from the registry. However, this registration system works hard to complement the online registration with manual entries; nonetheless, these manual entries clearly did not cover all provinces in early years of the national registry. Still, there are probably some births not included in the system, yet these should be few and should not have introduced substantial bias to our mean and prevalence estimates. Third, had we aimed to study the strength of the association between the correlates and the birthweight indicators, clearly the Pearson's correlation coefficient would have been an insufficient association metric. This work was intended as a descriptive piece, whereby means and prevalences were described in relation to time, geography and according to the level of socioeconomic variables. In this line, the Pearson's correlation coefficient was presented as a help to identify lineal relationships in the scatterplots and whether these were positive or negative. The Pearson's correlation coefficient was not used to quantify the magnitude of the association.

### 4.3 Potential explanations

We observed almost 50% reduction in the prevalence of low birthweight in some regions, like in Cajamarca, San Martin and La Libertad. One possible explanation is that these provinces are close one another and they share access routes which can be used to transfer high-risk women or those who need careful antenatal care. In La Libertad there are two maternal and child hospitals which could provide antenatal care to women from Cajamarca and San Martin. This is, of course, a bold hypothesis which deserves further investigation. Of note, there are no maternal and child hospitals in Cajamarca or San Martin.

Over the year there has been a decrease in the prevalence of low birthweight and small for gestational age amongst user of private health services. Overall, the underlying idea is that users of private services may have access to better care. Upon this general idea, we could draw two potential hypotheses. The first hypothesis is that, at the beginning of the study period, users of private services could afford this care but probably not a holistic care; that is, although women could afford a private clinic, they may not have the resources, knowledge or support to have a healthy pregnancy. The second hypothesis is that the quality of care provided by private health services has improved. Over the years the offer for private health services has increased substantially, and to keep competitive prices they needed to improve the quality of care provided, securing not only antenatal visits or institutional births but also complementary services like nutritional counselling.

The equiplots showed an inverse trend between the three birthweight indicators and maternal education, whereby mothers with fewer education years had newborns with lower birthweight, which is consistent with the literature. [48] Closely related, newborns delivered in public health facilities showed worse birthweight indicators. Presumably, mothers with low educational attainment would not have the resources to access private care, nor to secure a safe environment (e.g., adequate nutrition) for a healthy pregnancy. Overall, these two correlates -healthcare provider and maternal education- are not independent from one another and need structural changes to secure optimal antenatal care across sociodemographic strata. Undoubtedly, this is a multisectoral issue. For example, those working in healthcare could secure adequate antenatal care, those working with poor or vulnerable populations could secure access to what mothers need for a healthy pregnancy (e.g., diet, education, and prevention of acute diseases), and those working in transportation could secure safe routes and means to access timely antenatal and maternal healthcare, particularly in rural settings. [49] The equiplots also showed that the birthweight indicators would be worse for mothers aged <19 years; this observation agrees with the literature on the subject. [50, 51] Regarding low birthweight prevalence, the youngest mothers were closely followed by those aged 36+ years, which has also been documented before. [52] Both extremes of the reproductive age are considered at risk for adverse birth outcomes, and the prevalence of adverse birth outcomes have kept similar along time.

In our ecological analysis at the province level we showed that HDI was a protective factor against non-optimal birthweight indicators, while high altitude above sea level, poverty and rurality were risk factors for non-optimal birthweight indicators. These results are in line with previous studies showing that remote rural areas of the Amazon and the Highlands lagged behind in terms of progress achieved overtime by Peru for various neonatal and child health indicators. [53], [54], [55], [56] Also, our findings mirror those retrieved at the individual-level. Altitude above sea level has been found to have a negative correlation with birthweight. [57] Moreover, socioeconomic indicators in the Highlands are the worst in Peru (followed by the Amazon and Coast). [58] Most likely, there is an interaction effect between low socioeconomic status and altitude, though is it difficult –and beyond the scope of this work– to disentangle how much each of these contributes to poor birthweight indicators. From a public health perspective, it would not possible to intervene on the altitude above sea level, but improving socioeconomic conditions and access to maternal healthcare in these areas could be an alternative to improve birthweight indicators. Our work showed where these interventions are urgently needed and therefore inform public health officers where they need to focus their efforts. As hypothesized above regarding maternal education and access to public healthcare, the fact that the Highlands host worse-off populations could explain the findings and are consistent with the literature suggesting that maternal education, unemployment and poverty are associated with undernutrition in early life. [59] The Health Equity Report 2016 showed wealth, rural/urban residence and educational level as main factors influencing maternal health access and birthweight indicators across Latin America and the Caribbean. [60] Policies to secure homogenous antenatal care and opportunities to experience a healthy pregnancy are needed across subnational borders in Peru and other low- and middle-income countries, where geography should not be a synonym of poor healthcare access.

### 4.4 Public health implications

Reducing the incidence of low birthweight may have positive effects on the economy of LMICs. [61] There are several possible interventions to improve birthweight, namely improving antenatal education [62] and nutrition during pregnancy. [62], [63], [64], [65] Which is the most cost-effective, and which would render the best results in the Peruvian subnational context remains unanswered, yet our work provides arguments to justify this research and identified places where these interventions are most needed; that is, provinces in the Highlands and those with the worst sociodemographic profiles.

While the WHO Global Nutrition Targets established a 30% reduction on low birthweight by 2025, in 26 countries in LAC low birthweight rates only decreased 0.4% during 15 years. [66] This suggests that it would be difficult to meet the target at the national, and much more at the subnational level. To meet the WHO Global Nutrition Targets, the WHO recommendations are divided into country, community and personalized levels to take action to improve pregnant women nutritional status, healthcare access and environment. [39] Our results identified communities (provinces in the Highlands) were these interventions are needed to improve birthweight indicators.

The Peruvian JUNTOS programme, based on conditional cash transfers targeting rural people in the poorest communities, has been reported to increase the probability to attend antenatal care appointments for enrolled pregnant women. [67, 68] Our results helped to identify provinces where the implementation of a programme like JUNTOS could be reinforced or tightened, along with other interventions to promote adequate antenatal care.

Our results provide baseline information for future surveillance and monitoring frameworks of birthweight indicators in Peru. With a national and subnational analysis of recent (eight years) trends, we delivered evidence to set realistic and tailored targets to improve birthweight indicators in the short- and mid-term, even at the province level. National health authorities (i.e., decision makers), international organizations (e.g., UNICEF), and non-for-profit organizations could use these results to identify places to conduct their work to improve birthweight indicators; similarly, our results could foster discussions and evaluations of where birthweight indicators have not improved despite ongoing interventions. In this line, the results stratified by natural region (i.e., Coast, Highlands and Amazon) revealed an interesting finding that deserves careful consideration: the prevalence of low birthweight and small for gestational age were slightly higher in the Amazon than in the Highlands. Overall, our results pinpointed that in the Highlands we found more provinces with the worst birthweight indicators; however, in aggregate the Amazon and the Highlands are not far apart showing high prevalence of unfavourable birthweight profiles. Historically, the Highlands has been the region with worst socio-economic indicators and often recipient of interventions. Our results could suggest that the Amazon needs further care, and our analysis showed which provinces in the Amazon may need prioritization.

## 5 Conclusions

Peru has not experienced substantial changes in mean birthweight, prevalence of low birthweight and prevalence of small for gestational age between 2012 and 2019. Nonetheless, for these three metrics there were strong geographic inequalities whereby places in the Highlands performed worse than those in the Coast. This national and subnational analysis showed that interventions are most needed in the Highlands to improve poor birthweight profiles observed since 2012 in Peru.

## Contributions

All authors conceived the research question and analysis plan. RMC-L pooled and prepared the data. RMC-L, WCG-V, HGQ-P and KNC-T conducted the analysis and prepared the figures. RMC-L prepared the first draft of the manuscript with support from CT-M. RMC-L, KNC-T, WCG-V, HGQ-P, CT-M and LH provided critical scientific and editorial input to improve the manuscript. All authors approved the submitted version.

## Declaration of interests

No competing interests.

## Data sharing statement

All data herein analysed are open access and can be requested from the Ministry of Health in Peru: https://www.minsa.gob.pe/portada/transparencia/solicitud/frmFormulario.asp We provide the data herein analysed and the analysis code of data preparation, scatterplots and maps as supplementary materials to this paper.

Editor note: The Lancet Group takes a neutral position with respect to territorial claims in published maps and institutional affiliations

## Editor note

The Lancet Group takes a neutral position with respect to territorial claims in published maps and institutional affiliations.

## Appendix Supplementary materials

Image, application 1

Image, application 2
