# Supplementary material for: National and subnational trends of birthweight in Peru: Pooled analysis of 2,927,761 births between 2012 and 2019 from the national birth registry
Source: Lancet Reg Health Am. 2021 Jul 13;1:100017. doi: 10.1016/j.lana.2021.100017 (PMC8447569; doi:10.1016/j.lana.2021.100017)
Supplement: Supplementary file 1 [file mmc1.pdf]

National and subnational trends of birthweight in Peru: Pooled analysis of 2,927,761 births between 2012 and 2019 from the national birth registry

Corresponding author

Rodrigo M Carrillo-Larco, MD

CRONICAS Centre of Excellence in Chronic Diseases

Universidad Peruana Cayetano Heredia, Lima, Peru

rodrigo.carrillo@upch.pe

Supplementary Figure 1. Flowchart of data cleaning and inclusion criteria 3

Supplementary Table 1. Birthweight, low birthweight and small for gestational age by region, province, and year. 4

Supplementary Figure 2. Regional time trends of mean birthweight in Peru between 2012 and 2019, overall and by sex (point estimates are shown along with the 95% confidence intervals). 43

Supplementary Figure 3. Regional time trends of low birthweight prevalence in Peru between 2012 and 2019, overall and by sex (point estimates are shown along with the 95% confidence intervals). 44

Supplementary Figure 4. Regional time trends of small for gestational age prevalence in Peru between 2012 and 2019, overall and by sex (point estimates are shown along with the 95% confidence intervals). 45

Supplementary Figure 5. Scatterplots of mean birthweight, low birthweight prevalence and small for gestational age prevalence with human development index (HDI), altitude above sea level, poverty, and rurality.. 46

Supplementary Figure 6. Geographic profiles of mean birthweight, low birthweight prevalence and small for gestational age prevalence in Peru between 2012 and 2019. 47

Supplementary Figure 1. Flowchart of data cleaning and inclusion criteria

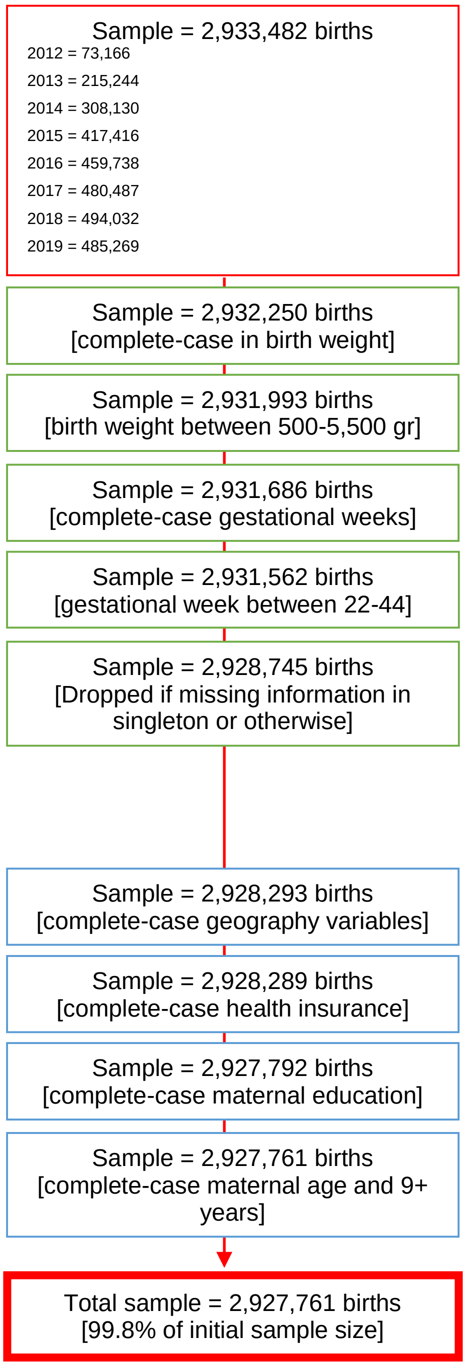

*In addition, for all plots and results at the *province* level, we dropped *provinces* in which there were fewer than 30 observations. For example, if *province* “X” in 2012 had fewer than 30 births to compute the mean birth weight or any prevalence estimate, *province* “X” was excluded.

Supplementary Table 1. Birthweight, low birthweight and small for gestational age by region, province, and year.

Year	Region	Province	Number of births	Birthweight (mean, 95% CI)	Low birthweight (%, 95% CI)	Small for gestational age (%, 95% CI)
2012	AMAZONAS	BAGUA	181	3173 (3094 - 3251)	9% (6% - 15%)	9% (6% - 15%)
2012	AMAZONAS	CHACHAPOYAS	227	3157 (3094 - 3220)	7% (4% - 11%)	7% (4% - 12%)
2012	AMAZONAS	LUYA	45	3193 (3075 - 3312)	2% (0% - 12%)	9% (2% - 21%)
2013	AMAZONAS	BAGUA	775	3245 (3207 - 3282)	6% (4% - 8%)	6% (4% - 8%)
2013	AMAZONAS	BONGARA	77	3303 (3218 - 3387)	1% (0% - 7%)	1% (0% - 7%)
2013	AMAZONAS	CHACHAPOYAS	1204	3139 (3109 - 3170)	8% (6% - 9%)	7% (6% - 9%)
2013	AMAZONAS	CONDORCANQUI	33	3098 (2901 - 3294)	12% (3% - 28%)	12% (3% - 28%)
2013	AMAZONAS	LUYA	106	3201 (3126 - 3277)	2% (0% - 7%)	11% (6% - 19%)
2013	AMAZONAS	RODRIGUEZ DE MENDOZA	125	3235 (3164 - 3306)	5% (2% - 10%)	6% (2% - 11%)
2013	AMAZONAS	UTCUBAMBA	581	3221 (3181 - 3260)	6% (4% - 8%)	5% (4% - 7%)
2014	AMAZONAS	BAGUA	851	3211 (3175 - 3248)	7% (6% - 9%)	6% (5% - 8%)
2014	AMAZONAS	BONGARA	143	3250 (3182 - 3318)	6% (2% - 11%)	6% (3% - 12%)
2014	AMAZONAS	CHACHAPOYAS	1422	3143 (3117 - 3168)	8% (7% - 10%)	8% (6% - 9%)
2014	AMAZONAS	CONDORCANQUI	117	3088 (2996 - 3180)	7% (3% - 13%)	10% (5% - 17%)
2014	AMAZONAS	LUYA	136	3174 (3103 - 3244)	5% (2% - 10%)	15% (10% - 23%)
2014	AMAZONAS	RODRIGUEZ DE MENDOZA	161	3260 (3196 - 3324)	1% (0% - 4%)	5% (2% - 10%)
2014	AMAZONAS	UTCUBAMBA	1039	3248 (3219 - 3277)	5% (4% - 7%)	7% (6% - 9%)
2015	AMAZONAS	BAGUA	1133	3232 (3203 - 3260)	6% (4% - 7%)	6% (5% - 8%)
2015	AMAZONAS	BONGARA	177	3214 (3158 - 3270)	1% (0% - 4%)	8% (5% - 14%)
2015	AMAZONAS	CHACHAPOYAS	1487	3175 (3149 - 3201)	8% (7% - 9%)	7% (6% - 9%)
2015	AMAZONAS	CONDORCANQUI	186	3072 (3003 - 3141)	6% (3% - 11%)	16% (11% - 22%)
2015	AMAZONAS	LUYA	146	3158 (3089 - 3227)	5% (2% - 10%)	13% (8% - 20%)
2015	AMAZONAS	RODRIGUEZ DE MENDOZA	158	3251 (3188 - 3313)	3% (1% - 7%)	6% (3% - 11%)
2015	AMAZONAS	UTCUBAMBA	1257	3235 (3209 - 3262)	5% (4% - 6%)	6% (5% - 7%)
2016	AMAZONAS	BAGUA	1136	3232 (3203 - 3261)	7% (5% - 8%)	6% (5% - 8%)
2016	AMAZONAS	BONGARA	212	3213 (3154 - 3272)	4% (2% - 7%)	4% (2% - 8%)

2016	AMAZONAS	CHACHAPOYAS	1621	3137 (3112 - 3163)	10% (8% - 11%)	6% (5% - 7%)
2016	AMAZONAS	CONDORCANQUI	401	3104 (3061 - 3147)	6% (4% - 9%)	14% (11% - 18%)
2016	AMAZONAS	LUYA	161	3153 (3095 - 3211)	1% (0% - 4%)	6% (3% - 10%)
2016	AMAZONAS	RODRIGUEZ DE MENDOZA	154	3291 (3231 - 3350)	1% (0% - 4%)	6% (3% - 11%)
2016	AMAZONAS	UTCUBAMBA	1307	3268 (3243 - 3293)	4% (3% - 6%)	5% (4% - 6%)
2017	AMAZONAS	BAGUA	1283	3203 (3176 - 3231)	7% (5% - 8%)	7% (6% - 9%)
2017	AMAZONAS	BONGARA	204	3182 (3128 - 3235)	3% (1% - 6%)	6% (3% - 11%)
2017	AMAZONAS	CHACHAPOYAS	1737	3179 (3155 - 3203)	7% (6% - 8%)	6% (5% - 7%)
2017	AMAZONAS	CONDORCANQUI	451	3104 (3059 - 3148)	6% (4% - 9%)	14% (11% - 18%)
2017	AMAZONAS	LUYA	167	3215 (3152 - 3277)	3% (1% - 7%)	7% (4% - 12%)
2017	AMAZONAS	RODRIGUEZ DE MENDOZA	166	3291 (3228 - 3355)	2% (0% - 5%)	2% (1% - 6%)
2017	AMAZONAS	UTCUBAMBA	1425	3269 (3245 - 3293)	5% (4% - 6%)	4% (3% - 6%)
2018	AMAZONAS	BAGUA	1193	3229 (3201 - 3257)	6% (4% - 7%)	6% (5% - 7%)
2018	AMAZONAS	BONGARA	173	3213 (3143 - 3282)	7% (4% - 12%)	7% (4% - 12%)
2018	AMAZONAS	CHACHAPOYAS	1726	3164 (3139 - 3188)	8% (7% - 9%)	6% (5% - 7%)
2018	AMAZONAS	CONDORCANQUI	426	3146 (3102 - 3190)	6% (4% - 9%)	13% (10% - 17%)
2018	AMAZONAS	LUYA	166	3219 (3157 - 3281)	4% (1% - 8%)	7% (3% - 12%)
2018	AMAZONAS	RODRIGUEZ DE MENDOZA	129	3204 (3129 - 3278)	4% (1% - 9%)	4% (1% - 9%)
2018	AMAZONAS	UTCUBAMBA	1387	3290 (3266 - 3314)	4% (3% - 5%)	4% (3% - 5%)
2019	AMAZONAS	BAGUA	1275	3211 (3184 - 3237)	6% (5% - 8%)	6% (5% - 8%)
2019	AMAZONAS	BONGARA	186	3190 (3131 - 3249)	4% (2% - 8%)	5% (2% - 9%)
2019	AMAZONAS	CHACHAPOYAS	1807	3157 (3133 - 3181)	8% (7% - 10%)	6% (5% - 8%)
2019	AMAZONAS	CONDORCANQUI	376	3088 (3039 - 3136)	8% (5% - 11%)	14% (10% - 17%)
2019	AMAZONAS	LUYA	114	3203 (3125 - 3282)	4% (1% - 9%)	7% (3% - 13%)
2019	AMAZONAS	RODRIGUEZ DE MENDOZA	100	3313 (3239 - 3388)	1% (0% - 5%)	2% (0% - 7%)
2019	AMAZONAS	UTCUBAMBA	1325	3275 (3249 - 3300)	4% (3% - 6%)	6% (4% - 7%)
2013	ANCASH	HUAYLAS	136	3142 (3069 - 3215)	8% (4% - 14%)	12% (7% - 18%)
2013	ANCASH	SANTA	413	3382 (3329 - 3435)	5% (3% - 8%)	3% (2% - 5%)
2014	ANCASH	HUARAZ	374	3194 (3151 - 3237)	4% (2% - 7%)	6% (4% - 8%)
2014	ANCASH	HUAYLAS	749	3150 (3122 - 3178)	3% (2% - 5%)	7% (5% - 9%)

2014	ANCASH	RECUAY	91	2963 (2887 - 3040)	11% (5% - 19%)	15% (9% - 24%)
2014	ANCASH	SANTA	3597	3340 (3322 - 3359)	7% (6% - 8%)	5% (4% - 5%)
2015	ANCASH	CARHUAZ	528	3078 (3045 - 3112)	6% (5% - 9%)	12% (9% - 15%)
2015	ANCASH	HUARAZ	3717	3126 (3110 - 3142)	8% (8% - 9%)	8% (7% - 9%)
2015	ANCASH	HUARI	542	3069 (3033 - 3104)	8% (6% - 11%)	10% (7% - 13%)
2015	ANCASH	HUAYLAS	800	3157 (3128 - 3185)	5% (4% - 7%)	10% (8% - 13%)
2015	ANCASH	POMABAMBA	306	3098 (3047 - 3149)	8% (5% - 11%)	7% (5% - 11%)
2015	ANCASH	RECUAY	140	3056 (2994 - 3117)	6% (2% - 11%)	14% (8% - 20%)
2015	ANCASH	SANTA	5659	3350 (3336 - 3364)	6% (5% - 7%)	4% (3% - 4%)
2015	ANCASH	SIHUAS	210	2893 (2831 - 2956)	12% (8% - 18%)	20% (14% - 26%)
2015	ANCASH	YUNGAY	368	3059 (3019 - 3100)	7% (4% - 10%)	12% (9% - 16%)
2016	ANCASH	ANTONIO RAIMONDI	58	3154 (3037 - 3270)	5% (1% - 14%)	12% (5% - 23%)
2016	ANCASH	ASUNCION	45	3070 (2969 - 3172)	4% (1% - 15%)	9% (2% - 21%)
2016	ANCASH	CARHUAZ	509	3089 (3057 - 3120)	4% (3% - 6%)	9% (7% - 12%)
2016	ANCASH	CARLOS FERMIN FITZCARRALD	120	2969 (2903 - 3036)	6% (2% - 12%)	14% (8% - 22%)
2016	ANCASH	HUARAZ	4196	3132 (3116 - 3147)	9% (8% - 9%)	7% (6% - 8%)
2016	ANCASH	HUARI	553	3086 (3051 - 3122)	8% (6% - 10%)	11% (9% - 14%)
2016	ANCASH	HUAYLAS	828	3150 (3121 - 3179)	4% (3% - 6%)	9% (7% - 11%)
2016	ANCASH	MARISCAL LUZURIAGA	74	3125 (3038 - 3211)	4% (1% - 11%)	9% (4% - 19%)
2016	ANCASH	POMABAMBA	380	3109 (3063 - 3156)	7% (5% - 10%)	7% (5% - 10%)
2016	ANCASH	RECUAY	170	3023 (2960 - 3086)	9% (5% - 14%)	14% (9% - 20%)
2016	ANCASH	SANTA	6427	3343 (3330 - 3357)	6% (5% - 6%)	3% (3% - 4%)
2016	ANCASH	SIHUAS	254	2984 (2926 - 3042)	9% (6% - 13%)	15% (11% - 20%)
2016	ANCASH	YUNGAY	474	3105 (3066 - 3145)	6% (4% - 9%)	9% (6% - 12%)
2017	ANCASH	ANTONIO RAIMONDI	78	3022 (2924 - 3121)	8% (3% - 16%)	15% (8% - 25%)
2017	ANCASH	ASUNCION	146	3091 (3015 - 3168)	7% (3% - 12%)	10% (5% - 16%)
2017	ANCASH	BOLOGNESI	36	2973 (2854 - 3091)	8% (2% - 22%)	8% (2% - 22%)
2017	ANCASH	CARHUAZ	520	3126 (3095 - 3157)	4% (2% - 6%)	5% (3% - 7%)

2017	ANCASH	CARLOS FERMIN FITZCARRALD	141	2970 (2896 - 3044)	11% (7% - 18%)	16% (10% - 23%)
2017	ANCASH	CASMA	236	3308 (3246 - 3370)	4% (2% - 8%)	3% (1% - 6%)
2017	ANCASH	HUARAZ	4196	3138 (3123 - 3153)	8% (7% - 9%)	7% (6% - 7%)
2017	ANCASH	HUARI	570	3110 (3077 - 3144)	7% (5% - 9%)	9% (6% - 11%)
2017	ANCASH	HUAYLAS	868	3150 (3121 - 3178)	6% (4% - 7%)	9% (7% - 11%)
2017	ANCASH	MARISCAL LUZURIAGA	82	3137 (3047 - 3227)	7% (3% - 15%)	10% (4% - 18%)
2017	ANCASH	PALLASCA	87	3061 (2983 - 3138)	6% (2% - 13%)	7% (3% - 14%)
2017	ANCASH	POMABAMBA	390	3125 (3082 - 3168)	7% (5% - 10%)	9% (7% - 13%)
2017	ANCASH	RECUAY	145	3072 (3002 - 3142)	5% (2% - 10%)	6% (3% - 11%)
2017	ANCASH	SANTA	7651	3336 (3324 - 3347)	5% (5% - 6%)	3% (3% - 3%)
2017	ANCASH	SIHUAS	248	3045 (2987 - 3102)	8% (5% - 12%)	15% (10% - 20%)
2017	ANCASH	YUNGAY	487	3132 (3096 - 3169)	4% (3% - 7%)	7% (5% - 10%)
2018	ANCASH	ANTONIO RAIMONDI	69	3087 (3004 - 3171)	3% (0% - 10%)	9% (3% - 18%)
2018	ANCASH	ASUNCION	120	3066 (3003 - 3129)	6% (2% - 12%)	12% (7% - 19%)
2018	ANCASH	BOLOGNESI	123	3014 (2948 - 3079)	7% (3% - 13%)	11% (6% - 17%)
2018	ANCASH	CARHUAZ	488	3127 (3091 - 3164)	5% (3% - 7%)	7% (5% - 10%)
2018	ANCASH	CARLOS FERMIN FITZCARRALD	119	3065 (2987 - 3143)	4% (1% - 10%)	9% (5% - 16%)
2018	ANCASH	CASMA	470	3310 (3272 - 3348)	1% (1% - 3%)	4% (2% - 6%)
2018	ANCASH	HUARAZ	4023	3115 (3099 - 3131)	9% (8% - 10%)	7% (6% - 8%)
2018	ANCASH	HUARI	617	3080 (3043 - 3117)	7% (5% - 10%)	9% (7% - 12%)
2018	ANCASH	HUARMEY	111	3456 (3387 - 3526)		1% (0% - 5%)
2018	ANCASH	HUAYLAS	814	3116 (3085 - 3146)	7% (5% - 9%)	11% (9% - 13%)
2018	ANCASH	MARISCAL LUZURIAGA	62	3080 (2993 - 3168)	5% (1% - 13%)	15% (7% - 26%)
2018	ANCASH	PALLASCA	132	3177 (3110 - 3243)	4% (1% - 9%)	6% (3% - 12%)
2018	ANCASH	POMABAMBA	385	3133 (3089 - 3176)	6% (4% - 9%)	8% (5% - 11%)
2018	ANCASH	RECUAY	117	3088 (3015 - 3161)	6% (2% - 12%)	10% (5% - 17%)
2018	ANCASH	SANTA	8586	3351 (3340 - 3363)	5% (5% - 5%)	3% (3% - 4%)
2018	ANCASH	SIHUAS	253	3060 (3010 - 3109)	5% (3% - 9%)	11% (7% - 15%)

2018	ANCASH	YUNGAY	474	3122 (3084 - 3160)	5% (4% - 8%)	7% (5% - 9%)
2019	ANCASH	ANTONIO RAIMONDI	70	3037 (2925 - 3148)	11% (5% - 21%)	11% (5% - 21%)
2019	ANCASH	ASUNCION	111	3114 (3039 - 3189)	9% (4% - 16%)	13% (7% - 20%)
2019	ANCASH	BOLOGNESI	107	3104 (3027 - 3181)	5% (2% - 11%)	10% (5% - 18%)
2019	ANCASH	CARHUAZ	485	3162 (3127 - 3197)	3% (2% - 5%)	8% (6% - 11%)
2019	ANCASH	CARLOS FERMIN FITZCARRALD	111	3105 (3039 - 3172)	6% (3% - 13%)	11% (6% - 18%)
2019	ANCASH	CASMA	485	3255 (3216 - 3294)	4% (2% - 6%)	7% (5% - 9%)
2019	ANCASH	HUARAZ	4085	3140 (3124 - 3155)	8% (7% - 9%)	7% (6% - 8%)
2019	ANCASH	HUARI	632	3141 (3107 - 3174)	5% (4% - 7%)	8% (6% - 10%)
2019	ANCASH	HUARMEY	208	3371 (3319 - 3424)	1% (0% - 3%)	2% (1% - 5%)
2019	ANCASH	HUAYLAS	716	3200 (3168 - 3231)	4% (3% - 6%)	7% (5% - 9%)
2019	ANCASH	MARISCAL LUZURIAGA	86	3156 (3077 - 3235)	3% (1% - 10%)	8% (3% - 16%)
2019	ANCASH	PALLASCA	114	3192 (3114 - 3271)	3% (1% - 7%)	6% (3% - 12%)
2019	ANCASH	POMABAMBA	361	3058 (3012 - 3103)	8% (5% - 11%)	9% (7% - 13%)
2019	ANCASH	RECUAY	124	3144 (3076 - 3212)	5% (2% - 10%)	11% (6% - 18%)
2019	ANCASH	SANTA	8316	3330 (3319 - 3341)	5% (4% - 5%)	3% (3% - 3%)
2019	ANCASH	SIHUAS	285	3004 (2954 - 3055)	10% (7% - 14%)	12% (8% - 16%)
2019	ANCASH	YUNGAY	455	3094 (3056 - 3132)	5% (3% - 8%)	10% (7% - 13%)
2012	APURIMAC	ABANCAY	740	3191 (3159 - 3223)	6% (4% - 7%)	6% (5% - 8%)
2012	APURIMAC	ANDAHUAYLAS	151	3202 (3123 - 3281)	6% (3% - 11%)	3% (1% - 8%)
2013	APURIMAC	ABANCAY	1917	3190 (3169 - 3211)	6% (5% - 7%)	5% (4% - 7%)
2013	APURIMAC	ANDAHUAYLAS	1071	3124 (3095 - 3153)	9% (7% - 11%)	9% (7% - 11%)
2014	APURIMAC	ABANCAY	2407	3219 (3200 - 3238)	6% (5% - 7%)	5% (4% - 6%)
2014	APURIMAC	ANDAHUAYLAS	2012	3180 (3158 - 3202)	7% (6% - 9%)	9% (7% - 10%)
2014	APURIMAC	COTABAMBAS	415	3093 (3055 - 3131)	5% (3% - 8%)	10% (7% - 13%)
2014	APURIMAC	GRAU	33	3139 (3005 - 3274)	6% (1% - 20%)	6% (1% - 20%)
2015	APURIMAC	ABANCAY	2686	3194 (3175 - 3213)	6% (5% - 7%)	4% (3% - 4%)
2015	APURIMAC	ANDAHUAYLAS	2265	3176 (3156 - 3196)	7% (6% - 8%)	7% (6% - 8%)
2015	APURIMAC	ANTABAMBA	43	3055 (2937 - 3173)	9% (3% - 22%)	12% (4% - 25%)

2015	APURIMAC	AYMARAES	44	3108 (2999 - 3217)	7% (1% - 19%)	7% (1% - 19%)
2015	APURIMAC	CHINCHEROS	208	3117 (3061 - 3173)	4% (2% - 8%)	9% (5% - 13%)
2015	APURIMAC	COTABAMBAS	706	3085 (3056 - 3114)	7% (5% - 9%)	9% (7% - 11%)
2015	APURIMAC	GRAU	134	3018 (2943 - 3094)	12% (7% - 19%)	13% (8% - 20%)
2016	APURIMAC	ABANCAY	2656	3197 (3179 - 3215)	6% (5% - 7%)	4% (3% - 4%)
2016	APURIMAC	ANDAHUAYLAS	2988	3163 (3147 - 3179)	6% (6% - 7%)	8% (7% - 9%)
2016	APURIMAC	ANTABAMBA	42	2925 (2787 - 3063)	12% (4% - 26%)	17% (7% - 31%)
2016	APURIMAC	AYMARAES	122	3029 (2952 - 3105)	9% (5% - 16%)	11% (6% - 18%)
2016	APURIMAC	CHINCHEROS	647	3133 (3103 - 3163)	4% (2% - 5%)	7% (5% - 9%)
2016	APURIMAC	COTABAMBAS	802	3094 (3066 - 3122)	7% (5% - 9%)	9% (7% - 11%)
2016	APURIMAC	GRAU	130	3077 (3002 - 3153)	12% (7% - 18%)	11% (6% - 17%)
2017	APURIMAC	ABANCAY	2717	3223 (3205 - 3242)	5% (5% - 6%)	3% (2% - 4%)
2017	APURIMAC	ANDAHUAYLAS	2955	3189 (3172 - 3206)	6% (5% - 7%)	8% (7% - 9%)
2017	APURIMAC	ANTABAMBA	60	3103 (3019 - 3187)	5% (1% - 14%)	7% (2% - 16%)
2017	APURIMAC	AYMARAES	134	3125 (3046 - 3203)	5% (2% - 10%)	7% (4% - 13%)
2017	APURIMAC	CHINCHEROS	635	3124 (3092 - 3157)	6% (4% - 8%)	9% (7% - 12%)
2017	APURIMAC	COTABAMBAS	750	3070 (3041 - 3098)	7% (5% - 9%)	11% (9% - 13%)
2017	APURIMAC	GRAU	123	3074 (3005 - 3143)	7% (3% - 12%)	8% (4% - 14%)
2018	APURIMAC	ABANCAY	2800	3239 (3221 - 3256)	6% (5% - 6%)	3% (2% - 4%)
2018	APURIMAC	ANDAHUAYLAS	2817	3190 (3172 - 3207)	6% (6% - 7%)	8% (7% - 9%)
2018	APURIMAC	AYMARAES	125	3158 (3090 - 3226)	5% (2% - 10%)	3% (1% - 8%)
2018	APURIMAC	CHINCHEROS	622	3183 (3152 - 3214)	3% (1% - 4%)	5% (4% - 7%)
2018	APURIMAC	COTABAMBAS	764	3132 (3106 - 3159)	3% (2% - 5%)	8% (6% - 10%)
2018	APURIMAC	GRAU	89	3042 (2944 - 3139)	9% (4% - 17%)	17% (10% - 26%)
2019	APURIMAC	ABANCAY	2736	3234 (3215 - 3253)	7% (6% - 8%)	3% (3% - 4%)
2019	APURIMAC	ANDAHUAYLAS	2710	3191 (3174 - 3209)	7% (6% - 8%)	7% (6% - 8%)
2019	APURIMAC	ANTABAMBA	37	3093 (2991 - 3195)	3% (0% - 14%)	5% (1% - 18%)
2019	APURIMAC	AYMARAES	93	3087 (3004 - 3170)	9% (4% - 16%)	13% (7% - 21%)
2019	APURIMAC	CHINCHEROS	576	3205 (3173 - 3236)	4% (2% - 6%)	6% (4% - 8%)
2019	APURIMAC	COTABAMBAS	741	3145 (3115 - 3174)	4% (3% - 6%)	7% (5% - 9%)

2019	APURIMAC	GRAU	78	3076 (2983 - 3170)	10% (5% - 19%)	10% (5% - 19%)
2012	AREQUIPA	AREQUIPA	949	3305 (3270 - 3340)	7% (5% - 8%)	5% (3% - 6%)
2013	AREQUIPA	AREQUIPA	12521	3310 (3300 - 3320)	7% (6% - 7%)	5% (4% - 5%)
2013	AREQUIPA	CAMANA	1018	3499 (3469 - 3530)	2% (1% - 3%)	2% (2% - 3%)
2013	AREQUIPA	CASTILLA	53	3305 (3155 - 3455)	8% (2% - 18%)	9% (3% - 21%)
2013	AREQUIPA	CAYLLOMA	292	3429 (3381 - 3476)	1% (0% - 3%)	2% (1% - 5%)
2013	AREQUIPA	ISLAY	42	3334 (3186 - 3482)	5% (1% - 16%)	7% (1% - 19%)
2014	AREQUIPA	AREQUIPA	20070	3329 (3322 - 3336)	5% (5% - 5%)	3% (3% - 4%)
2014	AREQUIPA	CAMANA	957	3492 (3464 - 3520)	2% (1% - 3%)	3% (2% - 4%)
2014	AREQUIPA	CARAVELI	195	3329 (3257 - 3401)	4% (1% - 7%)	6% (3% - 10%)
2014	AREQUIPA	CASTILLA	456	3293 (3250 - 3336)	5% (3% - 7%)	5% (3% - 7%)
2014	AREQUIPA	CAYLLOMA	1151	3380 (3355 - 3405)	3% (2% - 4%)	3% (2% - 4%)
2014	AREQUIPA	ISLAY	262	3514 (3466 - 3563)	0% (0% - 2%)	
2015	AREQUIPA	AREQUIPA	20612	3320 (3313 - 3327)	5% (5% - 6%)	4% (4% - 4%)
2015	AREQUIPA	CAMANA	982	3483 (3453 - 3513)	2% (1% - 3%)	3% (2% - 4%)
2015	AREQUIPA	CARAVELI	190	3361 (3302 - 3419)	1% (0% - 4%)	3% (1% - 7%)
2015	AREQUIPA	CASTILLA	405	3343 (3299 - 3387)	2% (1% - 4%)	4% (2% - 7%)
2015	AREQUIPA	CAYLLOMA	1196	3447 (3421 - 3473)	1% (1% - 2%)	3% (2% - 4%)
2015	AREQUIPA	CONDESUYOS	49	3232 (3128 - 3337)		4% (0% - 14%)
2015	AREQUIPA	ISLAY	458	3506 (3467 - 3545)	1% (0% - 3%)	2% (1% - 3%)
2016	AREQUIPA	AREQUIPA	19745	3318 (3311 - 3325)	5% (5% - 6%)	3% (3% - 4%)
2016	AREQUIPA	CAMANA	1072	3502 (3475 - 3529)	2% (1% - 3%)	2% (1% - 3%)
2016	AREQUIPA	CARAVELI	153	3373 (3307 - 3439)	3% (1% - 7%)	3% (1% - 7%)
2016	AREQUIPA	CASTILLA	445	3286 (3241 - 3331)	6% (4% - 8%)	6% (4% - 8%)
2016	AREQUIPA	CAYLLOMA	1231	3437 (3412 - 3462)	1% (1% - 2%)	2% (1% - 3%)
2016	AREQUIPA	CONDESUYOS	65	3367 (3275 - 3459)		
2016	AREQUIPA	ISLAY	422	3545 (3503 - 3587)	0% (0% - 2%)	2% (1% - 3%)
2017	AREQUIPA	AREQUIPA	19261	3326 (3319 - 3334)	5% (5% - 6%)	3% (3% - 4%)
2017	AREQUIPA	CAMANA	1071	3469 (3443 - 3496)	2% (1% - 3%)	2% (2% - 3%)
2017	AREQUIPA	CARAVELI	130	3380 (3314 - 3447)		2% (0% - 5%)

2017	AREQUIPA	CASTILLA	418	3315 (3269 - 3361)	4% (2% - 6%)	4% (2% - 6%)
2017	AREQUIPA	CAYLLOMA	1379	3484 (3462 - 3506)	1% (1% - 2%)	1% (1% - 2%)
2017	AREQUIPA	CONDESUYOS	77	3354 (3262 - 3446)	1% (0% - 7%)	1% (0% - 7%)
2017	AREQUIPA	ISLAY	423	3506 (3458 - 3555)	2% (1% - 4%)	2% (1% - 4%)
2017	AREQUIPA	LA UNION	35	2985 (2816 - 3154)	11% (3% - 27%)	17% (7% - 34%)
2018	AREQUIPA	AREQUIPA	19214	3320 (3313 - 3327)	5% (5% - 6%)	4% (3% - 4%)
2018	AREQUIPA	CAMANA	1050	3471 (3443 - 3500)	2% (1% - 2%)	2% (1% - 3%)
2018	AREQUIPA	CARAVELI	165	3344 (3274 - 3413)	1% (0% - 4%)	2% (1% - 6%)
2018	AREQUIPA	CASTILLA	398	3334 (3291 - 3378)	3% (1% - 5%)	4% (3% - 7%)
2018	AREQUIPA	CAYLLOMA	1331	3457 (3432 - 3481)	2% (1% - 3%)	2% (1% - 3%)
2018	AREQUIPA	CONDESUYOS	80	3357 (3259 - 3456)	4% (1% - 11%)	4% (1% - 11%)
2018	AREQUIPA	ISLAY	364	3526 (3479 - 3573)	1% (0% - 2%)	1% (0% - 3%)
2018	AREQUIPA	LA UNION	60	3090 (2971 - 3208)	8% (3% - 18%)	12% (5% - 23%)
2019	AREQUIPA	AREQUIPA	18449	3311 (3304 - 3319)	6% (5% - 6%)	3% (3% - 3%)
2019	AREQUIPA	CAMANA	1038	3470 (3441 - 3499)	2% (1% - 3%)	2% (1% - 3%)
2019	AREQUIPA	CARAVELI	120	3425 (3356 - 3494)	1% (0% - 5%)	2% (1% - 7%)
2019	AREQUIPA	CASTILLA	391	3310 (3262 - 3358)	4% (2% - 6%)	4% (3% - 7%)
2019	AREQUIPA	CAYLLOMA	1373	3475 (3450 - 3501)	2% (1% - 3%)	2% (1% - 3%)
2019	AREQUIPA	CONDESUYOS	76	3248 (3158 - 3339)	3% (0% - 9%)	3% (0% - 9%)
2019	AREQUIPA	ISLAY	352	3516 (3472 - 3560)	1% (0% - 3%)	0% (0% - 2%)
2019	AREQUIPA	LA UNION	66	3141 (3023 - 3258)	6% (2% - 15%)	6% (2% - 15%)
2013	AYACUCHO	CANGALLO	261	3050 (2999 - 3102)	7% (4% - 11%)	13% (10% - 18%)
2013	AYACUCHO	HUAMANGA	3965	3113 (3097 - 3129)	9% (8% - 10%)	10% (9% - 11%)
2013	AYACUCHO	HUANCA SANCOS	61	3095 (3005 - 3185)	7% (2% - 16%)	8% (3% - 18%)
2013	AYACUCHO	HUANTA	944	3206 (3179 - 3234)	4% (3% - 5%)	6% (4% - 8%)
2013	AYACUCHO	LA MAR	576	3295 (3255 - 3336)	4% (3% - 6%)	8% (6% - 10%)
2013	AYACUCHO	LUCANAS	371	3128 (3086 - 3169)	6% (4% - 9%)	10% (7% - 14%)
2013	AYACUCHO	PARINACOCHAS	372	3109 (3068 - 3151)	6% (4% - 9%)	10% (7% - 14%)
2013	AYACUCHO	SUCRE	63	3029 (2930 - 3128)	10% (4% - 20%)	17% (9% - 29%)
2013	AYACUCHO	VICTOR FAJARDO	43	3074 (2941 - 3206)	7% (1% - 19%)	9% (3% - 22%)

2013	AYACUCHO	VILCAS HUAMAN	149	3008 (2942 - 3073)	9% (5% - 14%)	17% (11% - 24%)
2014	AYACUCHO	CANGALLO	432	3069 (3028 - 3110)	10% (8% - 14%)	11% (8% - 14%)
2014	AYACUCHO	HUAMANGA	5364	3131 (3117 - 3144)	8% (8% - 9%)	8% (8% - 9%)
2014	AYACUCHO	HUANCA SANCOS	80	3088 (3006 - 3170)	4% (1% - 11%)	8% (3% - 16%)
2014	AYACUCHO	HUANTA	1370	3220 (3198 - 3243)	3% (3% - 5%)	5% (4% - 7%)
2014	AYACUCHO	LA MAR	1100	3214 (3187 - 3242)	5% (4% - 7%)	9% (7% - 11%)
2014	AYACUCHO	LUCANAS	455	3129 (3089 - 3169)	6% (4% - 9%)	9% (7% - 13%)
2014	AYACUCHO	PARINACOCHAS	455	3108 (3071 - 3145)	7% (4% - 9%)	9% (7% - 12%)
2014	AYACUCHO	SUCRE	55	3090 (2966 - 3214)	7% (2% - 18%)	9% (3% - 20%)
2014	AYACUCHO	VICTOR FAJARDO	66	3101 (3021 - 3181)	5% (1% - 13%)	8% (3% - 17%)
2014	AYACUCHO	VILCAS HUAMAN	155	2994 (2929 - 3060)	8% (5% - 14%)	19% (13% - 26%)
2015	AYACUCHO	CANGALLO	465	3075 (3036 - 3114)	8% (6% - 11%)	10% (7% - 13%)
2015	AYACUCHO	HUAMANGA	6494	3136 (3124 - 3148)	8% (7% - 9%)	9% (8% - 9%)
2015	AYACUCHO	HUANCA SANCOS	81	3168 (3077 - 3260)	7% (3% - 15%)	7% (3% - 15%)
2015	AYACUCHO	HUANTA	1617	3252 (3232 - 3272)	3% (2% - 3%)	4% (3% - 5%)
2015	AYACUCHO	LA MAR	1485	3217 (3194 - 3240)	4% (3% - 5%)	8% (7% - 10%)
2015	AYACUCHO	LUCANAS	495	3107 (3069 - 3145)	5% (4% - 8%)	14% (11% - 17%)
2015	AYACUCHO	PARINACOCHAS	480	3119 (3079 - 3159)	6% (4% - 9%)	10% (7% - 13%)
2015	AYACUCHO	PAUCAR DEL SARA SARA	66	3229 (3141 - 3317)	3% (0% - 11%)	3% (0% - 11%)
2015	AYACUCHO	SUCRE	70	3112 (3029 - 3196)	1% (0% - 8%)	13% (6% - 23%)
2015	AYACUCHO	VICTOR FAJARDO	55	3004 (2886 - 3122)	11% (4% - 22%)	11% (4% - 22%)
2015	AYACUCHO	VILCAS HUAMAN	149	3047 (2979 - 3114)	9% (5% - 14%)	13% (8% - 19%)
2016	AYACUCHO	CANGALLO	494	3063 (3023 - 3103)	7% (5% - 9%)	7% (5% - 10%)
2016	AYACUCHO	HUAMANGA	6825	3151 (3139 - 3162)	8% (7% - 9%)	8% (7% - 9%)
2016	AYACUCHO	HUANCA SANCOS	65	3069 (2962 - 3175)	6% (2% - 15%)	9% (3% - 19%)
2016	AYACUCHO	HUANTA	1581	3233 (3212 - 3254)	4% (3% - 5%)	5% (4% - 6%)
2016	AYACUCHO	LA MAR	1511	3225 (3202 - 3247)	4% (3% - 5%)	8% (6% - 9%)
2016	AYACUCHO	LUCANAS	501	3118 (3082 - 3155)	5% (3% - 8%)	12% (9% - 15%)
2016	AYACUCHO	PARINACOCHAS	423	3158 (3117 - 3199)	6% (4% - 9%)	7% (5% - 10%)
2016	AYACUCHO	PAUCAR DEL SARA SARA	59	3119 (3014 - 3225)	7% (2% - 16%)	8% (3% - 19%)

2016	AYACUCHO	SUCRE	52	3054 (2950 - 3157)	4% (0% - 13%)	19% (10% - 33%)
2016	AYACUCHO	VICTOR FAJARDO	59	3097 (3002 - 3191)	3% (0% - 12%)	7% (2% - 16%)
2016	AYACUCHO	VILCAS HUAMAN	146	3084 (3024 - 3143)	5% (2% - 10%)	10% (5% - 16%)
2017	AYACUCHO	CANGALLO	509	3077 (3045 - 3110)	6% (4% - 8%)	7% (5% - 10%)
2017	AYACUCHO	HUAMANGA	7095	3142 (3131 - 3154)	8% (7% - 8%)	7% (7% - 8%)
2017	AYACUCHO	HUANCA SANCOS	53	3071 (2970 - 3172)	2% (0% - 10%)	6% (1% - 16%)
2017	AYACUCHO	HUANTA	1670	3211 (3191 - 3232)	5% (4% - 6%)	5% (4% - 6%)
2017	AYACUCHO	LA MAR	1842	3282 (3261 - 3304)	4% (3% - 5%)	6% (5% - 7%)
2017	AYACUCHO	LUCANAS	476	3172 (3132 - 3211)	5% (3% - 8%)	7% (4% - 9%)
2017	AYACUCHO	PARINACOCHAS	402	3209 (3168 - 3250)	4% (2% - 6%)	7% (5% - 10%)
2017	AYACUCHO	PAUCAR DEL SARA SARA	62	3127 (3036 - 3219)	6% (2% - 16%)	5% (1% - 13%)
2017	AYACUCHO	SUCRE	73	3061 (2964 - 3158)	4% (1% - 12%)	5% (2% - 13%)
2017	AYACUCHO	VICTOR FAJARDO	53	3092 (3001 - 3183)	2% (0% - 10%)	6% (1% - 16%)
2017	AYACUCHO	VILCAS HUAMAN	124	3063 (3002 - 3124)	2% (1% - 7%)	9% (5% - 15%)
2018	AYACUCHO	CANGALLO	498	3050 (3010 - 3090)	9% (7% - 12%)	12% (10% - 15%)
2018	AYACUCHO	HUAMANGA	6909	3168 (3156 - 3179)	8% (7% - 8%)	6% (6% - 7%)
2018	AYACUCHO	HUANCA SANCOS	63	3118 (3002 - 3235)	6% (2% - 15%)	8% (3% - 18%)
2018	AYACUCHO	HUANTA	1584	3231 (3212 - 3251)	3% (3% - 4%)	5% (4% - 6%)
2018	AYACUCHO	LA MAR	1660	3299 (3277 - 3321)	4% (3% - 5%)	6% (5% - 7%)
2018	AYACUCHO	LUCANAS	460	3178 (3142 - 3215)	4% (2% - 6%)	9% (7% - 12%)
2018	AYACUCHO	PARINACOCHAS	394	3211 (3167 - 3256)	3% (2% - 6%)	8% (5% - 11%)
2018	AYACUCHO	PAUCAR DEL SARA SARA	40	3154 (3019 - 3290)	8% (2% - 20%)	8% (2% - 20%)
2018	AYACUCHO	SUCRE	61	3086 (2990 - 3182)	3% (0% - 11%)	13% (6% - 24%)
2018	AYACUCHO	VICTOR FAJARDO	61	3028 (2943 - 3114)	3% (0% - 11%)	11% (5% - 22%)
2018	AYACUCHO	VILCAS HUAMAN	102	3035 (2945 - 3124)	12% (6% - 20%)	11% (6% - 18%)
2019	AYACUCHO	CANGALLO	417	3108 (3066 - 3151)	5% (3% - 8%)	6% (4% - 9%)
2019	AYACUCHO	HUAMANGA	6812	3185 (3173 - 3197)	7% (7% - 8%)	7% (6% - 7%)
2019	AYACUCHO	HUANCA SANCOS	46	3141 (3031 - 3251)	4% (1% - 15%)	7% (1% - 18%)
2019	AYACUCHO	HUANTA	1592	3235 (3214 - 3256)	3% (2% - 4%)	5% (4% - 6%)
2019	AYACUCHO	LA MAR	1566	3285 (3262 - 3308)	4% (3% - 5%)	5% (4% - 6%)

2019	AYACUCHO	LUCANAS	438	3192 (3154 - 3231)	5% (3% - 7%)	7% (5% - 10%)
2019	AYACUCHO	PARINACOCHAS	426	3165 (3123 - 3206)	4% (3% - 7%)	8% (6% - 12%)
2019	AYACUCHO	PAUCAR DEL SARA SARA	47	3176 (3068 - 3285)	2% (0% - 11%)	6% (1% - 18%)
2019	AYACUCHO	SUCRE	72	3056 (2980 - 3133)	1% (0% - 7%)	12% (6% - 22%)
2019	AYACUCHO	VICTOR FAJARDO	48	3122 (3018 - 3226)	6% (1% - 17%)	6% (1% - 17%)
2019	AYACUCHO	VILCAS HUAMAN	88	2983 (2910 - 3055)	6% (2% - 13%)	6% (2% - 13%)
2012	CAJAMARCA	CAJAMARCA	1175	2919 (2886 - 2953)	17% (15% - 19%)	15% (13% - 17%)
2013	CAJAMARCA	CAJABAMBA	476	3050 (3008 - 3092)	6% (4% - 9%)	9% (7% - 12%)
2013	CAJAMARCA	CAJAMARCA	4019	2927 (2910 - 2944)	16% (15% - 17%)	16% (15% - 17%)
2013	CAJAMARCA	CHOTA	391	3076 (3029 - 3124)	9% (7% - 13%)	11% (8% - 15%)
2013	CAJAMARCA	JAEN	807	3239 (3205 - 3273)	6% (4% - 8%)	8% (6% - 10%)
2013	CAJAMARCA	SAN IGNACIO	117	3197 (3122 - 3272)	3% (1% - 9%)	8% (4% - 14%)
2013	CAJAMARCA	SAN MARCOS	235	3109 (3052 - 3166)	6% (3% - 9%)	10% (7% - 15%)
2014	CAJAMARCA	CAJABAMBA	837	3135 (3102 - 3169)	7% (5% - 9%)	11% (9% - 13%)
2014	CAJAMARCA	CAJAMARCA	5034	2993 (2977 - 3008)	14% (13% - 15%)	15% (14% - 16%)
2014	CAJAMARCA	CELENDIN	445	3148 (3107 - 3189)	4% (3% - 7%)	10% (8% - 14%)
2014	CAJAMARCA	CHOTA	1436	3075 (3052 - 3098)	9% (7% - 11%)	11% (9% - 13%)
2014	CAJAMARCA	CUTERVO	798	3090 (3057 - 3124)	9% (7% - 11%)	11% (8% - 13%)
2014	CAJAMARCA	HUALGAYOC	35	2874 (2697 - 3051)	6% (1% - 19%)	23% (10% - 40%)
2014	CAJAMARCA	JAEN	1808	3203 (3179 - 3228)	8% (7% - 9%)	10% (8% - 11%)
2014	CAJAMARCA	SAN IGNACIO	442	3178 (3139 - 3218)	3% (2% - 5%)	10% (7% - 13%)
2014	CAJAMARCA	SAN MARCOS	338	3035 (2985 - 3085)	10% (7% - 13%)	16% (12% - 20%)
2014	CAJAMARCA	SANTA CRUZ	62	3050 (2937 - 3163)	11% (5% - 22%)	13% (6% - 24%)
2015	CAJAMARCA	CAJABAMBA	932	3169 (3140 - 3198)	5% (4% - 6%)	8% (7% - 10%)
2015	CAJAMARCA	CAJAMARCA	6380	3014 (3001 - 3028)	13% (12% - 14%)	13% (12% - 14%)
2015	CAJAMARCA	CELENDIN	512	3158 (3120 - 3197)	5% (4% - 8%)	12% (9% - 15%)
2015	CAJAMARCA	CHOTA	1557	3070 (3047 - 3094)	9% (7% - 10%)	11% (10% - 13%)
2015	CAJAMARCA	CONTUMAZA	55	3156 (3061 - 3252)	2% (0% - 10%)	5% (1% - 15%)
2015	CAJAMARCA	CUTERVO	962	3122 (3094 - 3150)	7% (6% - 9%)	11% (9% - 13%)
2015	CAJAMARCA	HUALGAYOC	618	3048 (3014 - 3081)	8% (6% - 11%)	15% (12% - 18%)

2015	CAJAMARCA	JAEN	2439	3200 (3181 - 3220)	7% (6% - 8%)	10% (8% - 11%)
2015	CAJAMARCA	SAN IGNACIO	604	3174 (3140 - 3207)	4% (3% - 6%)	10% (8% - 13%)
2015	CAJAMARCA	SAN MARCOS	310	3074 (3021 - 3126)	6% (4% - 9%)	12% (9% - 16%)
2015	CAJAMARCA	SANTA CRUZ	155	3171 (3113 - 3230)	3% (1% - 6%)	8% (5% - 14%)
2016	CAJAMARCA	CAJABAMBA	905	3175 (3146 - 3205)	4% (3% - 5%)	8% (7% - 10%)
2016	CAJAMARCA	CAJAMARCA	7283	3031 (3018 - 3043)	12% (12% - 13%)	11% (10% - 11%)
2016	CAJAMARCA	CELENDIN	548	3153 (3115 - 3191)	5% (4% - 8%)	9% (7% - 12%)
2016	CAJAMARCA	CHOTA	1784	3086 (3066 - 3107)	8% (6% - 9%)	10% (9% - 12%)
2016	CAJAMARCA	CONTUMAZA	159	3138 (3074 - 3203)	6% (3% - 10%)	6% (3% - 11%)
2016	CAJAMARCA	CUTERVO	1096	3093 (3067 - 3119)	8% (6% - 9%)	9% (8% - 11%)
2016	CAJAMARCA	HUALGAYOC	759	3022 (2995 - 3050)	8% (6% - 10%)	13% (10% - 15%)
2016	CAJAMARCA	JAEN	3015	3229 (3212 - 3246)	6% (5% - 6%)	9% (8% - 10%)
2016	CAJAMARCA	SAN IGNACIO	839	3180 (3150 - 3210)	5% (4% - 7%)	9% (7% - 11%)
2016	CAJAMARCA	SAN MARCOS	343	3074 (3027 - 3122)	8% (5% - 11%)	11% (8% - 15%)
2016	CAJAMARCA	SAN MIGUEL	188	2934 (2880 - 2989)	10% (6% - 15%)	14% (10% - 20%)
2016	CAJAMARCA	SAN PABLO	142	3067 (3000 - 3134)	6% (2% - 11%)	11% (6% - 17%)
2016	CAJAMARCA	SANTA CRUZ	181	3190 (3120 - 3260)	3% (1% - 7%)	3% (1% - 7%)
2017	CAJAMARCA	CAJABAMBA	900	3183 (3155 - 3211)	4% (3% - 5%)	7% (5% - 9%)
2017	CAJAMARCA	CAJAMARCA	7570	3036 (3024 - 3048)	12% (11% - 13%)	12% (11% - 12%)
2017	CAJAMARCA	CELENDIN	606	3164 (3128 - 3199)	5% (4% - 8%)	10% (8% - 13%)
2017	CAJAMARCA	CHOTA	1952	3100 (3079 - 3120)	8% (7% - 10%)	11% (9% - 12%)
2017	CAJAMARCA	CONTUMAZA	162	3122 (3053 - 3192)	7% (4% - 13%)	11% (7% - 17%)
2017	CAJAMARCA	CUTERVO	1292	3126 (3102 - 3150)	7% (5% - 8%)	9% (8% - 11%)
2017	CAJAMARCA	HUALGAYOC	783	3050 (3021 - 3078)	7% (5% - 9%)	14% (11% - 16%)
2017	CAJAMARCA	JAEN	3327	3222 (3205 - 3238)	6% (5% - 7%)	8% (7% - 9%)
2017	CAJAMARCA	SAN IGNACIO	819	3191 (3162 - 3219)	4% (2% - 5%)	9% (7% - 11%)
2017	CAJAMARCA	SAN MARCOS	421	3024 (2982 - 3065)	7% (4% - 9%)	14% (11% - 18%)
2017	CAJAMARCA	SAN MIGUEL	162	3066 (3010 - 3122)	4% (2% - 9%)	9% (5% - 15%)
2017	CAJAMARCA	SAN PABLO	116	3152 (3081 - 3224)	3% (1% - 9%)	9% (5% - 16%)
2017	CAJAMARCA	SANTA CRUZ	173	3156 (3099 - 3212)	3% (1% - 7%)	4% (2% - 8%)

2018	CAJAMARCA	CAJABAMBA	859	3240 (3210 - 3269)	4% (3% - 5%)	6% (4% - 7%)
2018	CAJAMARCA	CAJAMARCA	7350	3036 (3023 - 3048)	13% (12% - 14%)	11% (10% - 12%)
2018	CAJAMARCA	CELENDIN	572	3131 (3095 - 3166)	5% (3% - 7%)	9% (7% - 11%)
2018	CAJAMARCA	CHOTA	1964	3117 (3097 - 3137)	7% (6% - 9%)	9% (7% - 10%)
2018	CAJAMARCA	CONTUMAZA	134	3156 (3090 - 3221)	3% (1% - 7%)	8% (4% - 14%)
2018	CAJAMARCA	CUTERVO	1396	3155 (3130 - 3179)	7% (6% - 9%)	8% (7% - 10%)
2018	CAJAMARCA	HUALGAYOC	767	3051 (3021 - 3081)	8% (6% - 10%)	12% (10% - 15%)
2018	CAJAMARCA	JAEN	3716	3231 (3215 - 3246)	6% (5% - 6%)	8% (7% - 9%)
2018	CAJAMARCA	SAN IGNACIO	827	3206 (3177 - 3236)	4% (3% - 6%)	7% (6% - 10%)
2018	CAJAMARCA	SAN MARCOS	350	3070 (3025 - 3115)	6% (4% - 9%)	10% (7% - 14%)
2018	CAJAMARCA	SAN MIGUEL	158	3039 (2975 - 3104)	9% (5% - 14%)	15% (9% - 21%)
2018	CAJAMARCA	SAN PABLO	109	3033 (2946 - 3121)	13% (7% - 21%)	15% (9% - 23%)
2018	CAJAMARCA	SANTA CRUZ	172	3203 (3132 - 3274)	5% (2% - 10%)	7% (4% - 12%)
2019	CAJAMARCA	CAJABAMBA	1105	3178 (3151 - 3204)	4% (3% - 6%)	8% (7% - 10%)
2019	CAJAMARCA	CAJAMARCA	7414	3047 (3035 - 3059)	12% (11% - 12%)	10% (9% - 11%)
2019	CAJAMARCA	CELENDIN	610	3129 (3093 - 3166)	7% (5% - 9%)	11% (9% - 14%)
2019	CAJAMARCA	CHOTA	1813	3094 (3073 - 3115)	8% (7% - 9%)	10% (8% - 11%)
2019	CAJAMARCA	CONTUMAZA	127	3231 (3166 - 3296)	2% (0% - 6%)	6% (3% - 12%)
2019	CAJAMARCA	CUTERVO	1281	3164 (3139 - 3189)	7% (5% - 8%)	9% (7% - 10%)
2019	CAJAMARCA	HUALGAYOC	704	3072 (3040 - 3104)	7% (5% - 9%)	10% (8% - 12%)
2019	CAJAMARCA	JAEN	3596	3246 (3230 - 3261)	5% (4% - 6%)	7% (6% - 8%)
2019	CAJAMARCA	SAN IGNACIO	739	3186 (3156 - 3216)	3% (2% - 5%)	9% (7% - 11%)
2019	CAJAMARCA	SAN MARCOS	346	3083 (3038 - 3128)	6% (4% - 9%)	11% (8% - 15%)
2019	CAJAMARCA	SAN MIGUEL	148	3000 (2922 - 3078)	16% (10% - 22%)	20% (14% - 28%)
2019	CAJAMARCA	SAN PABLO	116	3082 (3011 - 3154)	7% (3% - 13%)	11% (6% - 18%)
2019	CAJAMARCA	SANTA CRUZ	155	3244 (3185 - 3303)	3% (1% - 7%)	7% (4% - 12%)
2012	CALLAO	CALLAO	6950	3326 (3314 - 3338)	5% (4% - 5%)	5% (4% - 5%)
2013	CALLAO	CALLAO	10245	3329 (3319 - 3340)	6% (5% - 6%)	4% (4% - 4%)
2014	CALLAO	CALLAO	11601	3328 (3318 - 3338)	6% (6% - 7%)	4% (4% - 5%)
2015	CALLAO	CALLAO	16867	3293 (3284 - 3301)	8% (7% - 8%)	4% (4% - 5%)

2016	CALLAO	CALLAO	16321	3290 (3281 - 3299)	7% (7% - 8%)	4% (4% - 4%)
2017	CALLAO	CALLAO	18534	3315 (3307 - 3323)	7% (6% - 7%)	4% (3% - 4%)
2018	CALLAO	CALLAO	20009	3327 (3319 - 3335)	7% (6% - 7%)	4% (3% - 4%)
2019	CALLAO	CALLAO	19263	3318 (3310 - 3326)	7% (6% - 7%)	3% (3% - 4%)
2012	CUSCO	CANCHIS	625	3162 (3124 - 3200)	7% (5% - 9%)	5% (4% - 7%)
2012	CUSCO	CUSCO	3858	3128 (3111 - 3145)	10% (9% - 11%)	10% (9% - 11%)
2013	CUSCO	ANTA	79	3139 (3065 - 3213)	1% (0% - 7%)	6% (2% - 14%)
2013	CUSCO	CALCA	180	3207 (3155 - 3259)	1% (0% - 4%)	8% (4% - 13%)
2013	CUSCO	CANCHIS	1353	3153 (3127 - 3178)	7% (6% - 9%)	10% (9% - 12%)
2013	CUSCO	CUSCO	9616	3151 (3140 - 3161)	9% (8% - 9%)	8% (8% - 9%)
2013	CUSCO	ESPINAR	537	3112 (3075 - 3149)	6% (4% - 9%)	6% (4% - 8%)
2013	CUSCO	LA CONVENCION	953	3371 (3338 - 3403)	3% (2% - 5%)	5% (4% - 7%)
2013	CUSCO	URUBAMBA	100	3175 (3101 - 3249)	3% (1% - 9%)	3% (1% - 9%)
2014	CUSCO	ANTA	360	3131 (3094 - 3168)	1% (0% - 3%)	6% (4% - 9%)
2014	CUSCO	CALCA	623	3212 (3181 - 3242)	2% (1% - 4%)	6% (5% - 9%)
2014	CUSCO	CANCHIS	1467	3155 (3132 - 3178)	6% (5% - 8%)	7% (6% - 8%)
2014	CUSCO	CUSCO	12211	3166 (3157 - 3175)	8% (7% - 8%)	8% (7% - 8%)
2014	CUSCO	ESPINAR	692	3056 (3023 - 3088)	9% (7% - 11%)	12% (10% - 14%)
2014	CUSCO	LA CONVENCION	1439	3360 (3334 - 3386)	4% (3% - 5%)	3% (2% - 4%)
2014	CUSCO	URUBAMBA	414	3198 (3161 - 3235)	3% (2% - 5%)	4% (2% - 6%)
2015	CUSCO	ANTA	328	3127 (3084 - 3170)	5% (3% - 7%)	8% (5% - 11%)
2015	CUSCO	CALCA	562	3188 (3152 - 3224)	6% (4% - 8%)	8% (6% - 11%)
2015	CUSCO	CANAS	120	3089 (3017 - 3161)	5% (2% - 11%)	2% (1% - 7%)
2015	CUSCO	CANCHIS	1905	3199 (3180 - 3219)	5% (4% - 6%)	7% (6% - 8%)
2015	CUSCO	CHUMBIVILCAS	82	3074 (2967 - 3182)	10% (4% - 18%)	9% (4% - 17%)
2015	CUSCO	CUSCO	12924	3172 (3163 - 3181)	8% (7% - 8%)	8% (7% - 8%)
2015	CUSCO	ESPINAR	731	3129 (3099 - 3160)	6% (4% - 8%)	8% (6% - 11%)
2015	CUSCO	LA CONVENCION	2335	3325 (3305 - 3345)	4% (3% - 5%)	4% (3% - 5%)
2015	CUSCO	QUISPICANCHI	457	3152 (3113 - 3191)	6% (4% - 8%)	9% (6% - 11%)
2015	CUSCO	URUBAMBA	382	3157 (3112 - 3202)	7% (4% - 10%)	9% (7% - 13%)

2016	CUSCO	ANTA	276	3104 (3059 - 3149)	6% (3% - 9%)	8% (5% - 11%)
2016	CUSCO	CALCA	562	3201 (3168 - 3233)	3% (2% - 5%)	5% (3% - 7%)
2016	CUSCO	CANAS	144	3038 (2979 - 3096)	7% (3% - 12%)	10% (6% - 17%)
2016	CUSCO	CANCHIS	1808	3213 (3193 - 3234)	5% (4% - 6%)	5% (4% - 6%)
2016	CUSCO	CHUMBIVILCAS	619	3085 (3051 - 3120)	8% (6% - 10%)	9% (7% - 12%)
2016	CUSCO	CUSCO	12799	3178 (3169 - 3187)	7% (7% - 8%)	6% (6% - 7%)
2016	CUSCO	ESPINAR	975	3150 (3125 - 3175)	4% (3% - 6%)	6% (5% - 8%)
2016	CUSCO	LA CONVENCION	2207	3329 (3309 - 3350)	4% (3% - 5%)	3% (3% - 4%)
2016	CUSCO	PARURO	30	3188 (3057 - 3319)		3% (0% - 17%)
2016	CUSCO	PAUCARTAMBO	256	3126 (3078 - 3175)	3% (1% - 6%)	8% (5% - 12%)
2016	CUSCO	QUISPICANCHI	568	3179 (3145 - 3212)	4% (2% - 6%)	6% (4% - 8%)
2016	CUSCO	URUBAMBA	398	3228 (3187 - 3268)	4% (2% - 6%)	4% (2% - 6%)
2017	CUSCO	ANTA	268	3147 (3103 - 3192)	5% (3% - 8%)	6% (4% - 10%)
2017	CUSCO	CALCA	511	3185 (3152 - 3218)	3% (2% - 5%)	5% (3% - 7%)
2017	CUSCO	CANAS	268	3129 (3081 - 3177)	5% (3% - 8%)	5% (3% - 8%)
2017	CUSCO	CANCHIS	1949	3211 (3191 - 3231)	5% (4% - 6%)	5% (4% - 7%)
2017	CUSCO	CHUMBIVILCAS	642	3142 (3110 - 3174)	4% (3% - 6%)	8% (6% - 10%)
2017	CUSCO	CUSCO	13098	3181 (3173 - 3190)	8% (7% - 8%)	6% (6% - 7%)
2017	CUSCO	ESPINAR	1017	3178 (3153 - 3202)	4% (3% - 5%)	5% (4% - 7%)
2017	CUSCO	LA CONVENCION	2382	3347 (3327 - 3366)	4% (3% - 4%)	5% (4% - 6%)
2017	CUSCO	PARURO	69	3110 (3011 - 3209)	3% (0% - 10%)	
2017	CUSCO	PAUCARTAMBO	270	3140 (3093 - 3187)	5% (3% - 9%)	9% (5% - 13%)
2017	CUSCO	QUISPICANCHI	593	3144 (3111 - 3178)	6% (5% - 9%)	9% (7% - 12%)
2017	CUSCO	URUBAMBA	394	3246 (3204 - 3288)	3% (2% - 6%)	4% (2% - 6%)
2018	CUSCO	ACOMAYO	99	3171 (3094 - 3247)	4% (1% - 10%)	7% (3% - 14%)
2018	CUSCO	ANTA	268	3176 (3135 - 3217)	1% (0% - 3%)	4% (2% - 7%)
2018	CUSCO	CALCA	526	3213 (3181 - 3245)	3% (2% - 5%)	5% (3% - 7%)
2018	CUSCO	CANAS	216	3130 (3076 - 3184)	4% (2% - 7%)	7% (4% - 12%)
2018	CUSCO	CANCHIS	1844	3230 (3210 - 3250)	4% (3% - 5%)	6% (5% - 8%)
2018	CUSCO	CHUMBIVILCAS	854	3122 (3095 - 3149)	5% (4% - 7%)	7% (6% - 9%)

2018	CUSCO	CUSCO	12630	3190 (3181 - 3199)	8% (7% - 8%)	6% (6% - 7%)
2018	CUSCO	ESPINAR	983	3133 (3107 - 3159)	5% (3% - 6%)	7% (5% - 9%)
2018	CUSCO	LA CONVENCION	2341	3306 (3286 - 3326)	4% (3% - 5%)	4% (3% - 5%)
2018	CUSCO	PARURO	36	3209 (3105 - 3313)		
2018	CUSCO	PAUCARTAMBO	240	3105 (3054 - 3156)	5% (2% - 8%)	7% (4% - 11%)
2018	CUSCO	QUISPICANCHI	777	3172 (3145 - 3199)	5% (3% - 6%)	6% (5% - 8%)
2018	CUSCO	URUBAMBA	363	3250 (3208 - 3292)	3% (2% - 6%)	5% (3% - 7%)
2019	CUSCO	ACOMAYO	90	3141 (3056 - 3227)	7% (2% - 14%)	4% (1% - 11%)
2019	CUSCO	ANTA	223	3167 (3121 - 3213)	2% (0% - 5%)	4% (2% - 7%)
2019	CUSCO	CALCA	521	3198 (3166 - 3230)	4% (2% - 6%)	6% (4% - 9%)
2019	CUSCO	CANAS	206	3106 (3057 - 3155)	2% (1% - 5%)	6% (3% - 10%)
2019	CUSCO	CANCHIS	1832	3234 (3214 - 3253)	4% (3% - 5%)	6% (5% - 7%)
2019	CUSCO	CHUMBIVILCAS	838	3152 (3126 - 3178)	4% (3% - 5%)	7% (5% - 9%)
2019	CUSCO	CUSCO	12373	3197 (3188 - 3206)	8% (7% - 8%)	6% (6% - 7%)
2019	CUSCO	ESPINAR	920	3173 (3146 - 3201)	5% (3% - 6%)	5% (4% - 7%)
2019	CUSCO	LA CONVENCION	2590	3313 (3294 - 3331)	4% (4% - 5%)	5% (4% - 6%)
2019	CUSCO	PARURO	39	3181 (3061 - 3301)	3% (0% - 13%)	3% (0% - 13%)
2019	CUSCO	PAUCARTAMBO	211	3165 (3114 - 3217)	3% (1% - 6%)	6% (3% - 10%)
2019	CUSCO	QUISPICANCHI	733	3188 (3159 - 3216)	4% (3% - 5%)	5% (3% - 7%)
2019	CUSCO	URUBAMBA	395	3232 (3192 - 3271)	2% (1% - 4%)	4% (2% - 6%)
2013	HUANCAVELICA	ACOBAMBA	473	3019 (2984 - 3054)	7% (5% - 10%)	11% (9% - 14%)
2013	HUANCAVELICA	ANGARAES	331	3049 (3006 - 3092)	5% (3% - 8%)	11% (8% - 15%)
2013	HUANCAVELICA	HUANCAVELICA	1137	2991 (2963 - 3019)	12% (10% - 14%)	15% (13% - 17%)
2013	HUANCAVELICA	TAYACAJA	665	3064 (3029 - 3099)	9% (7% - 12%)	12% (10% - 15%)
2014	HUANCAVELICA	ACOBAMBA	691	3019 (2988 - 3049)	8% (6% - 11%)	13% (10% - 15%)
2014	HUANCAVELICA	ANGARAES	533	3060 (3028 - 3092)	6% (4% - 8%)	12% (9% - 15%)
2014	HUANCAVELICA	CHURCAMPA	108	3070 (2975 - 3165)	9% (5% - 16%)	21% (14% - 30%)
2014	HUANCAVELICA	HUANCAVELICA	1967	2991 (2970 - 3012)	12% (11% - 14%)	14% (12% - 16%)
2014	HUANCAVELICA	TAYACAJA	963	3066 (3037 - 3096)	10% (8% - 12%)	12% (10% - 14%)
2015	HUANCAVELICA	ACOBAMBA	617	3015 (2986 - 3044)	8% (6% - 11%)	12% (9% - 14%)

2015	HUANCAVELICA	ANGARAES	649	3063 (3033 - 3093)	8% (6% - 10%)	12% (9% - 14%)
2015	HUANCAVELICA	CHURCAMPA	431	3068 (3033 - 3104)	5% (3% - 7%)	10% (7% - 13%)
2015	HUANCAVELICA	HUANCAVELICA	2337	3033 (3014 - 3052)	11% (9% - 12%)	12% (11% - 13%)
2015	HUANCAVELICA	HUAYTARA	69	3119 (3031 - 3208)	4% (1% - 12%)	9% (3% - 18%)
2015	HUANCAVELICA	TAYACAJA	909	3093 (3067 - 3120)	6% (4% - 7%)	11% (9% - 13%)
2016	HUANCAVELICA	ACOBAMBA	648	3043 (3013 - 3073)	7% (5% - 9%)	9% (7% - 11%)
2016	HUANCAVELICA	ANGARAES	703	3030 (3003 - 3058)	7% (5% - 9%)	12% (10% - 15%)
2016	HUANCAVELICA	CASTROVIRREYNA	74	3019 (2926 - 3112)	9% (4% - 19%)	8% (3% - 17%)
2016	HUANCAVELICA	CHURCAMPA	405	3090 (3054 - 3127)	4% (2% - 6%)	8% (5% - 11%)
2016	HUANCAVELICA	HUANCAVELICA	2229	3012 (2993 - 3031)	11% (9% - 12%)	13% (11% - 14%)
2016	HUANCAVELICA	HUAYTARA	106	3115 (3034 - 3195)	5% (2% - 11%)	8% (4% - 16%)
2016	HUANCAVELICA	TAYACAJA	880	3108 (3081 - 3135)	6% (5% - 8%)	9% (7% - 11%)
2017	HUANCAVELICA	ACOBAMBA	703	2987 (2957 - 3017)	10% (8% - 13%)	14% (12% - 17%)
2017	HUANCAVELICA	ANGARAES	718	3096 (3066 - 3125)	5% (4% - 7%)	11% (9% - 14%)
2017	HUANCAVELICA	CASTROVIRREYNA	78	2903 (2831 - 2976)	5% (1% - 13%)	18% (10% - 28%)
2017	HUANCAVELICA	CHURCAMPA	415	3081 (3043 - 3118)	5% (3% - 7%)	11% (8% - 14%)
2017	HUANCAVELICA	HUANCAVELICA	2349	3029 (3010 - 3049)	10% (9% - 12%)	12% (10% - 13%)
2017	HUANCAVELICA	HUAYTARA	73	3086 (2995 - 3177)	7% (2% - 15%)	11% (5% - 20%)
2017	HUANCAVELICA	TAYACAJA	1012	3074 (3047 - 3101)	8% (6% - 9%)	9% (7% - 11%)
2018	HUANCAVELICA	ACOBAMBA	572	3066 (3031 - 3100)	7% (5% - 9%)	12% (10% - 15%)
2018	HUANCAVELICA	ANGARAES	705	3074 (3046 - 3101)	5% (3% - 6%)	11% (9% - 14%)
2018	HUANCAVELICA	CASTROVIRREYNA	67	3103 (3011 - 3195)	1% (0% - 8%)	10% (4% - 20%)
2018	HUANCAVELICA	CHURCAMPA	423	3122 (3085 - 3159)	5% (3% - 8%)	11% (8% - 15%)
2018	HUANCAVELICA	HUANCAVELICA	2273	3030 (3010 - 3049)	11% (9% - 12%)	13% (11% - 14%)
2018	HUANCAVELICA	HUAYTARA	81	3000 (2901 - 3098)	9% (4% - 17%)	16% (9% - 26%)
2018	HUANCAVELICA	TAYACAJA	1066	3089 (3065 - 3113)	6% (5% - 8%)	8% (6% - 9%)
2019	HUANCAVELICA	ACOBAMBA	597	3081 (3048 - 3115)	7% (5% - 9%)	14% (11% - 17%)
2019	HUANCAVELICA	ANGARAES	703	3094 (3068 - 3121)	4% (3% - 6%)	9% (7% - 11%)
2019	HUANCAVELICA	CASTROVIRREYNA	67	3069 (2961 - 3178)	9% (3% - 18%)	9% (3% - 18%)
2019	HUANCAVELICA	CHURCAMPA	383	3097 (3059 - 3134)	5% (3% - 8%)	9% (6% - 12%)

2019	HUANCAVELICA	HUANCAVELICA	2188	3038 (3019 - 3057)	10% (9% - 12%)	12% (11% - 13%)
2019	HUANCAVELICA	HUAYTARA	72	3057 (2976 - 3138)	4% (1% - 12%)	10% (4% - 19%)
2019	HUANCAVELICA	TAYACAJA	1042	3123 (3096 - 3149)	5% (4% - 7%)	6% (5% - 8%)
2012	HUANUCO	HUANUCO	910	3137 (3100 - 3173)	11% (9% - 14%)	10% (8% - 12%)
2012	HUANUCO	LEONCIO PRADO	614	3292 (3253 - 3331)	5% (3% - 7%)	7% (5% - 9%)
2013	HUANUCO	HUANUCO	3543	3134 (3116 - 3153)	10% (9% - 12%)	9% (8% - 10%)
2013	HUANUCO	LEONCIO PRADO	2456	3307 (3287 - 3327)	5% (4% - 6%)	6% (5% - 7%)
2013	HUANUCO	PACHITEA	223	3027 (2974 - 3079)	6% (3% - 10%)	12% (8% - 17%)
2014	HUANUCO	AMBO	317	3156 (3115 - 3198)	5% (3% - 8%)	8% (5% - 12%)
2014	HUANUCO	DOS DE MAYO	211	3068 (3001 - 3135)	8% (5% - 13%)	11% (7% - 16%)
2014	HUANUCO	HUACAYBAMBA	46	3048 (2925 - 3171)	9% (2% - 21%)	9% (2% - 21%)
2014	HUANUCO	HUAMALIES	268	3100 (3041 - 3159)	7% (4% - 10%)	14% (10% - 19%)
2014	HUANUCO	HUANUCO	5306	3203 (3189 - 3217)	8% (7% - 8%)	7% (7% - 8%)
2014	HUANUCO	LEONCIO PRADO	2685	3336 (3318 - 3354)	4% (3% - 5%)	4% (3% - 5%)
2014	HUANUCO	PACHITEA	519	2992 (2957 - 3026)	7% (5% - 10%)	13% (10% - 16%)
2014	HUANUCO	PUERTO INCA	52	3243 (3114 - 3372)	6% (1% - 16%)	10% (3% - 21%)
2014	HUANUCO	YAROWILCA	39	2995 (2871 - 3119)	13% (4% - 27%)	13% (4% - 27%)
2015	HUANUCO	AMBO	452	3149 (3114 - 3184)	4% (3% - 6%)	7% (5% - 10%)
2015	HUANUCO	DOS DE MAYO	373	3050 (3008 - 3093)	8% (6% - 12%)	9% (6% - 13%)
2015	HUANUCO	HUACAYBAMBA	104	3078 (2996 - 3159)	6% (2% - 12%)	7% (3% - 13%)
2015	HUANUCO	HUAMALIES	632	3039 (3005 - 3073)	8% (6% - 10%)	16% (13% - 19%)
2015	HUANUCO	HUANUCO	6187	3178 (3166 - 3191)	8% (7% - 9%)	7% (7% - 8%)
2015	HUANUCO	LAURICOCHA	75	2969 (2880 - 3059)	8% (3% - 17%)	12% (6% - 22%)
2015	HUANUCO	LEONCIO PRADO	3175	3320 (3304 - 3337)	4% (3% - 5%)	5% (4% - 5%)
2015	HUANUCO	MARAÑON	69	3024 (2922 - 3126)	7% (2% - 16%)	13% (6% - 23%)
2015	HUANUCO	PACHITEA	857	3052 (3023 - 3080)	7% (6% - 9%)	11% (9% - 14%)
2015	HUANUCO	PUERTO INCA	195	3175 (3115 - 3235)	5% (2% - 9%)	9% (5% - 14%)
2015	HUANUCO	YAROWILCA	202	3032 (2978 - 3086)	4% (2% - 8%)	13% (9% - 19%)
2016	HUANUCO	AMBO	602	3156 (3122 - 3189)	4% (3% - 6%)	7% (5% - 10%)
2016	HUANUCO	DOS DE MAYO	436	3022 (2984 - 3059)	8% (5% - 11%)	12% (9% - 15%)

2016	HUANUCO	HUACAYBAMBA	125	3108 (3033 - 3182)	8% (4% - 14%)	6% (2% - 11%)
2016	HUANUCO	HUAMALIES	679	3008 (2973 - 3043)	11% (8% - 13%)	14% (11% - 17%)
2016	HUANUCO	HUANUCO	6418	3193 (3182 - 3205)	7% (6% - 7%)	6% (6% - 7%)
2016	HUANUCO	LAURICOCHA	197	2979 (2917 - 3041)	9% (6% - 14%)	13% (9% - 19%)
2016	HUANUCO	LEONCIO PRADO	3049	3334 (3317 - 3351)	4% (3% - 5%)	4% (3% - 5%)
2016	HUANUCO	MARAÑON	83	3036 (2950 - 3122)	6% (2% - 14%)	17% (10% - 27%)
2016	HUANUCO	PACHITEA	853	3056 (3026 - 3085)	8% (6% - 10%)	14% (12% - 17%)
2016	HUANUCO	PUERTO INCA	329	3258 (3208 - 3308)	4% (2% - 7%)	10% (7% - 14%)
2016	HUANUCO	YAROWILCA	284	3020 (2977 - 3062)	7% (5% - 11%)	10% (7% - 14%)
2017	HUANUCO	AMBO	624	3146 (3115 - 3177)	4% (2% - 5%)	7% (5% - 9%)
2017	HUANUCO	DOS DE MAYO	519	3087 (3050 - 3125)	7% (5% - 9%)	7% (5% - 10%)
2017	HUANUCO	HUACAYBAMBA	138	3080 (3003 - 3157)	9% (5% - 15%)	5% (2% - 10%)
2017	HUANUCO	HUAMALIES	722	3017 (2988 - 3047)	7% (5% - 9%)	14% (12% - 17%)
2017	HUANUCO	HUANUCO	6855	3183 (3171 - 3195)	7% (6% - 8%)	6% (6% - 7%)
2017	HUANUCO	LAURICOCHA	209	2966 (2915 - 3018)	8% (5% - 13%)	18% (13% - 24%)
2017	HUANUCO	LEONCIO PRADO	3379	3339 (3323 - 3355)	4% (3% - 5%)	5% (4% - 6%)
2017	HUANUCO	PACHITEA	988	3113 (3087 - 3139)	6% (4% - 8%)	8% (6% - 9%)
2017	HUANUCO	PUERTO INCA	416	3237 (3195 - 3279)	4% (3% - 7%)	6% (4% - 9%)
2017	HUANUCO	YAROWILCA	252	2998 (2949 - 3047)	8% (5% - 12%)	15% (11% - 21%)
2018	HUANUCO	AMBO	651	3166 (3137 - 3195)	3% (2% - 5%)	4% (2% - 5%)
2018	HUANUCO	DOS DE MAYO	466	3063 (3021 - 3104)	8% (6% - 11%)	9% (7% - 12%)
2018	HUANUCO	HUACAYBAMBA	181	3043 (2984 - 3102)	7% (4% - 12%)	10% (6% - 15%)
2018	HUANUCO	HUAMALIES	698	3032 (3000 - 3063)	7% (5% - 9%)	13% (10% - 16%)
2018	HUANUCO	HUANUCO	6841	3170 (3158 - 3182)	8% (8% - 9%)	6% (5% - 7%)
2018	HUANUCO	LAURICOCHA	203	3034 (2972 - 3096)	10% (6% - 15%)	14% (10% - 20%)
2018	HUANUCO	LEONCIO PRADO	3190	3337 (3321 - 3354)	4% (3% - 5%)	4% (3% - 5%)
2018	HUANUCO	MARAÑON	100	3093 (3013 - 3172)	5% (2% - 11%)	7% (3% - 14%)
2018	HUANUCO	PACHITEA	795	3186 (3156 - 3216)	4% (3% - 6%)	5% (4% - 7%)
2018	HUANUCO	PUERTO INCA	421	3267 (3223 - 3311)	4% (2% - 6%)	9% (6% - 12%)
2018	HUANUCO	YAROWILCA	266	3002 (2951 - 3052)	11% (7% - 15%)	15% (11% - 19%)

2019	HUANUCO	AMBO	615	3159 (3128 - 3190)	3% (2% - 5%)	5% (4% - 7%)
2019	HUANUCO	DOS DE MAYO	506	3024 (2986 - 3061)	8% (6% - 11%)	12% (10% - 16%)
2019	HUANUCO	HUACAYBAMBA	221	3106 (3047 - 3164)	8% (5% - 12%)	9% (6% - 14%)
2019	HUANUCO	HUAMALIES	670	3023 (2988 - 3058)	10% (8% - 13%)	14% (12% - 17%)
2019	HUANUCO	HUANUCO	6763	3204 (3192 - 3216)	7% (7% - 8%)	5% (5% - 6%)
2019	HUANUCO	LAURICOCHA	175	2954 (2893 - 3015)	8% (4% - 13%)	13% (9% - 19%)
2019	HUANUCO	LEONCIO PRADO	3021	3339 (3322 - 3357)	4% (3% - 5%)	4% (3% - 4%)
2019	HUANUCO	MARAÑON	207	2964 (2902 - 3026)	13% (8% - 18%)	21% (15% - 27%)
2019	HUANUCO	PACHITEA	892	3198 (3170 - 3226)	3% (2% - 5%)	5% (4% - 7%)
2019	HUANUCO	PUERTO INCA	441	3207 (3165 - 3249)	5% (3% - 7%)	8% (6% - 11%)
2019	HUANUCO	YAROWILCA	256	3046 (2999 - 3094)	8% (5% - 12%)	11% (7% - 15%)
2012	ICA	CHINCHA	129	3320 (3229 - 3412)	7% (3% - 13%)	5% (2% - 11%)
2012	ICA	ICA	212	3349 (3283 - 3415)	4% (2% - 8%)	8% (4% - 12%)
2013	ICA	CHINCHA	2349	3291 (3270 - 3311)	6% (5% - 7%)	6% (5% - 7%)
2013	ICA	ICA	2745	3343 (3324 - 3361)	4% (3% - 5%)	5% (4% - 5%)
2014	ICA	CHINCHA	2891	3324 (3306 - 3341)	4% (4% - 5%)	5% (5% - 6%)
2014	ICA	ICA	4001	3361 (3345 - 3376)	4% (4% - 5%)	4% (3% - 5%)
2014	ICA	PISCO	728	3382 (3349 - 3416)	3% (2% - 4%)	4% (3% - 6%)
2015	ICA	CHINCHA	3896	3320 (3305 - 3336)	4% (3% - 5%)	4% (4% - 5%)
2015	ICA	ICA	8156	3351 (3339 - 3362)	5% (5% - 6%)	4% (4% - 5%)
2015	ICA	NAZCA	420	3358 (3311 - 3404)	3% (1% - 5%)	4% (3% - 7%)
2015	ICA	PISCO	2873	3371 (3353 - 3388)	3% (2% - 4%)	5% (4% - 6%)
2016	ICA	CHINCHA	3883	3308 (3293 - 3324)	4% (4% - 5%)	4% (4% - 5%)
2016	ICA	ICA	8074	3370 (3358 - 3381)	5% (4% - 5%)	4% (3% - 4%)
2016	ICA	NAZCA	945	3356 (3324 - 3388)	4% (3% - 6%)	5% (3% - 6%)
2016	ICA	PALPA	122	3368 (3289 - 3447)	2% (1% - 7%)	2% (1% - 7%)
2016	ICA	PISCO	2923	3394 (3377 - 3412)	3% (2% - 4%)	5% (4% - 6%)
2017	ICA	CHINCHA	3922	3307 (3291 - 3322)	4% (4% - 5%)	4% (4% - 5%)
2017	ICA	ICA	7839	3367 (3356 - 3379)	5% (5% - 6%)	4% (3% - 4%)
2017	ICA	NAZCA	1178	3395 (3367 - 3423)	3% (2% - 4%)	3% (2% - 4%)

2017	ICA	PALPA	76	3416 (3337 - 3496)	1% (0% - 7%)	1% (0% - 7%)
2017	ICA	PISCO	2993	3381 (3363 - 3398)	3% (3% - 4%)	5% (4% - 5%)
2018	ICA	CHINCHA	4173	3323 (3309 - 3338)	4% (3% - 5%)	4% (3% - 4%)
2018	ICA	ICA	8095	3367 (3356 - 3378)	5% (4% - 5%)	3% (3% - 4%)
2018	ICA	NAZCA	1132	3387 (3359 - 3414)	3% (2% - 4%)	3% (2% - 4%)
2018	ICA	PALPA	65	3323 (3228 - 3418)		2% (0% - 8%)
2018	ICA	PISCO	2821	3403 (3386 - 3420)	2% (2% - 3%)	4% (3% - 5%)
2019	ICA	CHINCHA	4110	3338 (3323 - 3353)	4% (4% - 5%)	3% (3% - 4%)
2019	ICA	ICA	7803	3364 (3353 - 3376)	4% (4% - 5%)	3% (3% - 4%)
2019	ICA	NAZCA	1124	3377 (3351 - 3403)	2% (1% - 3%)	3% (2% - 4%)
2019	ICA	PALPA	59	3403 (3300 - 3507)		2% (0% - 9%)
2019	ICA	PISCO	2916	3358 (3340 - 3376)	4% (3% - 4%)	5% (5% - 6%)
2013	JUNIN	CHANCHAMAYO	799	3314 (3281 - 3346)	5% (3% - 6%)	9% (7% - 11%)
2013	JUNIN	HUANCAYO	1555	3045 (3019 - 3071)	12% (10% - 13%)	15% (13% - 17%)
2013	JUNIN	JAUJA	399	3058 (3012 - 3104)	11% (8% - 14%)	11% (8% - 14%)
2014	JUNIN	CHANCHAMAYO	2096	3316 (3295 - 3337)	5% (4% - 6%)	7% (6% - 8%)
2014	JUNIN	HUANCAYO	5455	3037 (3022 - 3051)	12% (11% - 13%)	17% (16% - 18%)
2014	JUNIN	JAUJA	1092	3112 (3087 - 3137)	6% (5% - 8%)	9% (8% - 11%)
2015	JUNIN	CHANCHAMAYO	2287	3307 (3288 - 3327)	5% (4% - 6%)	8% (6% - 9%)
2015	JUNIN	CHUPACA	261	3100 (3054 - 3146)	5% (3% - 9%)	10% (7% - 15%)
2015	JUNIN	CONCEPCION	152	3170 (3110 - 3230)	4% (1% - 8%)	5% (2% - 9%)
2015	JUNIN	HUANCAYO	7157	3052 (3040 - 3064)	11% (10% - 12%)	14% (13% - 14%)
2015	JUNIN	JAUJA	1042	3097 (3069 - 3126)	8% (6% - 10%)	8% (7% - 10%)
2015	JUNIN	JUNIN	194	2995 (2934 - 3056)	9% (6% - 14%)	12% (8% - 17%)
2015	JUNIN	SATIPO	903	3247 (3214 - 3280)	6% (5% - 8%)	7% (6% - 9%)
2015	JUNIN	TARMA	648	3116 (3079 - 3154)	8% (6% - 11%)	8% (6% - 11%)
2015	JUNIN	YAULI	174	3028 (2972 - 3083)	6% (3% - 10%)	7% (4% - 12%)
2016	JUNIN	CHANCHAMAYO	2735	3323 (3305 - 3341)	4% (3% - 5%)	5% (4% - 6%)
2016	JUNIN	CHUPACA	498	3114 (3078 - 3150)	7% (5% - 10%)	11% (8% - 14%)
2016	JUNIN	CONCEPCION	356	3149 (3111 - 3187)	3% (1% - 5%)	5% (3% - 8%)

2016	JUNIN	HUANCAYO	9971	3084 (3074 - 3094)	10% (9% - 10%)	12% (11% - 12%)
2016	JUNIN	JAUJA	1068	3115 (3091 - 3140)	6% (5% - 8%)	7% (6% - 9%)
2016	JUNIN	JUNIN	410	3017 (2973 - 3061)	9% (6% - 12%)	10% (7% - 13%)
2016	JUNIN	SATIPO	2373	3288 (3268 - 3308)	5% (4% - 6%)	5% (4% - 6%)
2016	JUNIN	TARMA	1445	3126 (3103 - 3149)	7% (6% - 8%)	6% (4% - 7%)
2016	JUNIN	YAULI	407	3108 (3067 - 3149)	5% (3% - 8%)	8% (5% - 11%)
2017	JUNIN	CHANCHAMAYO	2912	3315 (3298 - 3333)	4% (4% - 5%)	5% (5% - 6%)
2017	JUNIN	CHUPACA	471	3093 (3061 - 3125)	4% (2% - 6%)	9% (7% - 12%)
2017	JUNIN	CONCEPCION	334	3109 (3071 - 3148)	3% (1% - 5%)	8% (6% - 12%)
2017	JUNIN	HUANCAYO	10295	3111 (3102 - 3121)	9% (8% - 9%)	9% (9% - 10%)
2017	JUNIN	JAUJA	1058	3089 (3062 - 3116)	8% (7% - 10%)	8% (7% - 10%)
2017	JUNIN	JUNIN	353	3071 (3025 - 3116)	9% (7% - 13%)	10% (7% - 13%)
2017	JUNIN	SATIPO	2611	3259 (3240 - 3277)	6% (5% - 7%)	6% (6% - 7%)
2017	JUNIN	TARMA	1398	3167 (3143 - 3191)	7% (5% - 8%)	6% (5% - 7%)
2017	JUNIN	YAULI	373	3121 (3082 - 3161)	4% (2% - 7%)	8% (5% - 11%)
2018	JUNIN	CHANCHAMAYO	2894	3335 (3317 - 3352)	4% (3% - 4%)	5% (4% - 5%)
2018	JUNIN	CHUPACA	414	3099 (3061 - 3137)	5% (3% - 8%)	10% (8% - 14%)
2018	JUNIN	CONCEPCION	313	3089 (3049 - 3129)	6% (4% - 9%)	12% (9% - 17%)
2018	JUNIN	HUANCAYO	10263	3109 (3100 - 3119)	9% (8% - 9%)	10% (9% - 10%)
2018	JUNIN	JAUJA	975	3102 (3075 - 3130)	7% (6% - 9%)	7% (6% - 9%)
2018	JUNIN	JUNIN	329	3088 (3043 - 3134)	4% (2% - 7%)	11% (8% - 14%)
2018	JUNIN	SATIPO	2620	3249 (3230 - 3268)	6% (5% - 7%)	7% (6% - 8%)
2018	JUNIN	TARMA	1376	3182 (3157 - 3208)	6% (5% - 8%)	6% (5% - 8%)
2018	JUNIN	YAULI	402	3066 (3025 - 3107)	8% (5% - 11%)	7% (5% - 10%)
2019	JUNIN	CHANCHAMAYO	2841	3300 (3281 - 3318)	5% (4% - 6%)	6% (5% - 7%)
2019	JUNIN	CHUPACA	437	3131 (3098 - 3164)	3% (2% - 6%)	7% (4% - 9%)
2019	JUNIN	CONCEPCION	333	3151 (3109 - 3193)	3% (1% - 5%)	6% (4% - 9%)
2019	JUNIN	HUANCAYO	10922	3116 (3107 - 3126)	9% (8% - 9%)	9% (9% - 10%)
2019	JUNIN	JAUJA	957	3102 (3074 - 3130)	7% (6% - 9%)	8% (6% - 10%)
2019	JUNIN	JUNIN	277	3097 (3050 - 3145)	6% (4% - 10%)	7% (4% - 11%)

2019	JUNIN	SATIPO	2688	3232 (3213 - 3251)	6% (5% - 7%)	7% (7% - 9%)
2019	JUNIN	TARMA	1310	3196 (3171 - 3221)	6% (4% - 7%)	5% (4% - 7%)
2019	JUNIN	YAULI	374	3096 (3052 - 3140)	6% (4% - 9%)	7% (5% - 10%)
2012	LA LIBERTAD	TRUJILLO	523	3216 (3164 - 3269)	11% (8% - 14%)	5% (4% - 8%)
2013	LA LIBERTAD	ASCOPE	165	3413 (3343 - 3484)	1% (0% - 3%)	2% (0% - 5%)
2013	LA LIBERTAD	CHEPEN	411	3321 (3278 - 3364)	3% (2% - 6%)	4% (2% - 7%)
2013	LA LIBERTAD	GRAN CHIMU	46	3225 (3087 - 3363)	4% (1% - 15%)	9% (2% - 21%)
2013	LA LIBERTAD	OTUZCO	162	3083 (3015 - 3151)	6% (3% - 10%)	8% (4% - 13%)
2013	LA LIBERTAD	PACASMAYO	1065	3357 (3332 - 3382)	2% (1% - 3%)	3% (2% - 4%)
2013	LA LIBERTAD	SANCHEZ CARRION	103	3023 (2944 - 3102)	5% (2% - 11%)	11% (5% - 18%)
2013	LA LIBERTAD	TRUJILLO	8577	3243 (3230 - 3256)	10% (9% - 10%)	6% (5% - 6%)
2013	LA LIBERTAD	VIRU	258	3371 (3320 - 3422)	1% (0% - 3%)	2% (0% - 4%)
2014	LA LIBERTAD	ASCOPE	554	3409 (3372 - 3446)	2% (1% - 4%)	3% (2% - 4%)
2014	LA LIBERTAD	CHEPEN	1189	3312 (3285 - 3338)	4% (3% - 5%)	4% (3% - 5%)
2014	LA LIBERTAD	GRAN CHIMU	94	3160 (3068 - 3251)	5% (2% - 12%)	11% (5% - 19%)
2014	LA LIBERTAD	OTUZCO	342	3106 (3059 - 3153)	7% (4% - 10%)	12% (8% - 16%)
2014	LA LIBERTAD	PACASMAYO	1161	3368 (3341 - 3394)	3% (2% - 4%)	3% (2% - 5%)
2014	LA LIBERTAD	PATAZ	82	3118 (3010 - 3225)	6% (2% - 14%)	10% (4% - 18%)
2014	LA LIBERTAD	SANCHEZ CARRION	163	3055 (2992 - 3119)	9% (5% - 15%)	10% (6% - 16%)
2014	LA LIBERTAD	SANTIAGO DE CHUCO	131	2948 (2872 - 3024)	12% (7% - 19%)	18% (11% - 25%)
2014	LA LIBERTAD	TRUJILLO	10782	3285 (3274 - 3296)	8% (7% - 8%)	5% (5% - 6%)
2014	LA LIBERTAD	VIRU	473	3360 (3319 - 3401)	2% (1% - 4%)	4% (2% - 6%)
2015	LA LIBERTAD	ASCOPE	1270	3343 (3317 - 3368)	3% (2% - 4%)	4% (3% - 5%)
2015	LA LIBERTAD	CHEPEN	1183	3302 (3275 - 3330)	4% (3% - 5%)	4% (3% - 6%)
2015	LA LIBERTAD	GRAN CHIMU	70	3141 (3037 - 3245)	6% (2% - 14%)	13% (6% - 23%)
2015	LA LIBERTAD	JULCAN	122	2977 (2910 - 3043)	10% (5% - 17%)	16% (10% - 24%)
2015	LA LIBERTAD	OTUZCO	485	3016 (2975 - 3057)	9% (6% - 12%)	15% (12% - 19%)
2015	LA LIBERTAD	PACASMAYO	1156	3328 (3302 - 3354)	3% (2% - 4%)	4% (3% - 5%)
2015	LA LIBERTAD	PATAZ	184	3185 (3122 - 3248)	3% (1% - 6%)	8% (5% - 13%)
2015	LA LIBERTAD	SANCHEZ CARRION	1113	3066 (3039 - 3094)	8% (6% - 9%)	11% (10% - 13%)

2015	LA LIBERTAD	SANTIAGO DE CHUCO	595	3013 (2980 - 3045)	9% (6% - 11%)	11% (9% - 14%)
2015	LA LIBERTAD	TRUJILLO	15336	3273 (3264 - 3282)	8% (7% - 8%)	5% (4% - 5%)
2015	LA LIBERTAD	VIRU	542	3352 (3315 - 3389)	1% (1% - 3%)	3% (1% - 4%)
2016	LA LIBERTAD	ASCOPE	1247	3358 (3334 - 3381)	2% (1% - 3%)	2% (2% - 4%)
2016	LA LIBERTAD	BOLIVAR	45	3067 (2950 - 3184)	4% (1% - 15%)	11% (4% - 24%)
2016	LA LIBERTAD	CHEPEN	1310	3333 (3309 - 3358)	3% (2% - 4%)	3% (2% - 4%)
2016	LA LIBERTAD	GRAN CHIMU	113	3206 (3134 - 3278)	4% (1% - 9%)	7% (3% - 13%)
2016	LA LIBERTAD	JULCAN	222	3052 (3001 - 3103)	6% (3% - 10%)	13% (9% - 18%)
2016	LA LIBERTAD	OTUZCO	585	3084 (3049 - 3118)	7% (5% - 10%)	11% (9% - 14%)
2016	LA LIBERTAD	PACASMAYO	1154	3390 (3364 - 3416)	3% (2% - 4%)	3% (2% - 4%)
2016	LA LIBERTAD	PATAZ	482	3161 (3120 - 3201)	5% (3% - 7%)	10% (7% - 13%)
2016	LA LIBERTAD	SANCHEZ CARRION	1871	3059 (3038 - 3081)	9% (7% - 10%)	12% (10% - 13%)
2016	LA LIBERTAD	SANTIAGO DE CHUCO	490	3068 (3029 - 3107)	8% (6% - 11%)	8% (6% - 11%)
2016	LA LIBERTAD	TRUJILLO	15174	3285 (3276 - 3295)	7% (7% - 8%)	4% (4% - 5%)
2016	LA LIBERTAD	VIRU	929	3359 (3334 - 3385)	2% (1% - 3%)	2% (1% - 3%)
2017	LA LIBERTAD	ASCOPE	1160	3350 (3324 - 3377)	3% (2% - 5%)	3% (2% - 4%)
2017	LA LIBERTAD	BOLIVAR	35	3115 (3005 - 3226)	6% (1% - 19%)	11% (3% - 27%)
2017	LA LIBERTAD	CHEPEN	1211	3306 (3279 - 3333)	4% (3% - 5%)	3% (2% - 4%)
2017	LA LIBERTAD	GRAN CHIMU	94	3136 (3014 - 3258)	13% (7% - 21%)	17% (10% - 26%)
2017	LA LIBERTAD	JULCAN	246	3006 (2947 - 3065)	12% (8% - 17%)	14% (10% - 19%)
2017	LA LIBERTAD	OTUZCO	577	3053 (3019 - 3088)	7% (5% - 10%)	12% (10% - 15%)
2017	LA LIBERTAD	PACASMAYO	1061	3347 (3319 - 3375)	3% (2% - 4%)	4% (3% - 6%)
2017	LA LIBERTAD	PATAZ	685	3162 (3131 - 3193)	5% (3% - 7%)	7% (5% - 9%)
2017	LA LIBERTAD	SANCHEZ CARRION	2248	3070 (3051 - 3088)	8% (7% - 9%)	12% (11% - 14%)
2017	LA LIBERTAD	SANTIAGO DE CHUCO	555	3029 (2992 - 3066)	9% (6% - 11%)	11% (9% - 14%)
2017	LA LIBERTAD	TRUJILLO	15792	3291 (3282 - 3299)	7% (7% - 8%)	5% (4% - 5%)
2017	LA LIBERTAD	VIRU	924	3373 (3345 - 3401)	2% (1% - 3%)	2% (1% - 3%)
2018	LA LIBERTAD	ASCOPE	1241	3386 (3361 - 3410)	2% (1% - 3%)	2% (2% - 4%)
2018	LA LIBERTAD	BOLIVAR	68	3152 (3061 - 3244)	1% (0% - 8%)	4% (1% - 12%)
2018	LA LIBERTAD	CHEPEN	1258	3356 (3330 - 3382)	4% (3% - 5%)	3% (3% - 5%)

2018	LA LIBERTAD	GRAN CHIMU	138	3118 (3042 - 3195)	4% (1% - 8%)	8% (4% - 14%)
2018	LA LIBERTAD	JULCAN	238	3051 (2999 - 3103)	7% (4% - 11%)	14% (10% - 19%)
2018	LA LIBERTAD	OTUZCO	529	3077 (3044 - 3110)	6% (4% - 9%)	11% (9% - 14%)
2018	LA LIBERTAD	PACASMAYO	1068	3348 (3322 - 3374)	2% (1% - 3%)	3% (2% - 4%)
2018	LA LIBERTAD	PATAZ	677	3142 (3110 - 3175)	5% (4% - 7%)	9% (7% - 12%)
2018	LA LIBERTAD	SANCHEZ CARRION	2378	3076 (3059 - 3093)	7% (6% - 8%)	10% (9% - 12%)
2018	LA LIBERTAD	SANTIAGO DE CHUCO	528	3054 (3016 - 3091)	8% (6% - 11%)	11% (8% - 14%)
2018	LA LIBERTAD	TRUJILLO	16618	3296 (3288 - 3305)	7% (7% - 8%)	4% (4% - 5%)
2018	LA LIBERTAD	VIRU	997	3403 (3377 - 3429)	2% (1% - 3%)	2% (1% - 3%)
2019	LA LIBERTAD	ASCOPE	1079	3391 (3366 - 3416)	2% (1% - 3%)	3% (2% - 4%)
2019	LA LIBERTAD	BOLIVAR	94	3110 (3027 - 3193)	1% (0% - 6%)	6% (2% - 13%)
2019	LA LIBERTAD	CHEPEN	1125	3294 (3267 - 3322)	4% (3% - 5%)	4% (3% - 6%)
2019	LA LIBERTAD	GRAN CHIMU	132	3223 (3152 - 3294)	5% (2% - 11%)	5% (2% - 11%)
2019	LA LIBERTAD	JULCAN	231	3023 (2965 - 3080)	6% (3% - 10%)	13% (9% - 18%)
2019	LA LIBERTAD	OTUZCO	505	3090 (3052 - 3128)	8% (5% - 10%)	10% (8% - 14%)
2019	LA LIBERTAD	PACASMAYO	1036	3385 (3358 - 3411)	2% (1% - 3%)	2% (2% - 4%)
2019	LA LIBERTAD	PATAZ	677	3161 (3130 - 3192)	4% (3% - 6%)	7% (5% - 9%)
2019	LA LIBERTAD	SANCHEZ CARRION	2231	3052 (3033 - 3071)	8% (7% - 9%)	12% (10% - 13%)
2019	LA LIBERTAD	SANTIAGO DE CHUCO	559	3038 (3002 - 3074)	9% (7% - 11%)	13% (10% - 16%)
2019	LA LIBERTAD	TRUJILLO	17088	3288 (3279 - 3296)	7% (6% - 7%)	4% (4% - 5%)
2019	LA LIBERTAD	VIRU	1165	3374 (3349 - 3399)	2% (2% - 3%)	2% (1% - 3%)
2012	LAMBAYEQUE	CHICLAYO	265	3162 (3089 - 3235)	7% (4% - 11%)	5% (2% - 8%)
2012	LAMBAYEQUE	FERREÑAFE	64	3347 (3227 - 3466)	5% (1% - 13%)	3% (0% - 11%)
2012	LAMBAYEQUE	LAMBAYEQUE	135	3310 (3231 - 3389)	4% (1% - 8%)	4% (1% - 8%)
2013	LAMBAYEQUE	CHICLAYO	1673	3202 (3173 - 3231)	11% (9% - 12%)	8% (7% - 10%)
2013	LAMBAYEQUE	FERREÑAFE	631	3340 (3308 - 3373)	1% (1% - 3%)	4% (3% - 6%)
2013	LAMBAYEQUE	LAMBAYEQUE	1540	3256 (3229 - 3282)	6% (5% - 7%)	7% (6% - 8%)
2014	LAMBAYEQUE	CHICLAYO	3039	3243 (3224 - 3263)	7% (6% - 8%)	4% (4% - 5%)
2014	LAMBAYEQUE	FERREÑAFE	686	3338 (3305 - 3370)	2% (1% - 4%)	5% (3% - 7%)
2014	LAMBAYEQUE	LAMBAYEQUE	3203	3281 (3264 - 3298)	5% (4% - 6%)	5% (5% - 6%)

2015	LAMBAYEQUE	CHICLAYO	9393	3223 (3212 - 3234)	9% (8% - 9%)	5% (5% - 6%)
2015	LAMBAYEQUE	FERREÑAFE	740	3170 (3139 - 3201)	5% (3% - 7%)	10% (8% - 13%)
2015	LAMBAYEQUE	LAMBAYEQUE	3900	3292 (3278 - 3307)	5% (4% - 5%)	5% (5% - 6%)
2016	LAMBAYEQUE	CHICLAYO	13168	3233 (3223 - 3242)	8% (8% - 9%)	5% (5% - 6%)
2016	LAMBAYEQUE	FERREÑAFE	713	3130 (3097 - 3162)	5% (4% - 7%)	11% (9% - 14%)
2016	LAMBAYEQUE	LAMBAYEQUE	4166	3287 (3273 - 3300)	4% (4% - 5%)	5% (4% - 6%)
2017	LAMBAYEQUE	CHICLAYO	13689	3242 (3232 - 3251)	8% (8% - 9%)	5% (5% - 5%)
2017	LAMBAYEQUE	FERREÑAFE	799	3176 (3145 - 3206)	6% (4% - 7%)	10% (8% - 12%)
2017	LAMBAYEQUE	LAMBAYEQUE	4643	3321 (3308 - 3334)	3% (3% - 4%)	4% (3% - 5%)
2018	LAMBAYEQUE	CHICLAYO	13742	3256 (3246 - 3266)	8% (8% - 9%)	4% (4% - 5%)
2018	LAMBAYEQUE	FERREÑAFE	851	3251 (3223 - 3280)	3% (2% - 5%)	7% (5% - 9%)
2018	LAMBAYEQUE	LAMBAYEQUE	4968	3317 (3305 - 3329)	3% (3% - 4%)	4% (4% - 5%)
2019	LAMBAYEQUE	CHICLAYO	13490	3230 (3220 - 3240)	9% (8% - 9%)	4% (4% - 5%)
2019	LAMBAYEQUE	FERREÑAFE	764	3290 (3262 - 3318)	2% (1% - 4%)	6% (4% - 8%)
2019	LAMBAYEQUE	LAMBAYEQUE	4730	3296 (3282 - 3309)	4% (4% - 5%)	5% (5% - 6%)
2012	LIMA	HUAURA	1681	3323 (3298 - 3348)	6% (4% - 7%)	5% (4% - 6%)
2012	LIMA	LIMA	49270	3311 (3306 - 3316)	7% (7% - 7%)	5% (5% - 5%)
2013	LIMA	CAÑETE	1646	3358 (3332 - 3384)	5% (4% - 6%)	4% (3% - 5%)
2013	LIMA	HUARAL	801	3375 (3341 - 3409)	4% (2% - 5%)	5% (3% - 6%)
2013	LIMA	HUAURA	2964	3319 (3300 - 3337)	5% (4% - 6%)	3% (2% - 3%)
2013	LIMA	LIMA	94116	3327 (3323 - 3330)	6% (6% - 6%)	4% (4% - 5%)
2014	LIMA	CAÑETE	2514	3330 (3309 - 3350)	5% (5% - 6%)	4% (3% - 5%)
2014	LIMA	HUARAL	1945	3368 (3346 - 3389)	4% (3% - 4%)	4% (3% - 5%)
2014	LIMA	HUAROCHIRI	130	3218 (3141 - 3295)	5% (2% - 11%)	8% (4% - 15%)
2014	LIMA	HUAURA	2983	3341 (3323 - 3359)	5% (4% - 6%)	4% (3% - 4%)
2014	LIMA	LIMA	108534	3336 (3333 - 3339)	5% (5% - 6%)	4% (4% - 4%)
2015	LIMA	BARRANCA	1364	3377 (3349 - 3404)	4% (3% - 5%)	3% (2% - 4%)
2015	LIMA	CAÑETE	2999	3349 (3330 - 3367)	5% (4% - 6%)	6% (5% - 6%)
2015	LIMA	HUARAL	2822	3341 (3321 - 3360)	5% (4% - 6%)	5% (4% - 6%)
2015	LIMA	HUAROCHIRI	172	3251 (3190 - 3312)	3% (1% - 7%)	4% (2% - 8%)

2015	LIMA	HUAURA	2969	3301 (3283 - 3319)	5% (4% - 6%)	4% (4% - 5%)
2015	LIMA	LIMA	131977	3338 (3335 - 3341)	5% (5% - 6%)	4% (4% - 4%)
2016	LIMA	BARRANCA	2225	3377 (3357 - 3397)	3% (3% - 4%)	3% (3% - 4%)
2016	LIMA	CAÑETE	4123	3371 (3356 - 3386)	4% (3% - 4%)	4% (4% - 5%)
2016	LIMA	HUARAL	2673	3354 (3335 - 3372)	4% (3% - 5%)	4% (3% - 5%)
2016	LIMA	HUAROCHIRI	241	3278 (3229 - 3327)	2% (1% - 5%)	2% (1% - 5%)
2016	LIMA	HUAURA	3082	3335 (3317 - 3353)	5% (4% - 5%)	3% (3% - 4%)
2016	LIMA	LIMA	139012	3331 (3328 - 3334)	5% (5% - 6%)	4% (4% - 4%)
2017	LIMA	BARRANCA	2219	3376 (3356 - 3396)	4% (3% - 5%)	3% (3% - 4%)
2017	LIMA	CAÑETE	4248	3368 (3354 - 3382)	4% (3% - 4%)	4% (4% - 5%)
2017	LIMA	HUARAL	2706	3360 (3341 - 3379)	4% (4% - 5%)	4% (3% - 5%)
2017	LIMA	HUAROCHIRI	202	3304 (3249 - 3359)	2% (1% - 5%)	3% (1% - 6%)
2017	LIMA	HUAURA	3498	3341 (3325 - 3358)	4% (4% - 5%)	3% (2% - 4%)
2017	LIMA	LIMA	140043	3334 (3331 - 3336)	5% (5% - 6%)	4% (4% - 4%)
2018	LIMA	BARRANCA	2219	3397 (3376 - 3417)	3% (3% - 4%)	2% (2% - 3%)
2018	LIMA	CAÑETE	4102	3368 (3352 - 3383)	4% (4% - 5%)	4% (4% - 5%)
2018	LIMA	HUARAL	2740	3354 (3336 - 3372)	4% (3% - 5%)	3% (3% - 4%)
2018	LIMA	HUAROCHIRI	217	3274 (3227 - 3321)	2% (1% - 5%)	4% (2% - 7%)
2018	LIMA	HUAURA	4767	3395 (3382 - 3408)	3% (2% - 3%)	2% (2% - 3%)
2018	LIMA	LIMA	148250	3330 (3328 - 3333)	6% (6% - 6%)	4% (4% - 4%)
2019	LIMA	BARRANCA	1978	3361 (3339 - 3383)	5% (4% - 6%)	3% (3% - 4%)
2019	LIMA	CAÑETE	3939	3382 (3366 - 3397)	3% (3% - 4%)	3% (3% - 4%)
2019	LIMA	HUARAL	2702	3360 (3342 - 3378)	4% (3% - 5%)	3% (3% - 4%)
2019	LIMA	HUAROCHIRI	232	3253 (3201 - 3306)	2% (1% - 5%)	2% (0% - 4%)
2019	LIMA	HUAURA	4492	3366 (3352 - 3380)	4% (3% - 4%)	3% (2% - 3%)
2019	LIMA	LIMA	144037	3323 (3320 - 3325)	6% (6% - 6%)	4% (4% - 4%)
2014	LORETO	ALTO AMAZONAS	728	3119 (3083 - 3155)	9% (7% - 12%)	12% (10% - 14%)
2014	LORETO	DATEM DEL MARAÑON	142	3035 (2953 - 3117)	14% (9% - 21%)	18% (12% - 25%)
2014	LORETO	LORETO	177	3178 (3109 - 3246)	6% (3% - 10%)	12% (7% - 18%)
2014	LORETO	MARISCAL RAMON CASTILLA	86	3191 (3099 - 3283)	8% (3% - 16%)	8% (3% - 16%)

2014	LORETO	MAYNAS	2917	3113 (3095 - 3131)	9% (8% - 10%)	8% (7% - 9%)
2015	LORETO	ALTO AMAZONAS	2237	3085 (3064 - 3106)	10% (9% - 11%)	13% (11% - 14%)
2015	LORETO	DATEM DEL MARAÑON	253	3105 (3050 - 3160)	9% (6% - 13%)	15% (11% - 20%)
2015	LORETO	LORETO	448	3191 (3154 - 3229)	4% (3% - 7%)	9% (7% - 12%)
2015	LORETO	MARISCAL RAMON CASTILLA	265	3190 (3140 - 3240)	6% (3% - 9%)	7% (4% - 11%)
2015	LORETO	MAYNAS	8506	3133 (3122 - 3144)	9% (8% - 9%)	8% (8% - 9%)
2015	LORETO	UCAYALI	41	3174 (3017 - 3331)	7% (2% - 20%)	15% (6% - 29%)
2016	LORETO	ALTO AMAZONAS	2472	3103 (3083 - 3122)	8% (7% - 9%)	10% (9% - 11%)
2016	LORETO	DATEM DEL MARAÑON	361	3104 (3056 - 3152)	7% (5% - 10%)	11% (8% - 14%)
2016	LORETO	LORETO	490	3139 (3096 - 3181)	6% (4% - 8%)	9% (6% - 12%)
2016	LORETO	MARISCAL RAMON CASTILLA	504	3189 (3149 - 3228)	6% (4% - 8%)	10% (8% - 13%)
2016	LORETO	MAYNAS	10274	3140 (3129 - 3150)	9% (9% - 10%)	7% (7% - 7%)
2016	LORETO	REQUENA	618	3237 (3202 - 3272)	3% (2% - 4%)	6% (4% - 8%)
2016	LORETO	UCAYALI	436	3122 (3078 - 3167)	7% (5% - 10%)	12% (9% - 15%)
2017	LORETO	ALTO AMAZONAS	3397	3079 (3064 - 3094)	7% (6% - 8%)	8% (7% - 9%)
2017	LORETO	DATEM DEL MARAÑON	487	3097 (3053 - 3142)	7% (5% - 9%)	10% (8% - 13%)
2017	LORETO	LORETO	667	3135 (3102 - 3168)	5% (3% - 7%)	10% (8% - 12%)
2017	LORETO	MARISCAL RAMON CASTILLA	910	3225 (3195 - 3255)	4% (3% - 5%)	8% (7% - 10%)
2017	LORETO	MAYNAS	10908	3124 (3114 - 3133)	9% (9% - 10%)	7% (6% - 7%)
2017	LORETO	PUTUMAYO	58	3230 (3131 - 3330)	2% (0% - 9%)	9% (3% - 19%)
2017	LORETO	REQUENA	704	3251 (3216 - 3285)	4% (2% - 5%)	7% (5% - 9%)
2017	LORETO	UCAYALI	842	3191 (3159 - 3222)	6% (4% - 7%)	9% (7% - 11%)
2018	LORETO	ALTO AMAZONAS	3294	3080 (3063 - 3096)	8% (7% - 9%)	9% (8% - 10%)
2018	LORETO	DATEM DEL MARAÑON	584	3072 (3036 - 3109)	8% (6% - 11%)	10% (7% - 12%)
2018	LORETO	LORETO	661	3168 (3133 - 3202)	5% (3% - 7%)	10% (8% - 13%)
2018	LORETO	MARISCAL RAMON CASTILLA	973	3204 (3176 - 3232)	4% (3% - 6%)	9% (8% - 11%)
2018	LORETO	MAYNAS	10925	3131 (3122 - 3141)	8% (8% - 9%)	6% (6% - 7%)
2018	LORETO	PUTUMAYO	52	3242 (3125 - 3358)	6% (1% - 16%)	6% (1% - 16%)
2018	LORETO	REQUENA	801	3149 (3117 - 3181)	6% (4% - 7%)	8% (7% - 11%)
2018	LORETO	UCAYALI	712	3177 (3142 - 3212)	6% (5% - 8%)	10% (8% - 13%)

2019	LORETO	ALTO AMAZONAS	3318	3085 (3069 - 3101)	8% (7% - 9%)	11% (10% - 12%)
2019	LORETO	DATEM DEL MARAÑON	538	3089 (3050 - 3127)	8% (6% - 10%)	13% (10% - 16%)
2019	LORETO	LORETO	745	3145 (3114 - 3176)	5% (3% - 6%)	8% (6% - 10%)
2019	LORETO	MARISCAL RAMON CASTILLA	943	3203 (3174 - 3232)	4% (3% - 6%)	9% (7% - 11%)
2019	LORETO	MAYNAS	10895	3135 (3125 - 3145)	8% (8% - 9%)	6% (6% - 7%)
2019	LORETO	PUTUMAYO	46	3043 (2931 - 3155)	7% (1% - 18%)	9% (2% - 21%)
2019	LORETO	REQUENA	665	3194 (3160 - 3227)	5% (3% - 7%)	7% (6% - 10%)
2019	LORETO	UCAYALI	800	3129 (3098 - 3161)	7% (5% - 9%)	10% (8% - 12%)
2012	MADRE DE DIOS	TAMBOPATA	73	3308 (3189 - 3428)	5% (2% - 13%)	4% (1% - 12%)
2013	MADRE DE DIOS	TAMBOPATA	1085	3376 (3343 - 3408)	5% (4% - 6%)	5% (4% - 6%)
2014	MADRE DE DIOS	TAHUAMANU	100	3425 (3328 - 3521)	2% (0% - 7%)	4% (1% - 10%)
2014	MADRE DE DIOS	TAMBOPATA	2057	3360 (3337 - 3382)	5% (4% - 6%)	4% (4% - 5%)
2015	MADRE DE DIOS	TAHUAMANU	137	3392 (3314 - 3470)	3% (1% - 7%)	3% (1% - 7%)
2015	MADRE DE DIOS	TAMBOPATA	2677	3361 (3341 - 3381)	5% (4% - 5%)	4% (3% - 5%)
2016	MADRE DE DIOS	MANU	37	3375 (3213 - 3537)	3% (0% - 14%)	3% (0% - 14%)
2016	MADRE DE DIOS	TAHUAMANU	110	3412 (3339 - 3486)	2% (0% - 6%)	4% (1% - 9%)
2016	MADRE DE DIOS	TAMBOPATA	2883	3354 (3335 - 3374)	5% (4% - 6%)	5% (4% - 5%)
2017	MADRE DE DIOS	MANU	123	3466 (3376 - 3556)	2% (0% - 6%)	1% (0% - 4%)
2017	MADRE DE DIOS	TAHUAMANU	132	3327 (3251 - 3404)	4% (1% - 9%)	4% (1% - 9%)
2017	MADRE DE DIOS	TAMBOPATA	3409	3378 (3361 - 3396)	5% (4% - 6%)	4% (4% - 5%)
2018	MADRE DE DIOS	MANU	107	3474 (3389 - 3558)		3% (1% - 8%)
2018	MADRE DE DIOS	TAHUAMANU	127	3383 (3299 - 3467)	3% (1% - 8%)	2% (0% - 7%)
2018	MADRE DE DIOS	TAMBOPATA	3579	3392 (3375 - 3409)	4% (4% - 5%)	5% (4% - 6%)
2019	MADRE DE DIOS	MANU	82	3483 (3383 - 3583)	1% (0% - 7%)	6% (2% - 14%)
2019	MADRE DE DIOS	TAHUAMANU	133	3362 (3284 - 3439)	3% (1% - 8%)	5% (2% - 10%)
2019	MADRE DE DIOS	TAMBOPATA	3523	3379 (3362 - 3396)	4% (4% - 5%)	5% (4% - 5%)
2013	MOQUEGUA	ILO	668	3537 (3499 - 3574)	1% (1% - 3%)	3% (2% - 4%)
2013	MOQUEGUA	MARISCAL NIETO	700	3373 (3334 - 3413)	6% (4% - 8%)	3% (2% - 5%)
2014	MOQUEGUA	ILO	1174	3494 (3466 - 3522)	2% (1% - 3%)	3% (2% - 4%)
2014	MOQUEGUA	MARISCAL NIETO	1272	3437 (3411 - 3463)	3% (2% - 4%)	3% (2% - 4%)

2015	MOQUEGUA	GENERAL SANCHEZ CERRO	35	3149 (3034 - 3265)	3% (0% - 15%)	3% (0% - 15%)
2015	MOQUEGUA	ILO	1192	3491 (3465 - 3517)	2% (1% - 3%)	3% (2% - 4%)
2015	MOQUEGUA	MARISCAL NIETO	1350	3443 (3419 - 3467)	3% (2% - 4%)	2% (1% - 3%)
2016	MOQUEGUA	GENERAL SANCHEZ CERRO	43	3228 (3067 - 3389)	5% (1% - 16%)	5% (1% - 16%)
2016	MOQUEGUA	ILO	1089	3526 (3496 - 3555)	2% (1% - 3%)	2% (2% - 4%)
2016	MOQUEGUA	MARISCAL NIETO	1315	3458 (3432 - 3484)	2% (2% - 3%)	2% (1% - 3%)
2017	MOQUEGUA	ILO	1080	3507 (3479 - 3534)	1% (1% - 2%)	1% (1% - 2%)
2017	MOQUEGUA	MARISCAL NIETO	1322	3477 (3451 - 3504)	2% (2% - 3%)	3% (2% - 4%)
2018	MOQUEGUA	ILO	994	3522 (3493 - 3550)	2% (1% - 2%)	2% (1% - 3%)
2018	MOQUEGUA	MARISCAL NIETO	1217	3465 (3438 - 3492)	3% (2% - 4%)	2% (1% - 3%)
2019	MOQUEGUA	ILO	1031	3504 (3475 - 3533)	2% (1% - 3%)	2% (1% - 3%)
2019	MOQUEGUA	MARISCAL NIETO	1229	3455 (3429 - 3482)	2% (2% - 3%)	1% (1% - 2%)
2013	PASCO	PASCO	103	2918 (2823 - 3013)	12% (6% - 19%)	18% (11% - 27%)
2014	PASCO	OXAPAMPA	574	3255 (3217 - 3293)	3% (2% - 5%)	4% (3% - 6%)
2014	PASCO	PASCO	1635	2936 (2915 - 2957)	13% (12% - 15%)	15% (13% - 17%)
2015	PASCO	DANIEL ALCIDES CARRION	157	3166 (3102 - 3231)	5% (2% - 10%)	7% (4% - 12%)
2015	PASCO	OXAPAMPA	1517	3198 (3175 - 3221)	6% (5% - 7%)	8% (6% - 9%)
2015	PASCO	PASCO	2543	2961 (2944 - 2979)	12% (11% - 14%)	15% (14% - 16%)
2016	PASCO	DANIEL ALCIDES CARRION	176	3167 (3101 - 3233)	7% (4% - 12%)	9% (5% - 14%)
2016	PASCO	OXAPAMPA	1730	3205 (3182 - 3227)	5% (4% - 6%)	7% (6% - 8%)
2016	PASCO	PASCO	2450	2977 (2959 - 2995)	12% (11% - 14%)	13% (12% - 15%)
2017	PASCO	DANIEL ALCIDES CARRION	181	3084 (3020 - 3148)	6% (3% - 10%)	8% (5% - 13%)
2017	PASCO	OXAPAMPA	1939	3171 (3152 - 3191)	5% (4% - 6%)	8% (7% - 9%)
2017	PASCO	PASCO	2488	2981 (2964 - 2998)	11% (10% - 12%)	13% (12% - 15%)
2018	PASCO	DANIEL ALCIDES CARRION	181	3061 (3002 - 3120)	6% (3% - 10%)	13% (8% - 18%)
2018	PASCO	OXAPAMPA	1705	3179 (3157 - 3201)	5% (4% - 6%)	8% (7% - 10%)
2018	PASCO	PASCO	2282	2986 (2967 - 3005)	12% (11% - 13%)	12% (10% - 13%)
2019	PASCO	DANIEL ALCIDES CARRION	195	3058 (3001 - 3115)	5% (2% - 9%)	12% (8% - 18%)
2019	PASCO	OXAPAMPA	1781	3189 (3167 - 3210)	5% (4% - 6%)	8% (7% - 10%)
2019	PASCO	PASCO	2225	2993 (2974 - 3013)	12% (11% - 13%)	13% (12% - 15%)

2012	PIURA	AYABACA	161	3067 (3013 - 3121)	2% (0% - 5%)	16% (10% - 22%)
2012	PIURA	PIURA	2608	3280 (3261 - 3300)	4% (4% - 5%)	6% (5% - 7%)
2012	PIURA	SULLANA	182	3168 (3090 - 3245)	8% (4% - 13%)	7% (3% - 11%)
2013	PIURA	AYABACA	118	3101 (3024 - 3177)	8% (4% - 14%)	16% (10% - 24%)
2013	PIURA	MORROPON	914	3208 (3178 - 3238)	7% (5% - 8%)	8% (6% - 10%)
2013	PIURA	PIURA	3877	3281 (3264 - 3298)	6% (5% - 6%)	7% (6% - 8%)
2013	PIURA	SECHURA	207	3193 (3131 - 3255)	5% (2% - 9%)	6% (3% - 10%)
2013	PIURA	SULLANA	4122	3142 (3124 - 3160)	11% (10% - 12%)	9% (8% - 9%)
2013	PIURA	TALARA	114	3246 (3168 - 3324)	4% (1% - 10%)	4% (1% - 9%)
2014	PIURA	AYABACA	179	3103 (3045 - 3162)	6% (3% - 11%)	13% (9% - 19%)
2014	PIURA	HUANCABAMBA	38	3301 (3170 - 3432)	3% (0% - 14%)	5% (1% - 18%)
2014	PIURA	MORROPON	1707	3178 (3154 - 3202)	7% (5% - 8%)	11% (9% - 12%)
2014	PIURA	PAITA	948	3285 (3258 - 3312)	3% (2% - 4%)	5% (4% - 7%)
2014	PIURA	PIURA	7785	3180 (3167 - 3193)	10% (9% - 10%)	7% (6% - 7%)
2014	PIURA	SECHURA	352	3204 (3159 - 3249)	4% (2% - 7%)	8% (5% - 11%)
2014	PIURA	SULLANA	4417	3177 (3160 - 3194)	10% (9% - 11%)	9% (8% - 9%)
2014	PIURA	TALARA	706	3322 (3289 - 3355)	2% (1% - 4%)	4% (3% - 6%)
2015	PIURA	AYABACA	444	3106 (3065 - 3147)	5% (3% - 7%)	14% (11% - 18%)
2015	PIURA	HUANCABAMBA	503	3181 (3139 - 3222)	6% (4% - 8%)	8% (6% - 11%)
2015	PIURA	MORROPON	2059	3240 (3220 - 3261)	5% (4% - 6%)	7% (6% - 8%)
2015	PIURA	PAITA	1381	3261 (3238 - 3284)	3% (2% - 4%)	4% (3% - 5%)
2015	PIURA	PIURA	9675	3177 (3165 - 3189)	9% (9% - 10%)	7% (7% - 8%)
2015	PIURA	SECHURA	598	3221 (3186 - 3255)	4% (2% - 6%)	6% (4% - 8%)
2015	PIURA	SULLANA	4310	3173 (3157 - 3189)	10% (9% - 11%)	8% (7% - 9%)
2015	PIURA	TALARA	929	3324 (3294 - 3354)	3% (2% - 4%)	5% (3% - 6%)
2016	PIURA	AYABACA	712	3210 (3177 - 3243)	4% (2% - 5%)	10% (8% - 13%)
2016	PIURA	HUANCABAMBA	745	3165 (3132 - 3198)	6% (4% - 8%)	10% (8% - 13%)
2016	PIURA	MORROPON	2313	3253 (3233 - 3272)	4% (4% - 5%)	6% (5% - 7%)
2016	PIURA	PAITA	1598	3276 (3254 - 3299)	3% (2% - 4%)	5% (4% - 6%)
2016	PIURA	PIURA	10825	3195 (3185 - 3206)	9% (8% - 9%)	6% (6% - 7%)

2016	PIURA	SECHURA	902	3215 (3189 - 3241)	4% (3% - 5%)	4% (3% - 6%)
2016	PIURA	SULLANA	4774	3160 (3143 - 3176)	11% (10% - 12%)	8% (7% - 9%)
2016	PIURA	TALARA	754	3320 (3287 - 3352)	3% (2% - 4%)	3% (2% - 5%)
2017	PIURA	AYABACA	834	3209 (3179 - 3238)	4% (3% - 5%)	7% (6% - 9%)
2017	PIURA	HUANCABAMBA	744	3191 (3161 - 3220)	4% (3% - 5%)	8% (6% - 11%)
2017	PIURA	MORROPON	2332	3253 (3233 - 3272)	5% (4% - 6%)	6% (5% - 7%)
2017	PIURA	PAITA	1773	3280 (3259 - 3301)	4% (3% - 5%)	4% (3% - 5%)
2017	PIURA	PIURA	11248	3193 (3182 - 3203)	8% (8% - 9%)	7% (6% - 7%)
2017	PIURA	SECHURA	633	3210 (3179 - 3241)	4% (2% - 6%)	6% (4% - 8%)
2017	PIURA	SULLANA	5714	3192 (3178 - 3207)	9% (8% - 10%)	7% (6% - 7%)
2017	PIURA	TALARA	701	3303 (3270 - 3336)	3% (2% - 4%)	5% (4% - 7%)
2018	PIURA	AYABACA	796	3194 (3163 - 3225)	4% (2% - 5%)	8% (6% - 10%)
2018	PIURA	HUANCABAMBA	793	3130 (3101 - 3159)	4% (3% - 6%)	10% (8% - 12%)
2018	PIURA	MORROPON	2417	3283 (3264 - 3303)	5% (4% - 6%)	6% (5% - 7%)
2018	PIURA	PAITA	1950	3309 (3290 - 3329)	3% (2% - 4%)	4% (3% - 5%)
2018	PIURA	PIURA	12484	3227 (3217 - 3236)	8% (7% - 8%)	6% (6% - 6%)
2018	PIURA	SECHURA	608	3244 (3212 - 3276)	3% (2% - 5%)	4% (3% - 6%)
2018	PIURA	SULLANA	6698	3241 (3228 - 3254)	7% (6% - 8%)	5% (4% - 6%)
2018	PIURA	TALARA	1468	3323 (3300 - 3347)	3% (2% - 4%)	4% (3% - 5%)
2019	PIURA	AYABACA	782	3160 (3129 - 3191)	5% (4% - 7%)	11% (9% - 14%)
2019	PIURA	HUANCABAMBA	764	3160 (3129 - 3190)	4% (3% - 6%)	10% (8% - 12%)
2019	PIURA	MORROPON	2400	3284 (3264 - 3303)	5% (4% - 6%)	6% (5% - 6%)
2019	PIURA	PAITA	1902	3281 (3260 - 3302)	4% (3% - 5%)	5% (4% - 6%)
2019	PIURA	PIURA	11704	3206 (3196 - 3217)	9% (8% - 9%)	5% (5% - 6%)
2019	PIURA	SECHURA	632	3176 (3143 - 3208)	4% (3% - 6%)	7% (5% - 9%)
2019	PIURA	SULLANA	6595	3217 (3204 - 3230)	8% (7% - 8%)	5% (4% - 5%)
2019	PIURA	TALARA	1249	3322 (3298 - 3346)	2% (1% - 3%)	3% (2% - 5%)
2012	PUNO	PUNO	68	3189 (3062 - 3316)	9% (3% - 18%)	7% (2% - 16%)
2012	PUNO	SAN ROMAN	164	3143 (3063 - 3222)	9% (5% - 14%)	5% (2% - 9%)
2013	PUNO	PUNO	2179	3151 (3131 - 3172)	8% (7% - 9%)	7% (6% - 8%)

2013	PUNO	SAN ROMAN	2749	3094 (3072 - 3115)	12% (11% - 14%)	10% (9% - 11%)
2014	PUNO	AZANGARO	36	3035 (2889 - 3181)	11% (3% - 26%)	14% (5% - 29%)
2014	PUNO	CARABAYA	171	3026 (2965 - 3087)	6% (3% - 11%)	9% (5% - 15%)
2014	PUNO	CHUCUITO	212	3198 (3139 - 3257)	5% (2% - 9%)	6% (3% - 10%)
2014	PUNO	EL COLLAO	454	3296 (3262 - 3329)	1% (0% - 2%)	4% (3% - 7%)
2014	PUNO	HUANCANE	180	3160 (3100 - 3220)	5% (2% - 9%)	10% (6% - 15%)
2014	PUNO	LAMPA	105	3029 (2944 - 3114)	10% (5% - 18%)	10% (5% - 18%)
2014	PUNO	MELGAR	392	3112 (3068 - 3157)	7% (5% - 10%)	5% (3% - 8%)
2014	PUNO	PUNO	2289	3159 (3138 - 3180)	7% (6% - 9%)	7% (6% - 8%)
2014	PUNO	SAN ROMAN	3619	3118 (3100 - 3136)	11% (10% - 12%)	9% (8% - 10%)
2014	PUNO	SANDIA	90	3360 (3256 - 3464)	2% (0% - 8%)	6% (2% - 12%)
2014	PUNO	YUNGUYO	154	3197 (3132 - 3262)	5% (2% - 9%)	6% (3% - 12%)
2015	PUNO	AZANGARO	684	3167 (3136 - 3197)	5% (4% - 7%)	6% (5% - 8%)
2015	PUNO	CARABAYA	329	3073 (3027 - 3119)	6% (4% - 10%)	10% (7% - 14%)
2015	PUNO	CHUCUITO	617	3191 (3157 - 3224)	5% (3% - 6%)	8% (6% - 10%)
2015	PUNO	EL COLLAO	814	3296 (3269 - 3323)	2% (1% - 3%)	4% (3% - 5%)
2015	PUNO	HUANCANE	444	3148 (3107 - 3188)	5% (3% - 7%)	9% (7% - 12%)
2015	PUNO	LAMPA	216	3175 (3119 - 3230)	5% (2% - 8%)	8% (5% - 13%)
2015	PUNO	MELGAR	729	3102 (3072 - 3131)	7% (5% - 9%)	6% (4% - 8%)
2015	PUNO	MOHO	35	3040 (2940 - 3140)		9% (2% - 23%)
2015	PUNO	PUNO	3070	3167 (3148 - 3185)	8% (7% - 9%)	8% (7% - 9%)
2015	PUNO	SAN ROMAN	4569	3141 (3126 - 3157)	10% (9% - 11%)	9% (8% - 10%)
2015	PUNO	SANDIA	195	3473 (3407 - 3539)	2% (1% - 5%)	2% (1% - 5%)
2015	PUNO	YUNGUYO	360	3218 (3176 - 3261)	3% (2% - 6%)	8% (5% - 11%)
2016	PUNO	AZANGARO	1217	3135 (3112 - 3157)	6% (4% - 7%)	7% (6% - 9%)
2016	PUNO	CARABAYA	463	3136 (3097 - 3174)	5% (3% - 8%)	9% (7% - 12%)
2016	PUNO	CHUCUITO	852	3242 (3213 - 3270)	4% (3% - 5%)	4% (3% - 6%)
2016	PUNO	EL COLLAO	752	3292 (3263 - 3320)	2% (1% - 4%)	4% (3% - 6%)
2016	PUNO	HUANCANE	598	3219 (3185 - 3252)	4% (2% - 6%)	7% (5% - 9%)
2016	PUNO	LAMPA	266	3222 (3170 - 3274)	3% (2% - 6%)	5% (3% - 8%)

2016	PUNO	MELGAR	912	3140 (3113 - 3167)	5% (4% - 7%)	5% (4% - 7%)
2016	PUNO	MOHO	82	3073 (2980 - 3165)	6% (2% - 14%)	11% (5% - 20%)
2016	PUNO	PUNO	3417	3198 (3181 - 3214)	7% (6% - 8%)	6% (5% - 7%)
2016	PUNO	SAN ANTONIO DE PUTINA	80	3187 (3086 - 3288)	6% (2% - 14%)	14% (7% - 23%)
2016	PUNO	SAN ROMAN	5951	3169 (3156 - 3182)	7% (7% - 8%)	7% (6% - 7%)
2016	PUNO	SANDIA	396	3381 (3336 - 3425)	3% (1% - 5%)	4% (3% - 7%)
2016	PUNO	YUNGUYO	384	3238 (3197 - 3279)	5% (3% - 8%)	5% (3% - 8%)
2017	PUNO	AZANGARO	1146	3147 (3123 - 3171)	5% (4% - 7%)	8% (6% - 9%)
2017	PUNO	CARABAYA	523	3128 (3088 - 3167)	6% (4% - 8%)	8% (6% - 10%)
2017	PUNO	CHUCUITO	773	3234 (3204 - 3264)	3% (2% - 5%)	6% (5% - 8%)
2017	PUNO	EL COLLAO	732	3318 (3289 - 3348)	2% (1% - 4%)	5% (3% - 7%)
2017	PUNO	HUANCANE	595	3230 (3197 - 3262)	3% (1% - 4%)	6% (4% - 8%)
2017	PUNO	LAMPA	243	3170 (3119 - 3221)	4% (2% - 7%)	6% (3% - 9%)
2017	PUNO	MELGAR	876	3133 (3105 - 3161)	6% (5% - 8%)	5% (4% - 7%)
2017	PUNO	MOHO	79	3213 (3132 - 3294)	1% (0% - 7%)	3% (0% - 9%)
2017	PUNO	PUNO	3531	3184 (3167 - 3200)	7% (7% - 8%)	6% (5% - 7%)
2017	PUNO	SAN ANTONIO DE PUTINA	245	3127 (3080 - 3173)	6% (3% - 10%)	7% (4% - 11%)
2017	PUNO	SAN ROMAN	6123	3145 (3132 - 3157)	8% (8% - 9%)	7% (6% - 8%)
2017	PUNO	SANDIA	465	3391 (3350 - 3433)	2% (1% - 4%)	4% (2% - 6%)
2017	PUNO	YUNGUYO	346	3253 (3206 - 3300)	4% (2% - 7%)	10% (7% - 13%)
2018	PUNO	AZANGARO	1111	3177 (3152 - 3201)	4% (3% - 6%)	7% (5% - 8%)
2018	PUNO	CARABAYA	662	3138 (3107 - 3168)	4% (3% - 6%)	7% (5% - 9%)
2018	PUNO	CHUCUITO	723	3249 (3217 - 3281)	4% (2% - 5%)	5% (4% - 7%)
2018	PUNO	EL COLLAO	762	3284 (3255 - 3314)	2% (1% - 4%)	4% (3% - 6%)
2018	PUNO	HUANCANE	644	3195 (3165 - 3226)	3% (2% - 5%)	6% (4% - 8%)
2018	PUNO	LAMPA	275	3174 (3127 - 3220)	4% (2% - 7%)	5% (3% - 8%)
2018	PUNO	MELGAR	886	3149 (3123 - 3175)	5% (4% - 7%)	5% (4% - 7%)
2018	PUNO	MOHO	82	3158 (3064 - 3251)	7% (3% - 15%)	6% (2% - 14%)
2018	PUNO	PUNO	3325	3196 (3179 - 3213)	7% (6% - 8%)	5% (5% - 6%)
2018	PUNO	SAN ANTONIO DE PUTINA	226	3113 (3060 - 3166)	7% (4% - 11%)	11% (7% - 15%)

2018	PUNO	SAN ROMAN	6326	3146 (3134 - 3158)	8% (7% - 9%)	7% (7% - 8%)
2018	PUNO	SANDIA	489	3427 (3390 - 3463)	2% (1% - 4%)	2% (1% - 4%)
2018	PUNO	YUNGUYO	330	3219 (3172 - 3267)	4% (2% - 6%)	7% (5% - 11%)
2019	PUNO	AZANGARO	1045	3132 (3106 - 3158)	6% (5% - 8%)	8% (7% - 10%)
2019	PUNO	CARABAYA	592	3133 (3100 - 3165)	5% (3% - 7%)	7% (5% - 10%)
2019	PUNO	CHUCUITO	688	3225 (3194 - 3256)	4% (3% - 6%)	5% (4% - 7%)
2019	PUNO	EL COLLAO	644	3273 (3241 - 3304)	2% (1% - 4%)	5% (4% - 7%)
2019	PUNO	HUANCANE	523	3191 (3154 - 3228)	4% (3% - 7%)	7% (5% - 10%)
2019	PUNO	LAMPA	268	3146 (3101 - 3191)	3% (1% - 6%)	7% (4% - 10%)
2019	PUNO	MELGAR	830	3156 (3128 - 3185)	5% (4% - 7%)	5% (4% - 7%)
2019	PUNO	MOHO	47	3295 (3206 - 3383)		
2019	PUNO	PUNO	3305	3202 (3185 - 3218)	6% (6% - 7%)	6% (5% - 7%)
2019	PUNO	SAN ANTONIO DE PUTINA	164	3189 (3127 - 3250)	5% (2% - 9%)	8% (4% - 13%)
2019	PUNO	SAN ROMAN	6598	3142 (3130 - 3154)	8% (8% - 9%)	7% (7% - 8%)
2019	PUNO	SANDIA	433	3387 (3343 - 3431)	3% (2% - 5%)	3% (2% - 5%)
2019	PUNO	YUNGUYO	352	3250 (3210 - 3291)	3% (2% - 6%)	7% (4% - 10%)
2012	SAN MARTIN	SAN MARTIN	101	3149 (3048 - 3250)	11% (6% - 19%)	8% (3% - 15%)
2013	SAN MARTIN	SAN MARTIN	2536	3102 (3079 - 3125)	12% (11% - 13%)	10% (9% - 11%)
2014	SAN MARTIN	MOYOBAMBA	187	3297 (3235 - 3358)	2% (1% - 5%)	4% (2% - 8%)
2014	SAN MARTIN	SAN MARTIN	2948	3115 (3093 - 3136)	12% (11% - 14%)	7% (6% - 8%)
2015	SAN MARTIN	BELLAVISTA	369	3267 (3217 - 3317)	5% (3% - 7%)	4% (2% - 7%)
2015	SAN MARTIN	EL DORADO	420	3075 (3035 - 3114)	6% (4% - 8%)	10% (7% - 13%)
2015	SAN MARTIN	LAMAS	316	3140 (3092 - 3187)	6% (4% - 10%)	9% (6% - 13%)
2015	SAN MARTIN	MARISCAL CACERES	791	3248 (3219 - 3278)	3% (2% - 4%)	5% (4% - 7%)
2015	SAN MARTIN	MOYOBAMBA	2061	3261 (3241 - 3281)	5% (4% - 6%)	6% (5% - 7%)
2015	SAN MARTIN	RIOJA	1760	3272 (3251 - 3293)	4% (3% - 5%)	7% (6% - 8%)
2015	SAN MARTIN	SAN MARTIN	4264	3150 (3133 - 3167)	11% (10% - 12%)	8% (7% - 9%)
2015	SAN MARTIN	TOCACHE	845	3289 (3259 - 3319)	4% (3% - 6%)	6% (4% - 8%)
2016	SAN MARTIN	BELLAVISTA	500	3242 (3204 - 3279)	4% (2% - 6%)	7% (5% - 10%)
2016	SAN MARTIN	EL DORADO	540	3145 (3105 - 3186)	6% (5% - 9%)	10% (7% - 12%)

2016	SAN MARTIN	HUALLAGA	201	3376 (3317 - 3435)	2% (1% - 5%)	1% (0% - 4%)
2016	SAN MARTIN	LAMAS	756	3163 (3133 - 3193)	5% (4% - 7%)	6% (5% - 8%)
2016	SAN MARTIN	MARISCAL CACERES	943	3254 (3225 - 3282)	4% (3% - 5%)	7% (6% - 9%)
2016	SAN MARTIN	MOYOBAMBA	2485	3239 (3221 - 3256)	5% (4% - 6%)	6% (5% - 7%)
2016	SAN MARTIN	PICOTA	332	3230 (3188 - 3272)	3% (1% - 5%)	5% (3% - 8%)
2016	SAN MARTIN	RIOJA	2243	3271 (3251 - 3290)	5% (4% - 6%)	7% (6% - 8%)
2016	SAN MARTIN	SAN MARTIN	4848	3150 (3134 - 3166)	10% (10% - 11%)	7% (6% - 8%)
2016	SAN MARTIN	TOCACHE	1194	3280 (3253 - 3306)	4% (3% - 5%)	6% (4% - 7%)
2017	SAN MARTIN	BELLAVISTA	600	3253 (3220 - 3287)	2% (1% - 4%)	5% (3% - 7%)
2017	SAN MARTIN	EL DORADO	488	3170 (3128 - 3211)	5% (3% - 7%)	7% (5% - 10%)
2017	SAN MARTIN	HUALLAGA	166	3344 (3280 - 3408)	2% (1% - 6%)	5% (2% - 9%)
2017	SAN MARTIN	LAMAS	833	3163 (3134 - 3191)	3% (2% - 5%)	7% (5% - 9%)
2017	SAN MARTIN	MARISCAL CACERES	1008	3265 (3238 - 3292)	3% (2% - 5%)	6% (4% - 7%)
2017	SAN MARTIN	MOYOBAMBA	2406	3268 (3249 - 3288)	4% (4% - 5%)	5% (4% - 6%)
2017	SAN MARTIN	PICOTA	464	3222 (3183 - 3262)	4% (2% - 6%)	5% (3% - 8%)
2017	SAN MARTIN	RIOJA	2457	3288 (3269 - 3306)	4% (3% - 5%)	6% (5% - 7%)
2017	SAN MARTIN	SAN MARTIN	5431	3167 (3152 - 3183)	10% (9% - 11%)	6% (6% - 7%)
2017	SAN MARTIN	TOCACHE	1393	3286 (3261 - 3311)	4% (3% - 6%)	5% (4% - 6%)
2018	SAN MARTIN	BELLAVISTA	564	3255 (3220 - 3291)	4% (3% - 6%)	5% (4% - 8%)
2018	SAN MARTIN	EL DORADO	470	3148 (3109 - 3187)	5% (3% - 7%)	7% (5% - 10%)
2018	SAN MARTIN	HUALLAGA	172	3300 (3243 - 3356)	1% (0% - 4%)	3% (1% - 7%)
2018	SAN MARTIN	LAMAS	656	3156 (3121 - 3191)	7% (5% - 9%)	9% (7% - 11%)
2018	SAN MARTIN	MARISCAL CACERES	924	3266 (3236 - 3295)	4% (3% - 5%)	6% (5% - 8%)
2018	SAN MARTIN	MOYOBAMBA	2459	3260 (3241 - 3279)	5% (4% - 6%)	5% (4% - 6%)
2018	SAN MARTIN	PICOTA	417	3229 (3188 - 3269)	4% (2% - 6%)	3% (2% - 5%)
2018	SAN MARTIN	RIOJA	2213	3287 (3267 - 3307)	5% (4% - 6%)	7% (6% - 8%)
2018	SAN MARTIN	SAN MARTIN	5192	3190 (3175 - 3205)	9% (8% - 10%)	6% (5% - 7%)
2018	SAN MARTIN	TOCACHE	1350	3295 (3270 - 3320)	4% (3% - 5%)	6% (5% - 7%)
2019	SAN MARTIN	BELLAVISTA	521	3245 (3203 - 3288)	4% (3% - 6%)	8% (6% - 11%)
2019	SAN MARTIN	EL DORADO	427	3128 (3088 - 3168)	6% (4% - 9%)	7% (5% - 10%)

2019	SAN MARTIN	HUALLAGA	171	3281 (3215 - 3347)	4% (2% - 8%)	6% (3% - 10%)
2019	SAN MARTIN	LAMAS	710	3144 (3113 - 3176)	5% (3% - 7%)	9% (7% - 12%)
2019	SAN MARTIN	MARISCAL CACERES	1003	3315 (3288 - 3343)	2% (2% - 4%)	4% (3% - 6%)
2019	SAN MARTIN	MOYOBAMBA	2506	3262 (3243 - 3281)	5% (4% - 6%)	4% (3% - 5%)
2019	SAN MARTIN	PICOTA	392	3263 (3220 - 3305)	3% (2% - 5%)	4% (2% - 6%)
2019	SAN MARTIN	RIOJA	2173	3270 (3250 - 3290)	4% (3% - 5%)	6% (5% - 8%)
2019	SAN MARTIN	SAN MARTIN	5481	3183 (3168 - 3198)	10% (9% - 10%)	7% (6% - 7%)
2019	SAN MARTIN	TOCACHE	1373	3294 (3271 - 3317)	4% (3% - 5%)	4% (3% - 5%)
2012	TACNA	TACNA	449	3427 (3372 - 3481)	6% (4% - 9%)	2% (1% - 4%)
2013	TACNA	TACNA	3505	3501 (3482 - 3519)	4% (4% - 5%)	3% (2% - 3%)
2014	TACNA	TACNA	4252	3533 (3517 - 3549)	3% (3% - 4%)	2% (1% - 2%)
2015	TACNA	TACNA	5084	3489 (3474 - 3504)	5% (4% - 5%)	2% (2% - 3%)
2016	TACNA	TACNA	5023	3510 (3495 - 3524)	3% (3% - 4%)	2% (2% - 2%)
2017	TACNA	TACNA	5077	3500 (3485 - 3515)	4% (3% - 4%)	1% (1% - 2%)
2018	TACNA	TACNA	5006	3518 (3503 - 3533)	4% (3% - 4%)	2% (1% - 2%)
2019	TACNA	TACNA	4846	3498 (3483 - 3514)	4% (3% - 5%)	2% (1% - 2%)
2012	TUMBES	TUMBES	99	3392 (3291 - 3492)	3% (1% - 9%)	3% (1% - 9%)
2013	TUMBES	CONTRALMIRANTE VILLAR	94	3268 (3178 - 3358)	4% (1% - 11%)	9% (4% - 16%)
2013	TUMBES	TUMBES	2996	3279 (3259 - 3298)	6% (6% - 7%)	5% (4% - 6%)
2013	TUMBES	ZARUMILLA	278	3346 (3298 - 3394)	2% (1% - 4%)	3% (1% - 5%)
2014	TUMBES	CONTRALMIRANTE VILLAR	107	3215 (3135 - 3294)	2% (0% - 7%)	8% (4% - 15%)
2014	TUMBES	TUMBES	3500	3269 (3252 - 3287)	7% (6% - 7%)	5% (4% - 5%)
2014	TUMBES	ZARUMILLA	277	3304 (3254 - 3353)	4% (2% - 7%)	6% (3% - 9%)
2015	TUMBES	CONTRALMIRANTE VILLAR	121	3221 (3144 - 3298)	4% (1% - 9%)	9% (5% - 16%)
2015	TUMBES	TUMBES	3846	3257 (3239 - 3274)	7% (6% - 7%)	4% (4% - 5%)
2015	TUMBES	ZARUMILLA	264	3311 (3265 - 3358)	2% (1% - 4%)	3% (1% - 6%)
2016	TUMBES	CONTRALMIRANTE VILLAR	111	3191 (3120 - 3261)	3% (1% - 8%)	4% (1% - 9%)
2016	TUMBES	TUMBES	3796	3256 (3239 - 3273)	7% (6% - 8%)	4% (3% - 4%)
2016	TUMBES	ZARUMILLA	291	3296 (3250 - 3342)	2% (1% - 4%)	3% (2% - 6%)
2017	TUMBES	CONTRALMIRANTE VILLAR	105	3362 (3277 - 3448)	1% (0% - 5%)	3% (1% - 8%)

2017	TUMBES	TUMBES	3782	3258 (3241 - 3275)	7% (6% - 7%)	5% (4% - 5%)
2017	TUMBES	ZARUMILLA	303	3309 (3260 - 3357)	2% (1% - 5%)	4% (2% - 7%)
2018	TUMBES	CONTRALMIRANTE VILLAR	123	3357 (3275 - 3438)	2% (0% - 6%)	2% (1% - 7%)
2018	TUMBES	TUMBES	3738	3278 (3261 - 3295)	6% (5% - 7%)	4% (4% - 5%)
2018	TUMBES	ZARUMILLA	326	3240 (3195 - 3286)	4% (2% - 6%)	5% (3% - 8%)
2019	TUMBES	CONTRALMIRANTE VILLAR	125	3265 (3189 - 3341)	2% (0% - 6%)	4% (1% - 9%)
2019	TUMBES	TUMBES	3691	3282 (3265 - 3300)	7% (6% - 8%)	4% (4% - 5%)
2019	TUMBES	ZARUMILLA	280	3284 (3237 - 3332)	3% (1% - 6%)	4% (2% - 6%)
2012	UCAYALI	PADRE ABAD	176	3248 (3179 - 3318)	6% (3% - 11%)	7% (4% - 12%)
2013	UCAYALI	CORONEL PORTILLO	756	3192 (3153 - 3232)	8% (6% - 10%)	10% (8% - 12%)
2013	UCAYALI	PADRE ABAD	542	3291 (3252 - 3330)	5% (3% - 7%)	6% (4% - 8%)
2014	UCAYALI	ATALAYA	251	3122 (3068 - 3177)	8% (5% - 12%)	10% (7% - 15%)
2014	UCAYALI	CORONEL PORTILLO	6582	3200 (3187 - 3213)	8% (7% - 8%)	9% (8% - 9%)
2014	UCAYALI	PADRE ABAD	667	3294 (3261 - 3328)	4% (2% - 5%)	7% (5% - 9%)
2015	UCAYALI	ATALAYA	554	3044 (3006 - 3082)	9% (7% - 12%)	11% (9% - 14%)
2015	UCAYALI	CORONEL PORTILLO	8947	3184 (3173 - 3195)	8% (7% - 8%)	9% (9% - 10%)
2015	UCAYALI	PADRE ABAD	556	3308 (3271 - 3345)	3% (1% - 4%)	4% (3% - 6%)
2016	UCAYALI	ATALAYA	629	3108 (3073 - 3144)	8% (6% - 10%)	13% (10% - 15%)
2016	UCAYALI	CORONEL PORTILLO	9777	3178 (3167 - 3188)	8% (7% - 9%)	9% (9% - 10%)
2016	UCAYALI	PADRE ABAD	812	3338 (3308 - 3368)	2% (1% - 3%)	4% (3% - 6%)
2017	UCAYALI	ATALAYA	744	3097 (3064 - 3131)	8% (6% - 10%)	11% (9% - 14%)
2017	UCAYALI	CORONEL PORTILLO	10557	3193 (3183 - 3203)	8% (7% - 8%)	10% (9% - 10%)
2017	UCAYALI	PADRE ABAD	906	3328 (3300 - 3356)	2% (1% - 3%)	4% (3% - 6%)
2017	UCAYALI	PURUS	63	3041 (2948 - 3134)	6% (2% - 15%)	8% (3% - 18%)
2018	UCAYALI	ATALAYA	743	3041 (3009 - 3073)	8% (6% - 11%)	13% (11% - 16%)
2018	UCAYALI	CORONEL PORTILLO	10217	3188 (3177 - 3198)	7% (7% - 8%)	9% (8% - 9%)
2018	UCAYALI	PADRE ABAD	980	3306 (3279 - 3333)	3% (2% - 4%)	4% (3% - 6%)
2018	UCAYALI	PURUS	45	3099 (2975 - 3223)	7% (1% - 18%)	9% (2% - 21%)
2019	UCAYALI	ATALAYA	838	3106 (3076 - 3137)	6% (5% - 8%)	10% (8% - 12%)
2019	UCAYALI	CORONEL PORTILLO	10774	3175 (3165 - 3184)	8% (8% - 9%)	9% (8% - 10%)

2019	UCAYALI	PADRE ABAD	985	3296 (3268 - 3324)	3% (2% - 4%)	5% (3% - 6%)
2019	UCAYALI	PURUS	59	3140 (3026 - 3253)	3% (0% - 12%)	10% (4% - 21%)

Provinces (i.e., rows in the table) with no prevalence results are because there were no cases of low birth weight or small for gestational age.

Supplementary Figure 2. Regional time trends of mean birthweight in Peru between 2012 and 2019, overall and by sex (point estimates are shown along with the 95% confidence intervals).

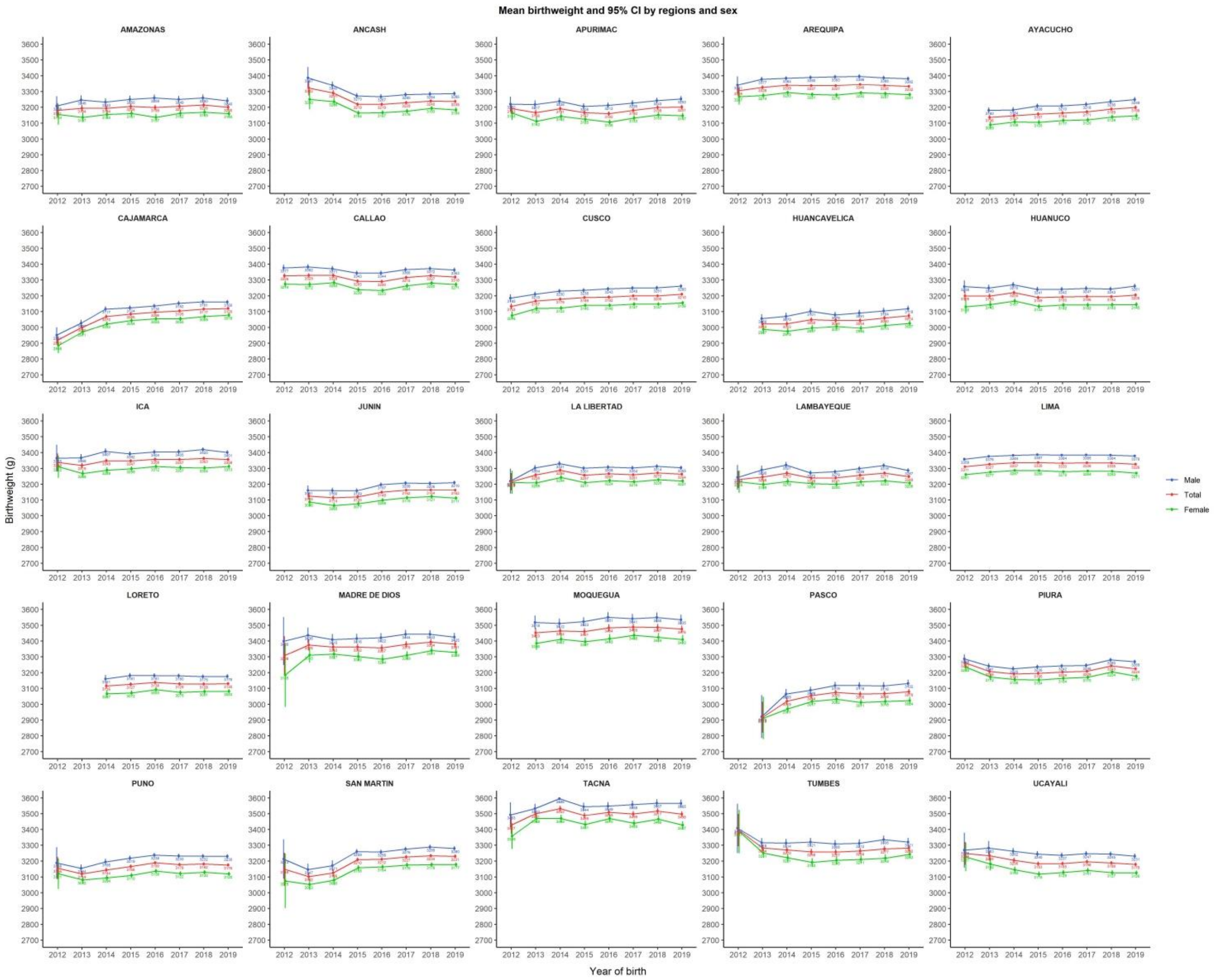

Supplementary Figure 3. Regional time trends of low birthweight prevalence in Peru between 2012 and 2019, overall and by sex (point estimates are shown along with the 95% confidence intervals).

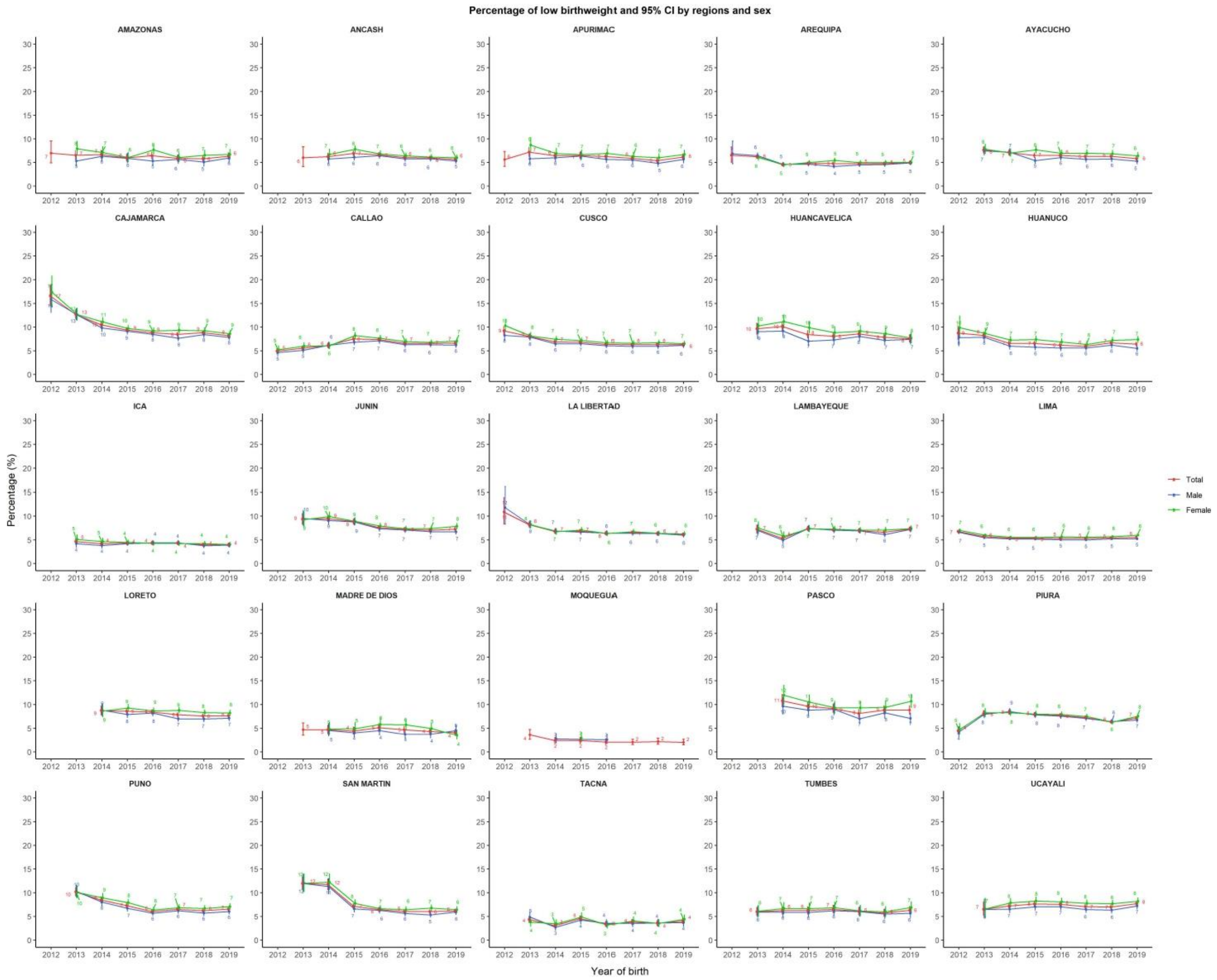

Supplementary Figure 4. Regional time trends of small for gestational age prevalence in Peru between 2012 and 2019, overall and by sex (point estimates are shown along with the 95% confidence intervals).

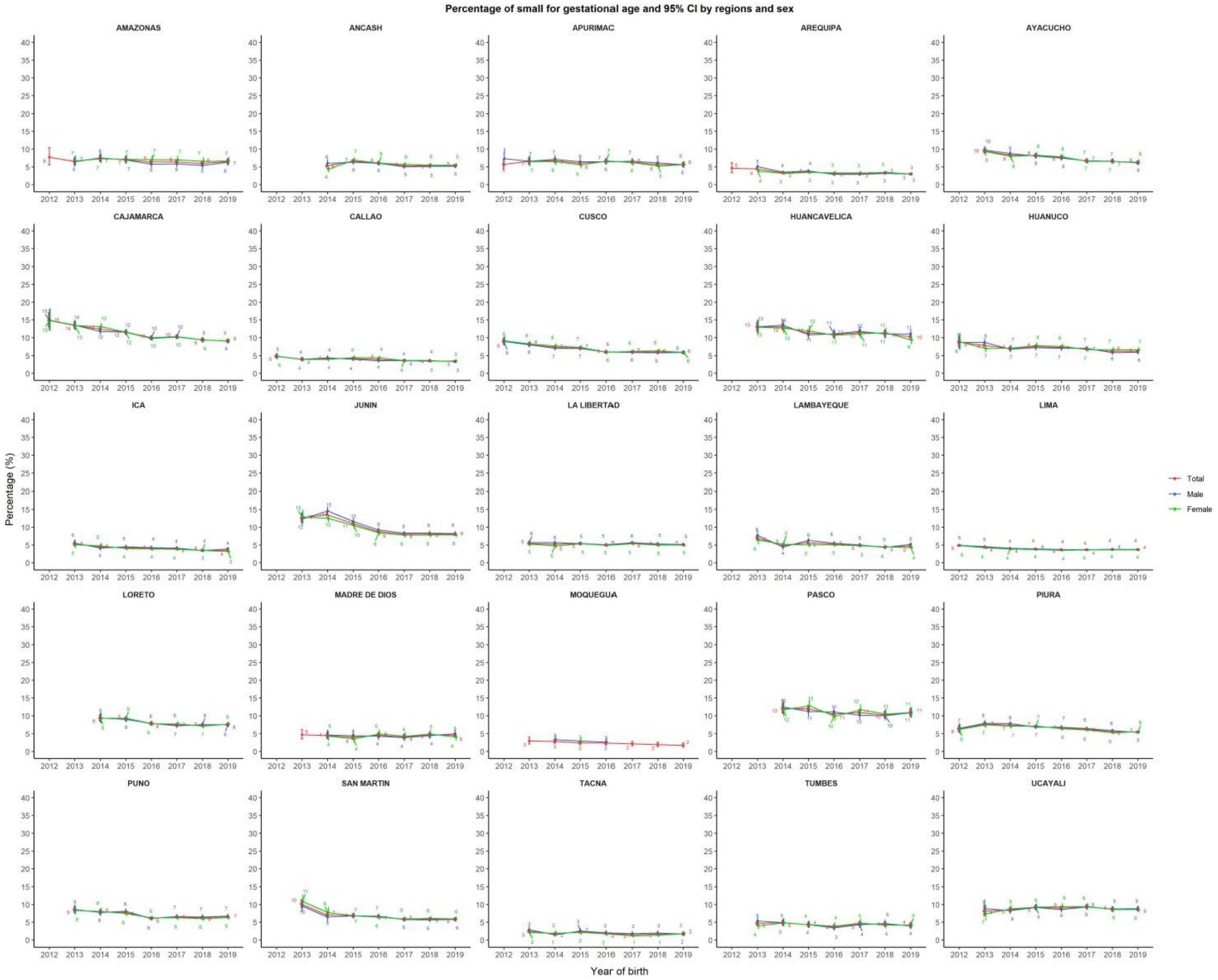

Supplementary Figure 5. Scatterplots of mean birthweight, low birthweight prevalence and small for gestational age prevalence with human development index (HDI), altitude above sea level, poverty, and rurality.

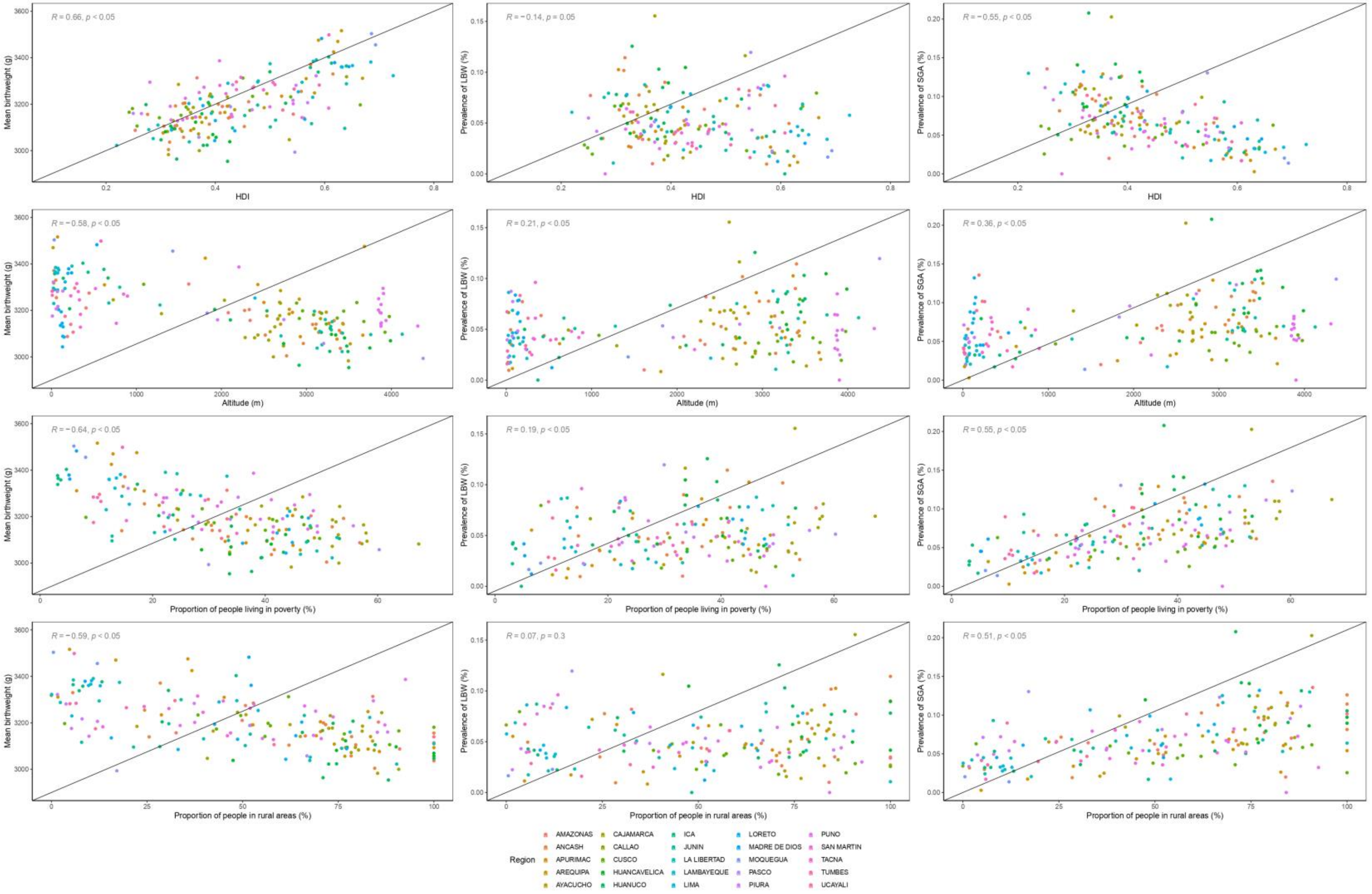

Each dot represents one province, and the colours show the region to which they belong. The Pearson correlation coefficient is presented in each plot. LBW: low birth weight; SGA: small for gestational age.

Supplementary Figure 6. Geographic profiles of mean birthweight, low birthweight prevalence and small for gestational age prevalence in Peru between 2012 and 2019.

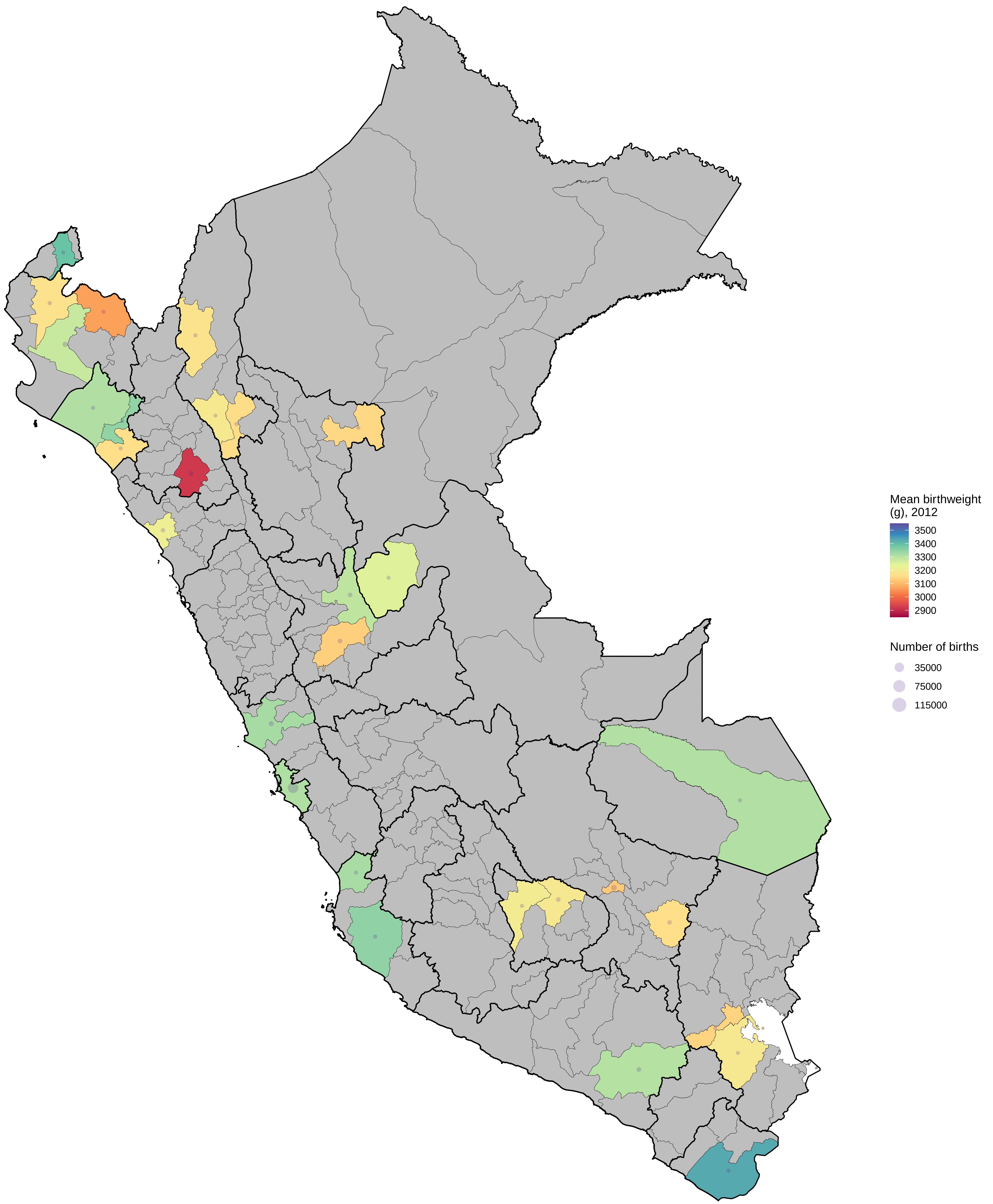

Mean birthweight (g), 2012
3500
3400
3300
3200
3100
3000
2900
Number of births
35000
75000
115000

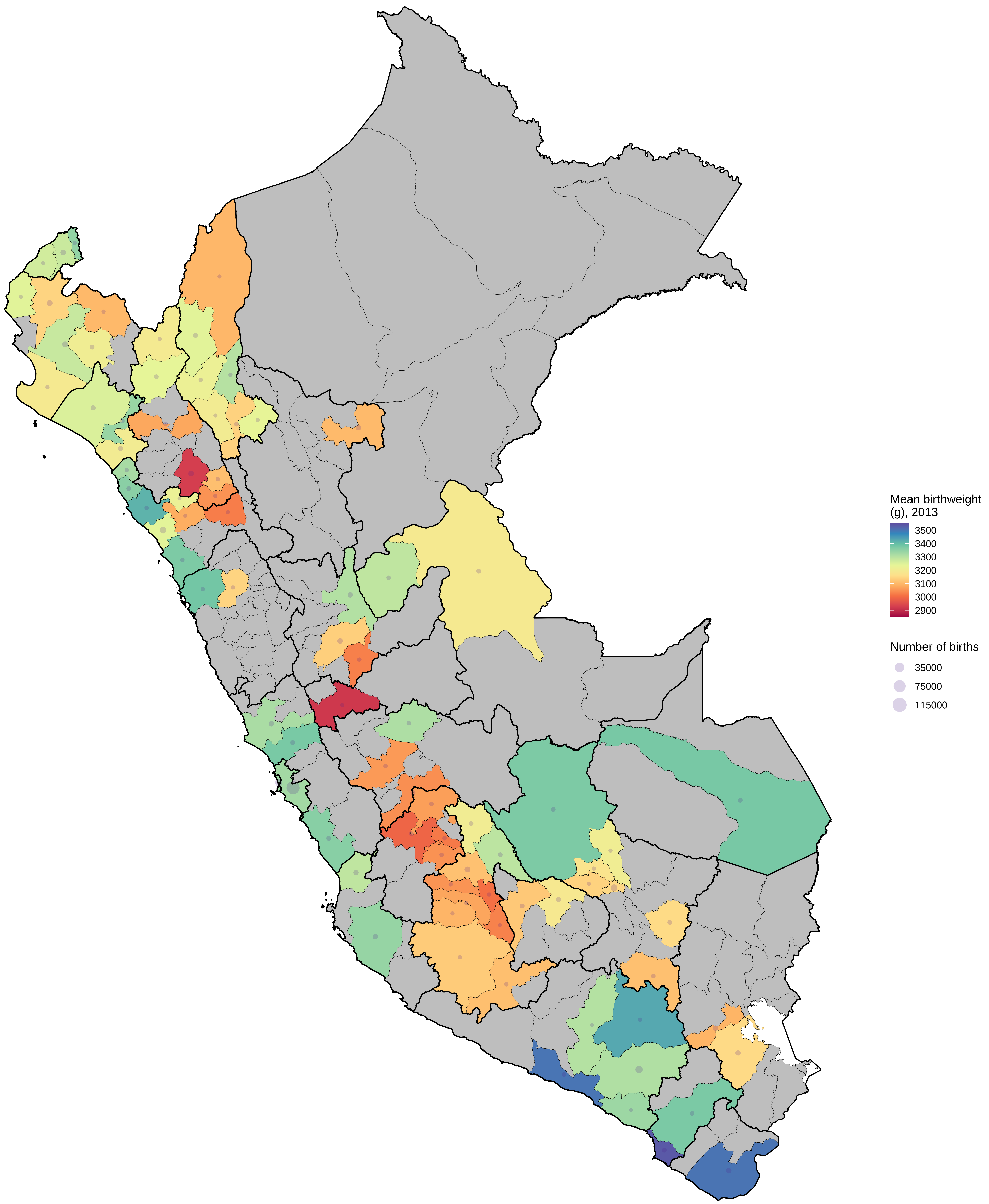

Mean birthweight
(g), 2013
3500
3400
3300
3200
3100
3000
2900
Number of births
35000
75000
115000

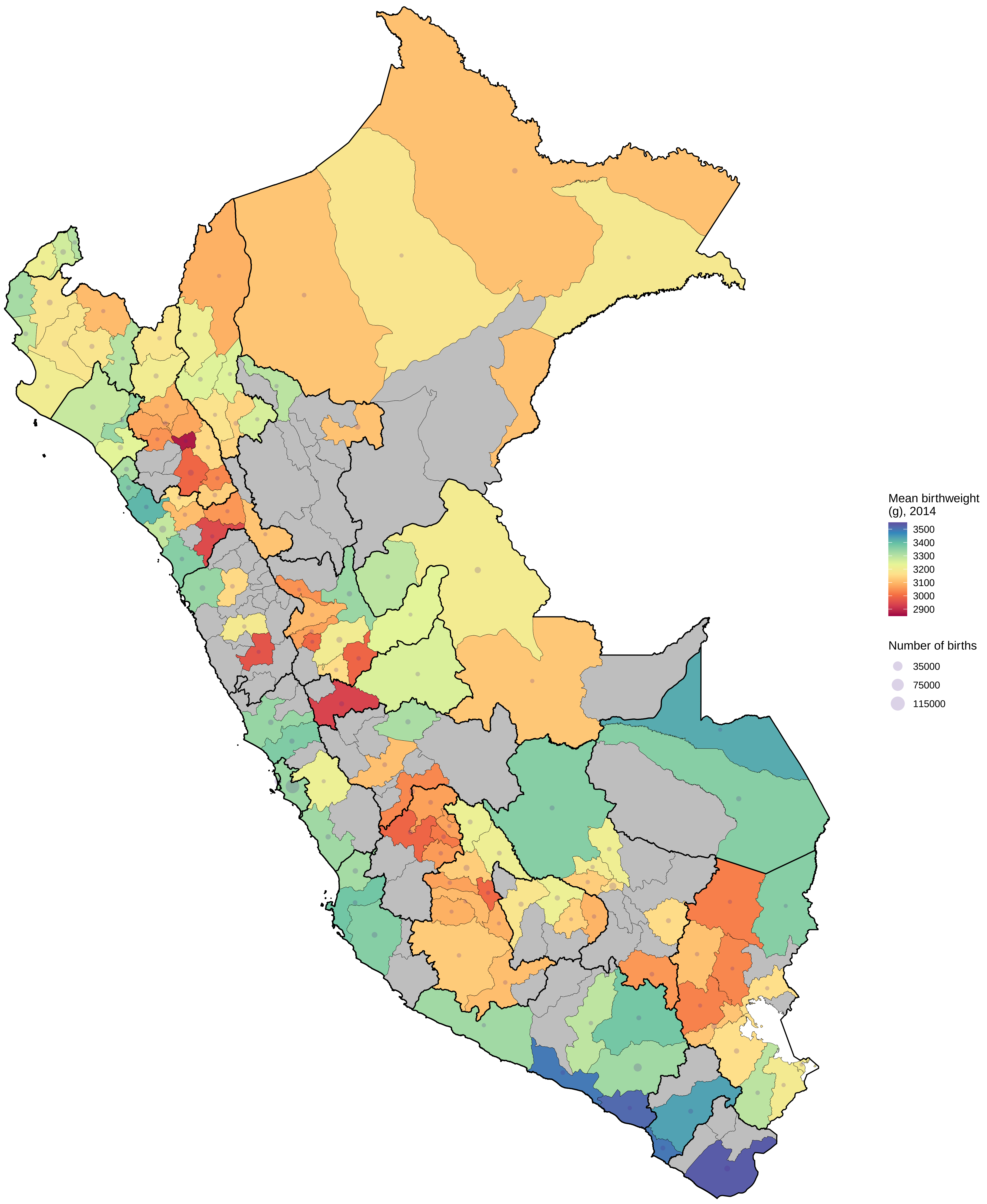
Mean birthweight
(g), 2014
3500
3400
3300
3200
3100
3000
2900
Number of births
35000
75000
115000

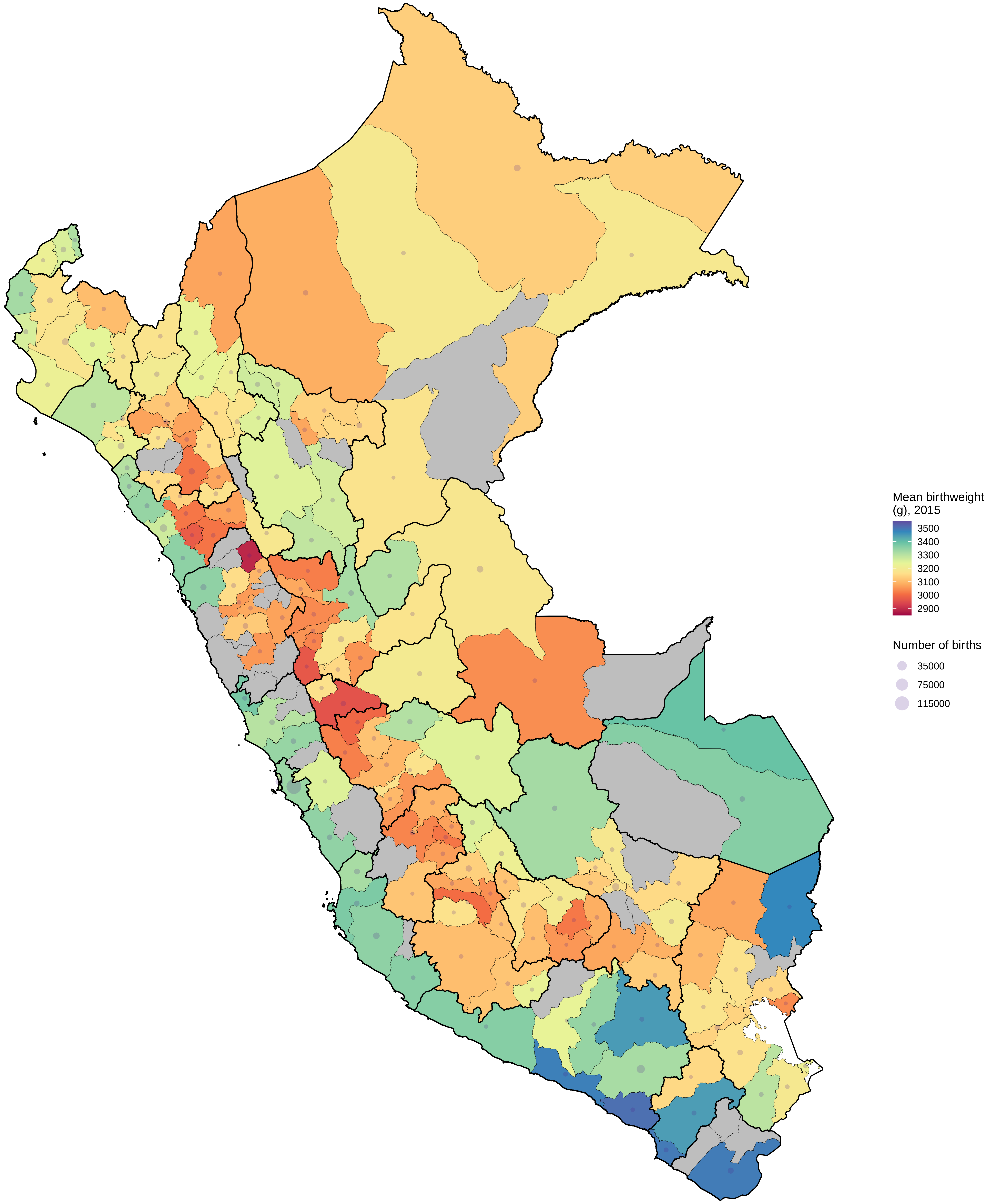

Mean birthweight
(g), 2015
3500
3400
3300
3200
3100
3000
2900
Number of births
35000
75000
115000

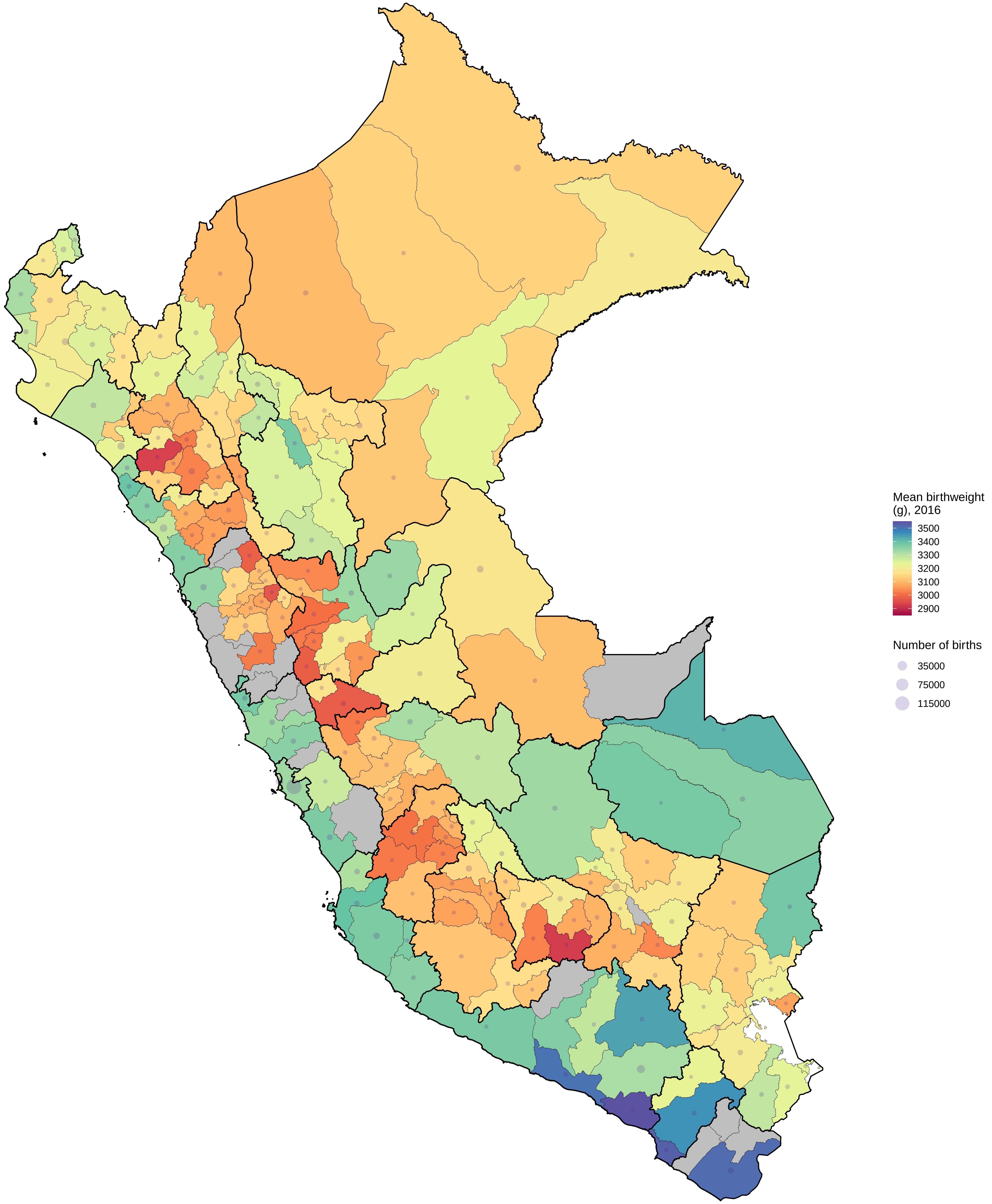

Mean birthweight (g), 2016
3500
3400
3300
3200
3100
3000
2900
Number of births
35000
75000
115000

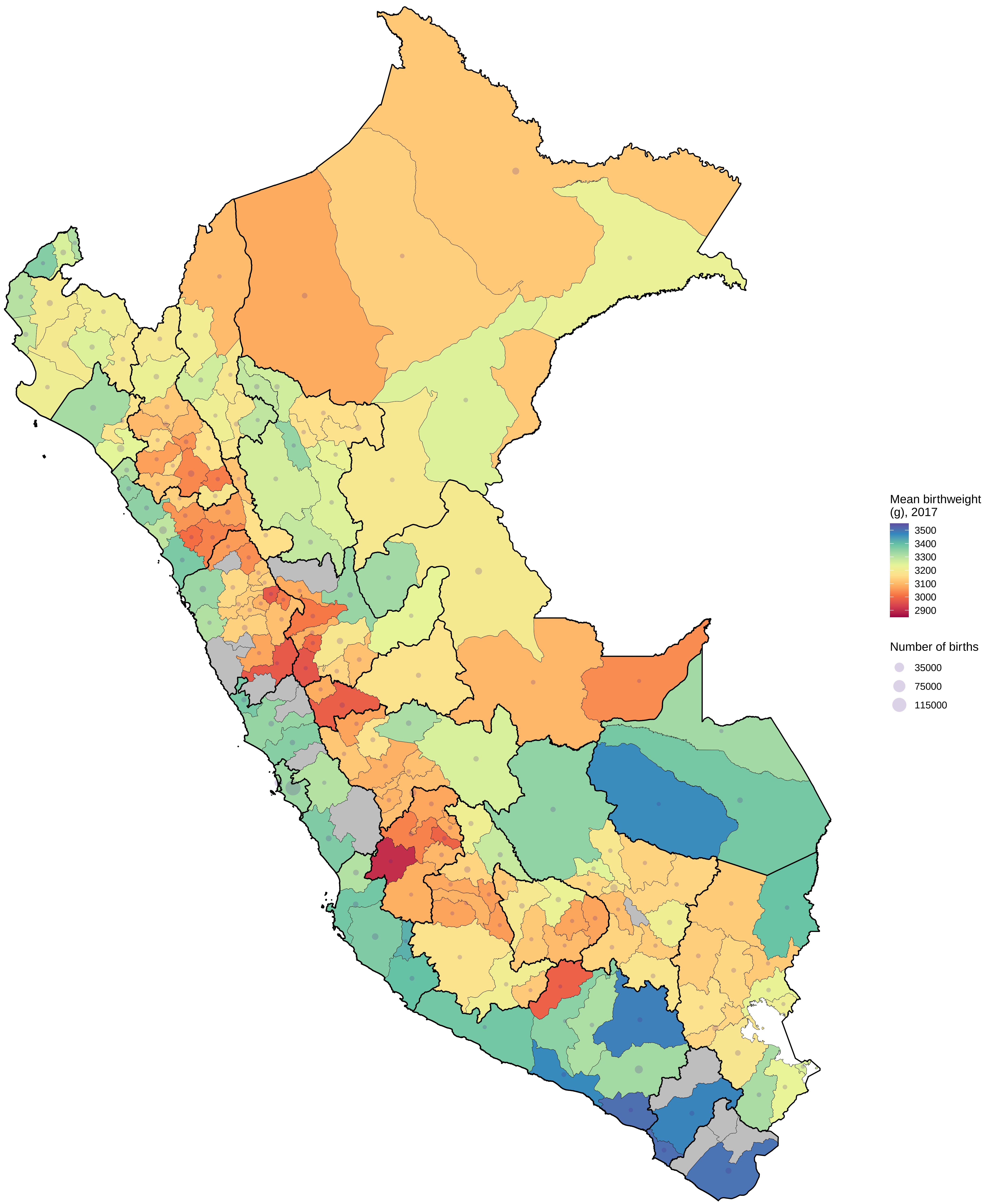

Mean birthweight
(g), 2017
3500
3400
3300
3200
3100
3000
2900
Number of births
35000
75000
115000

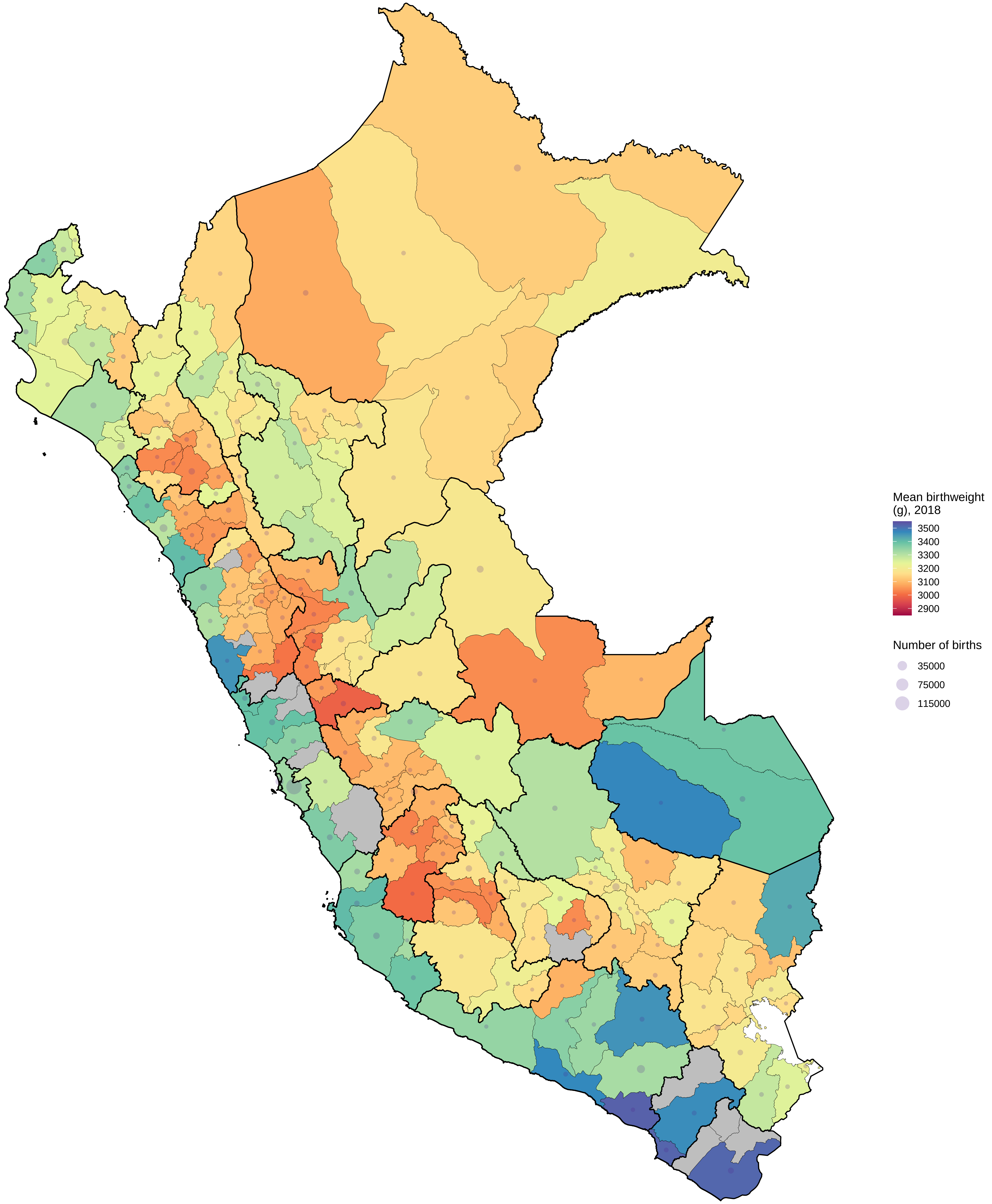
Mean birthweight
(g), 2018
3500
3400
3300
3200
3100
3000
2900
Number of births
35000
75000
115000

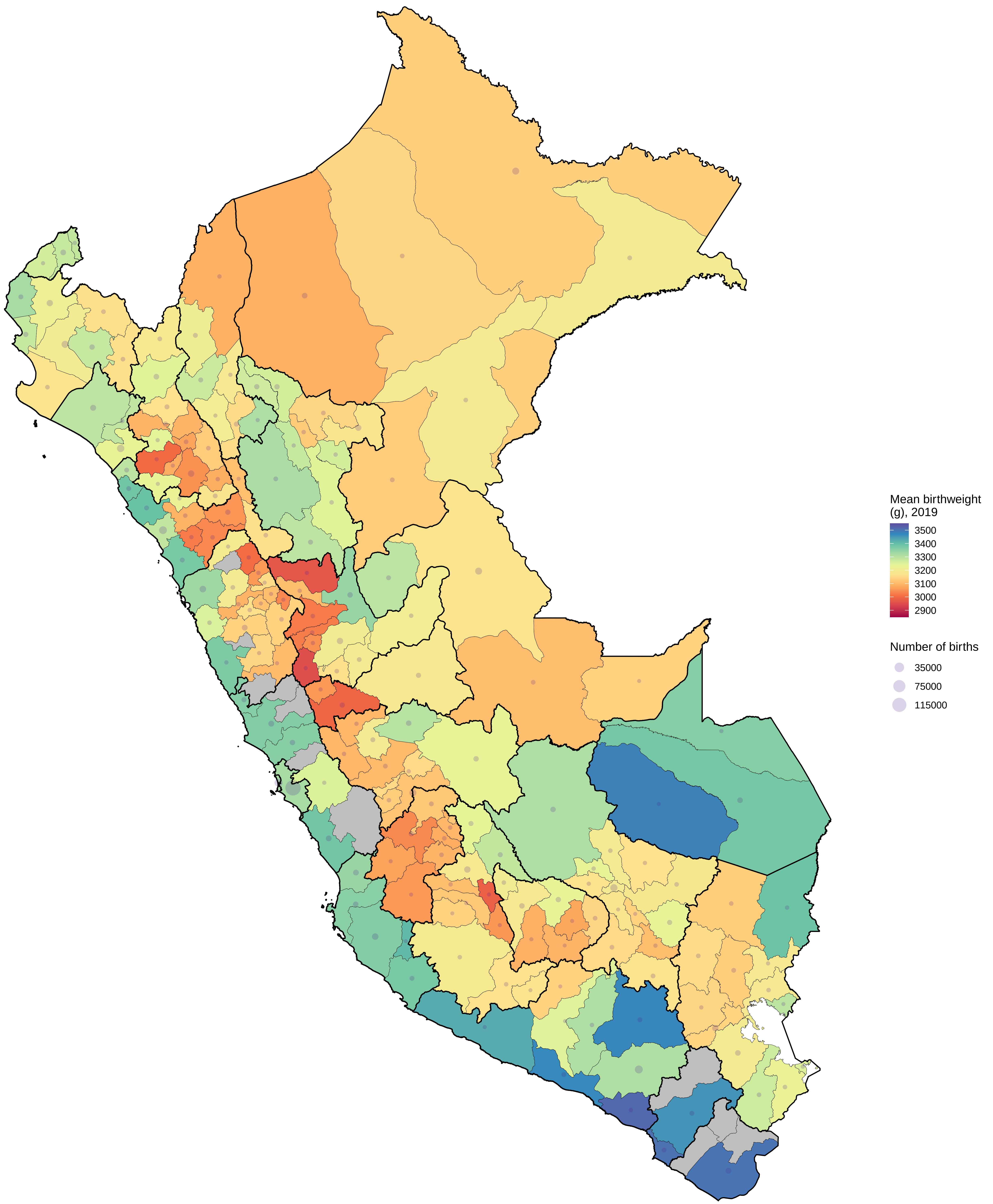

Mean birthweight
(g), 2019
3500
3400
3300
3200
3100
3000
2900
Number of births
35000
75000
115000

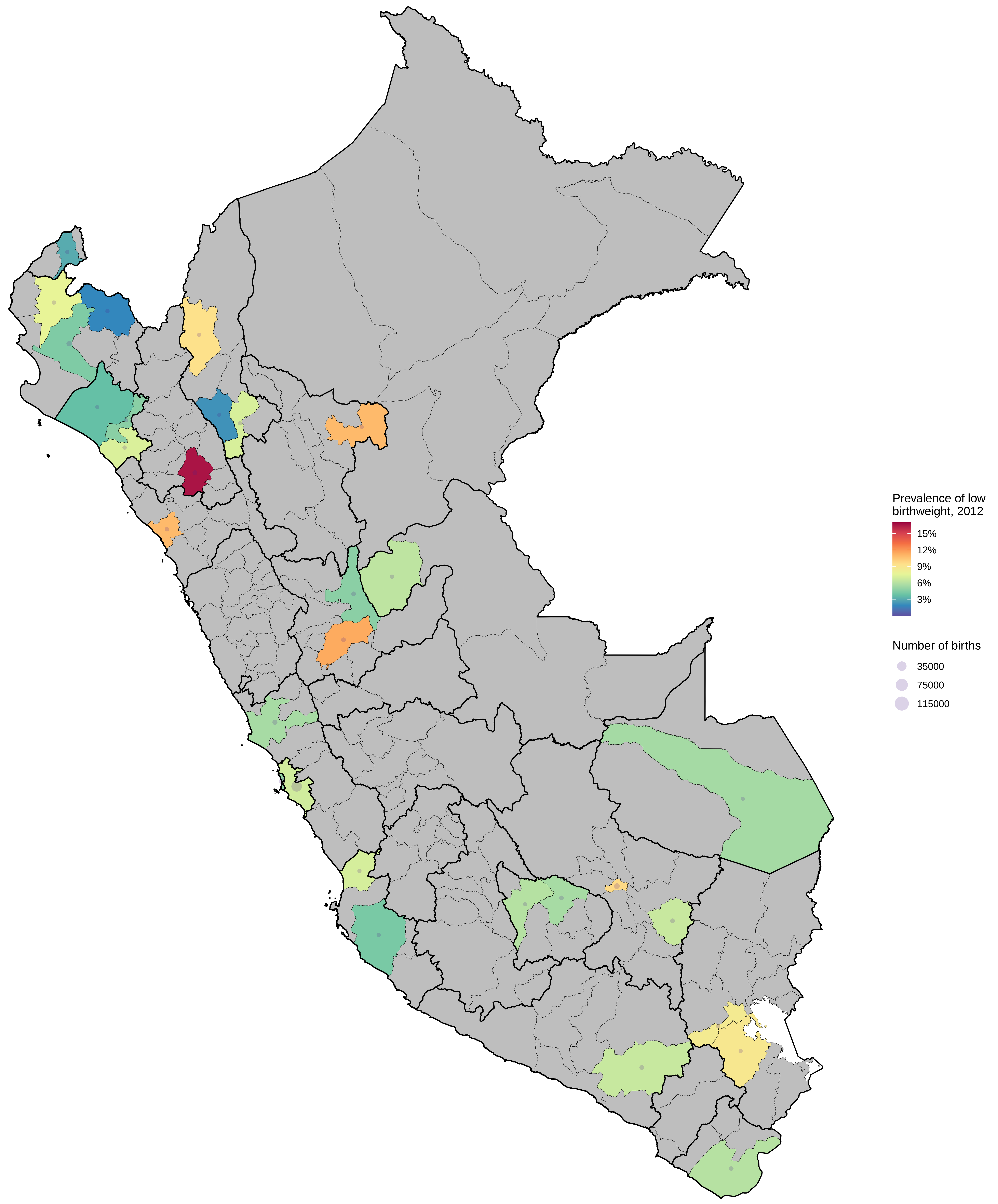

Prevalence of low birthweight, 2012
15%
12%
9%
6%
3%
Number of births
35000
75000
115000

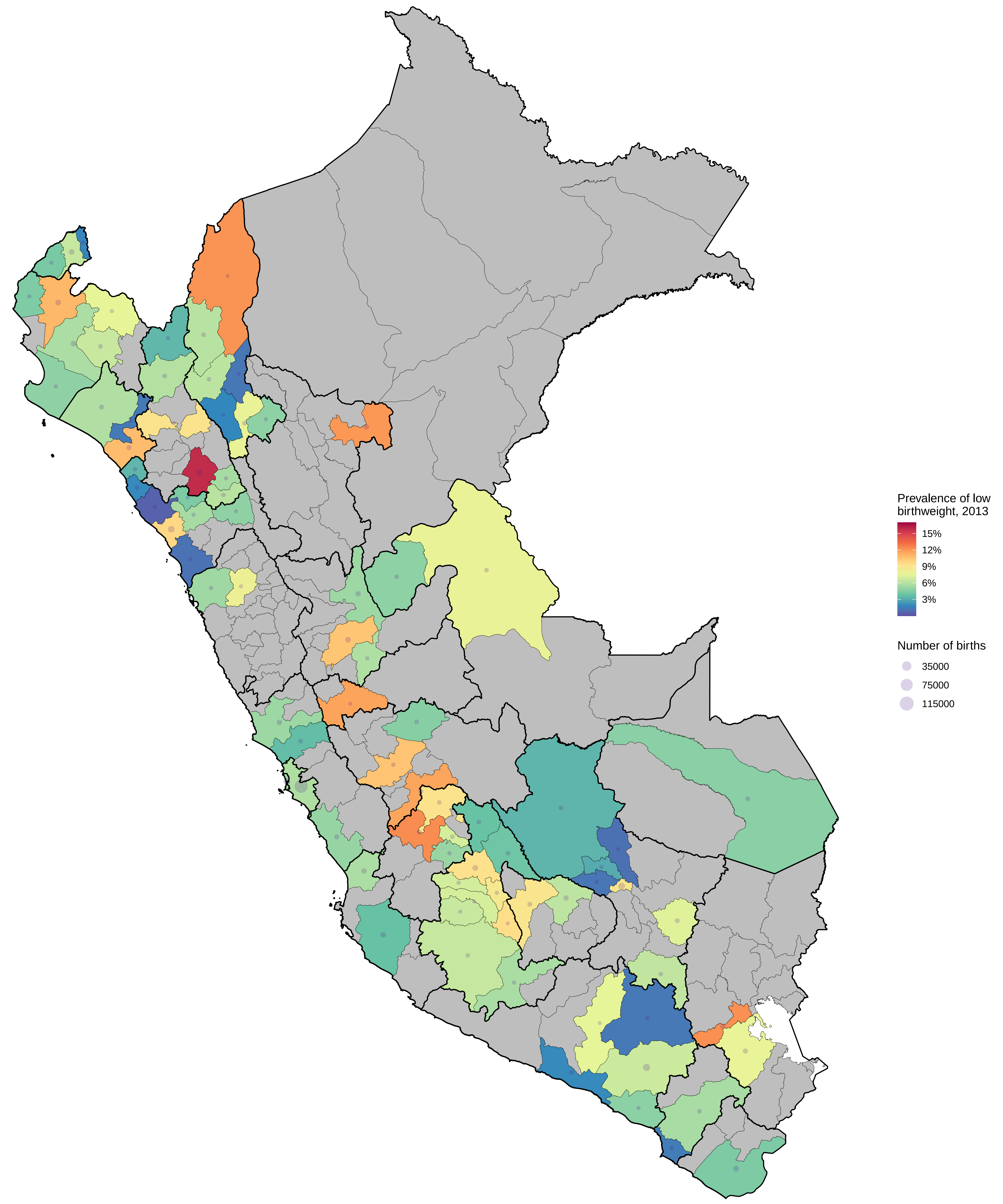

Prevalence of low birthweight, 2013
15%
12%
9%
6%
3%
Number of births
35000
75000
115000

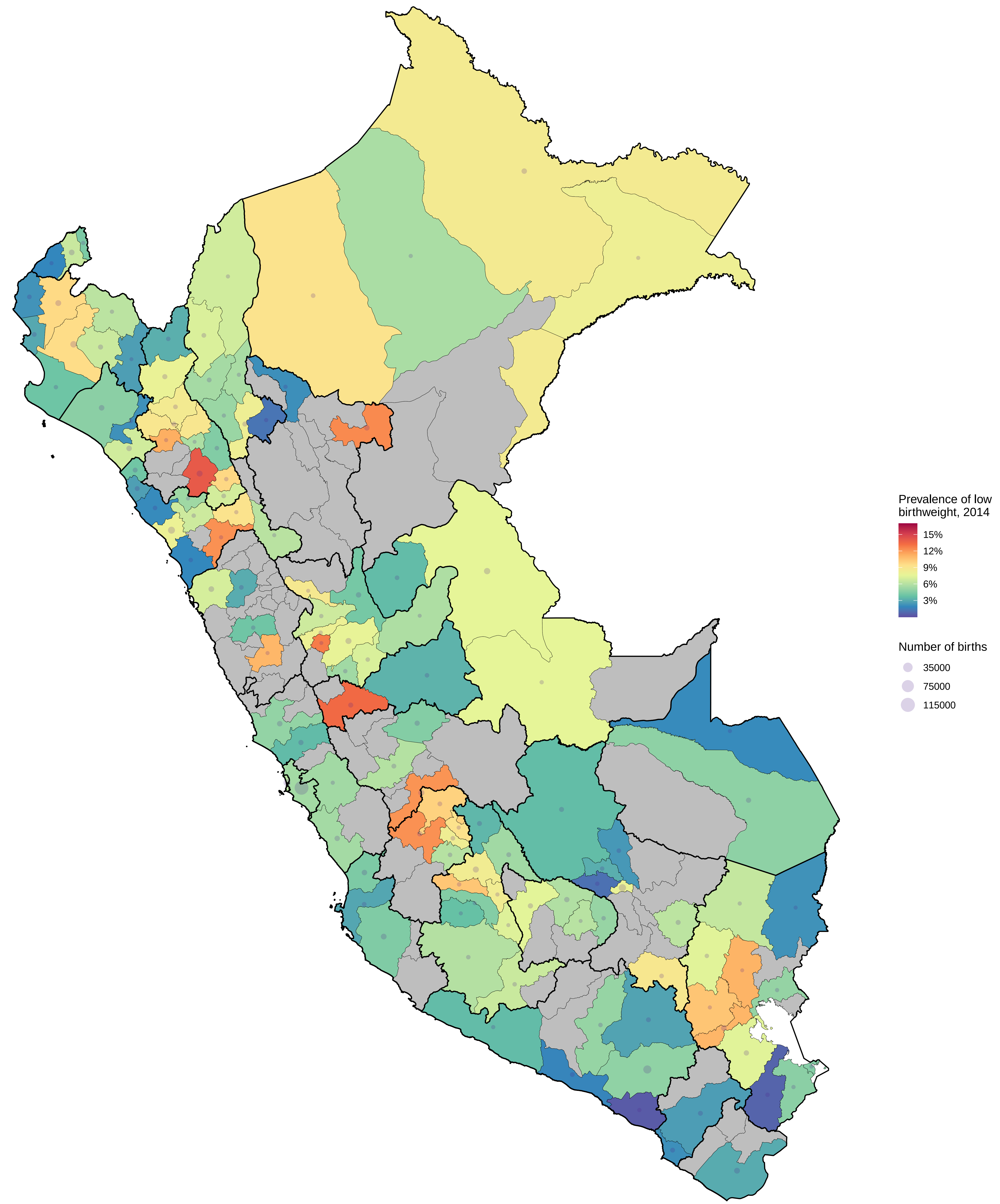

Prevalence of low birthweight, 2014
15%
12%
9%
6%
3%
Number of births
35000
75000
115000

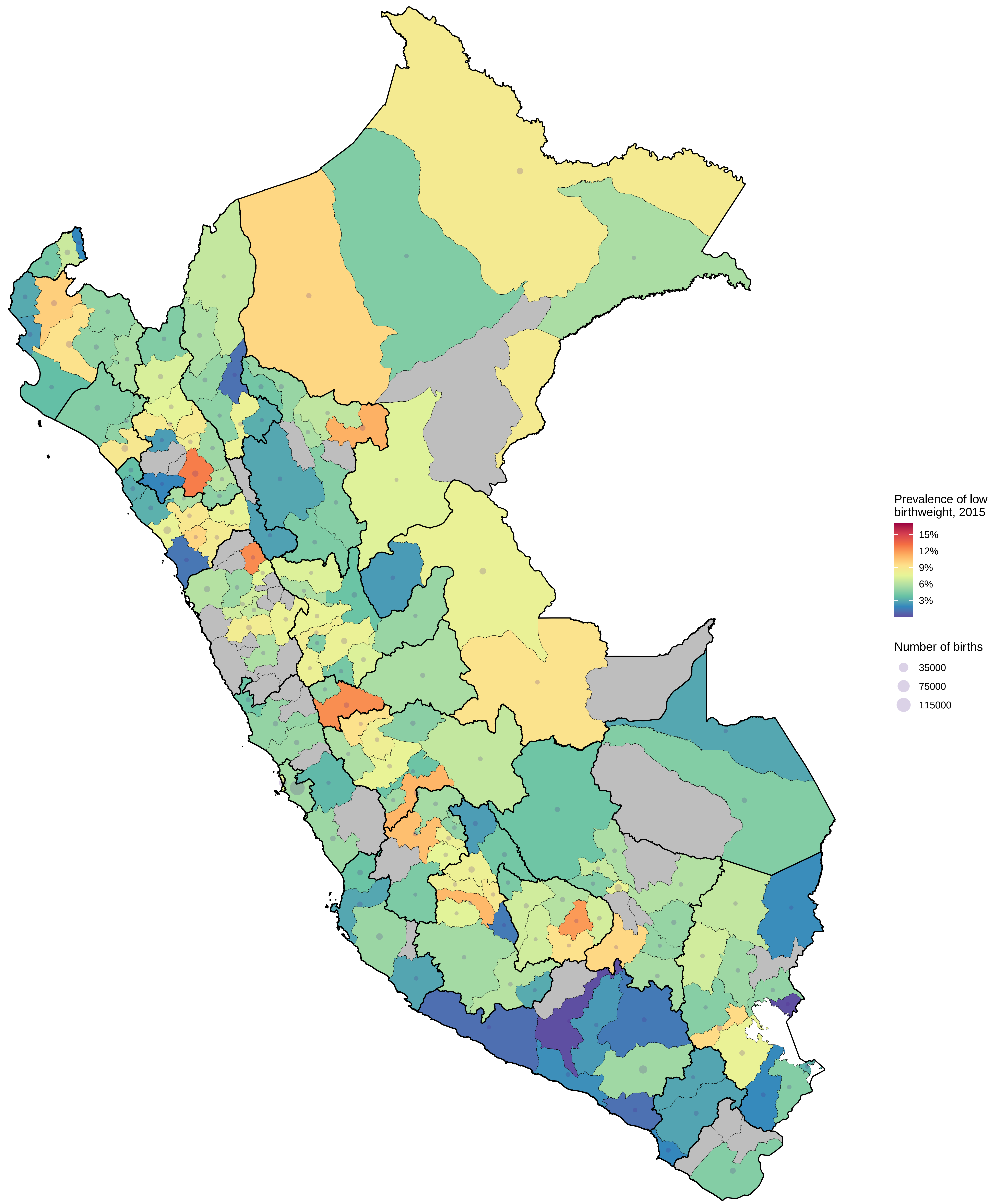

Prevalence of low birthweight, 2015
15%
12%
9%
6%
3%
Number of births
35000
75000
115000

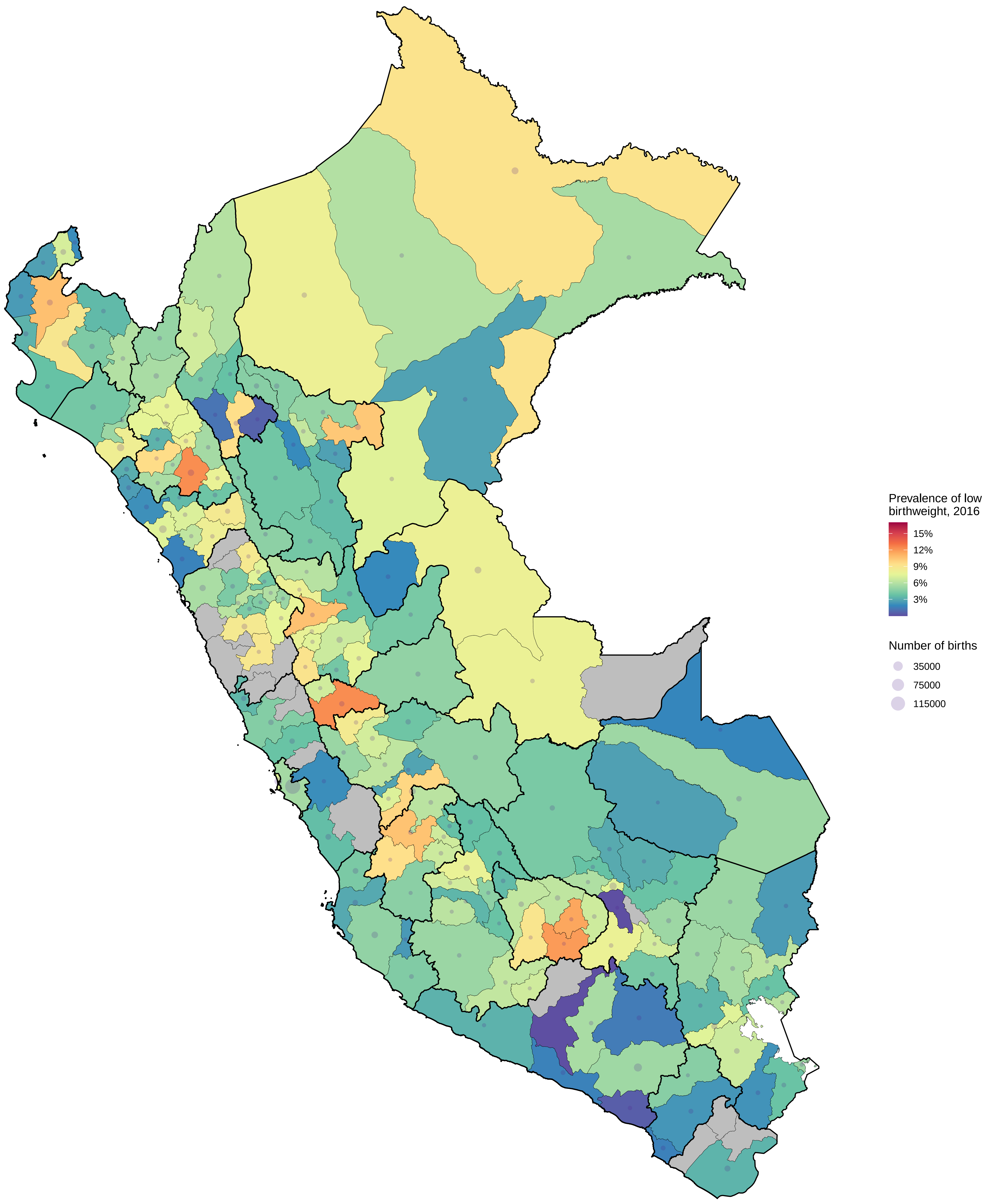

Prevalence of low birthweight, 2016
15%
12%
9%
6%
3%
Number of births
35000
75000
115000

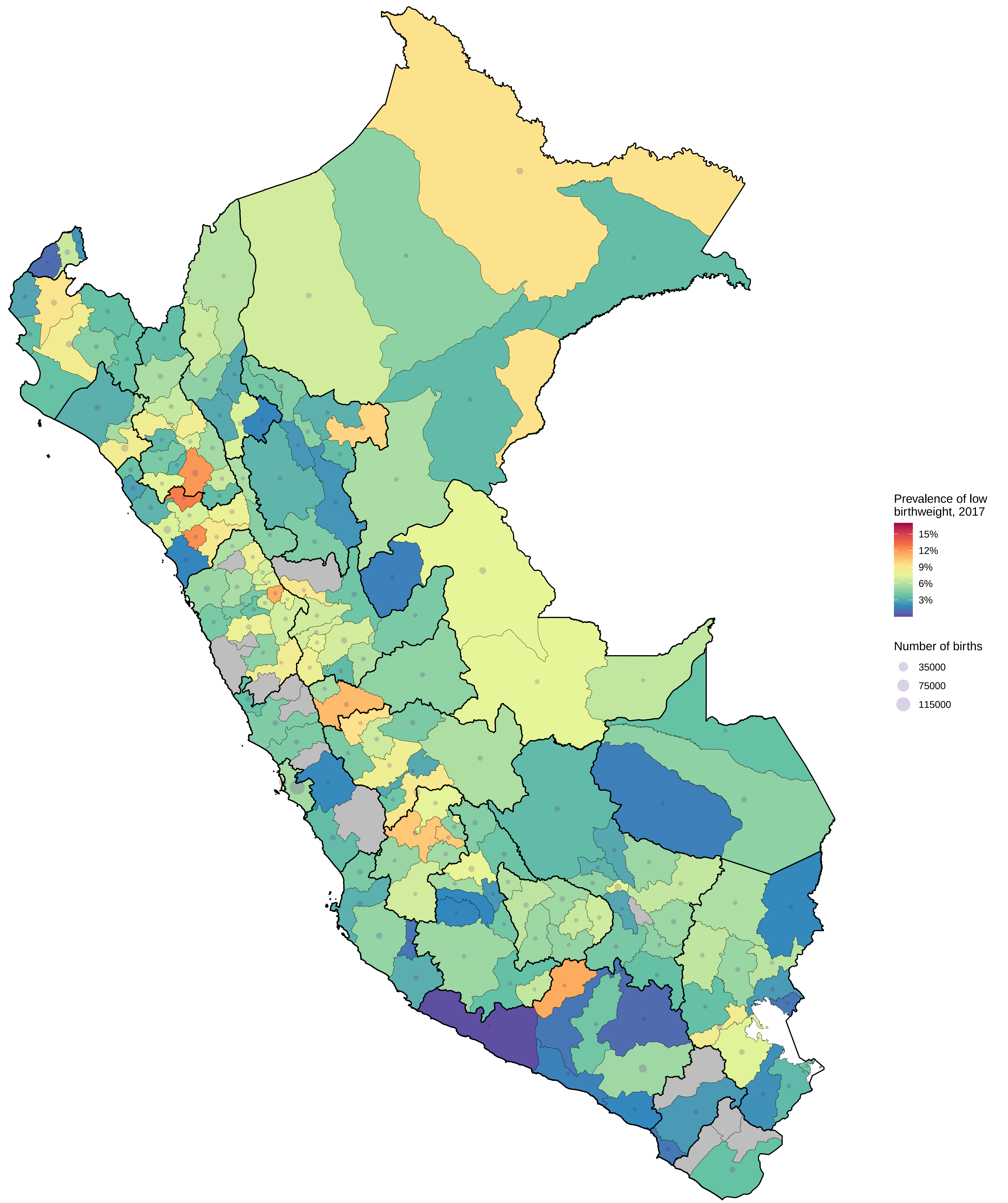
Prevalence of low birthweight, 2017
15%
12%
9%
6%
3%
Number of births
35000
75000
115000

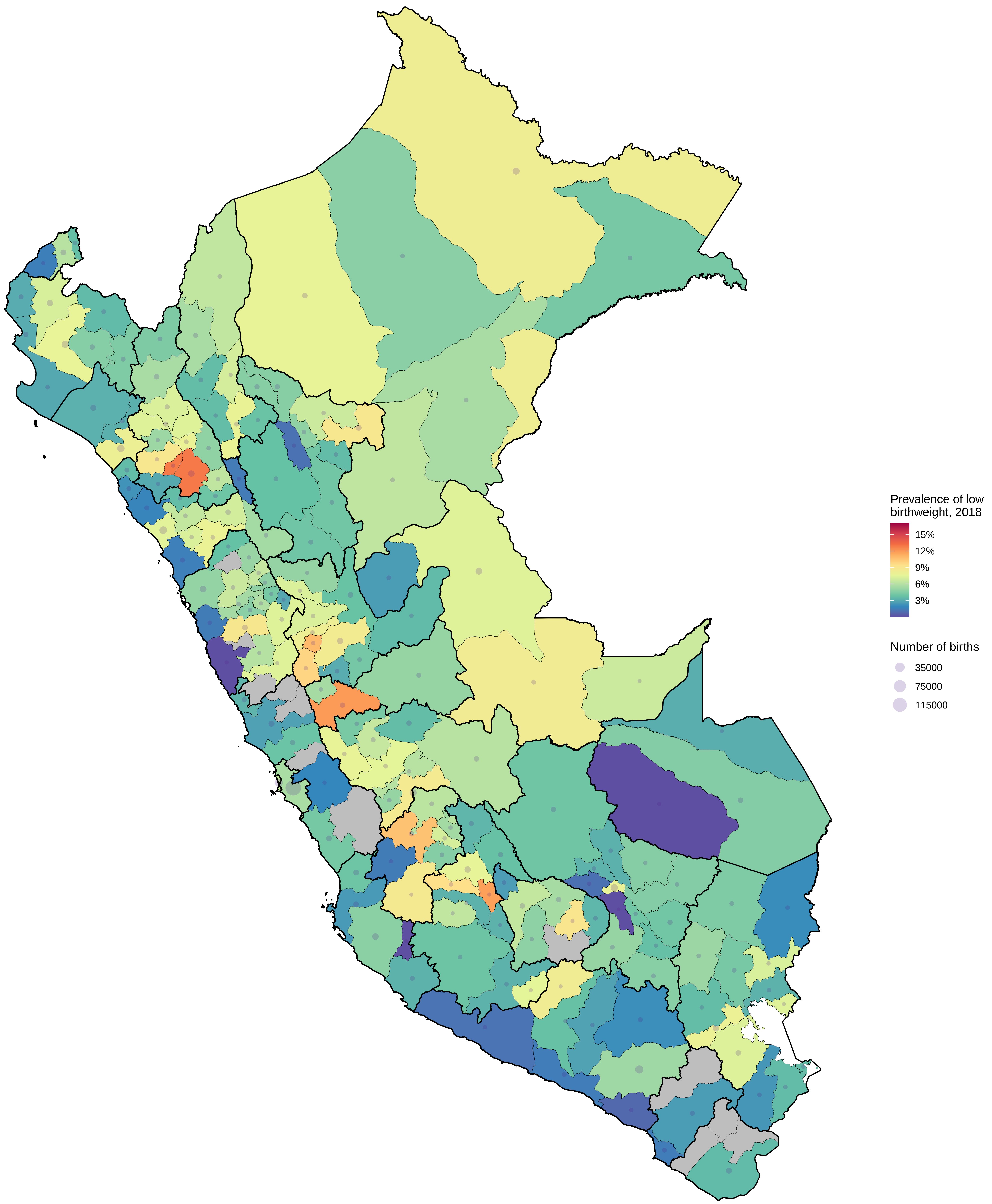

Prevalence of low
birthweight, 2018
15%
12%
9%
6%
3%
Number of births
35000
75000
115000

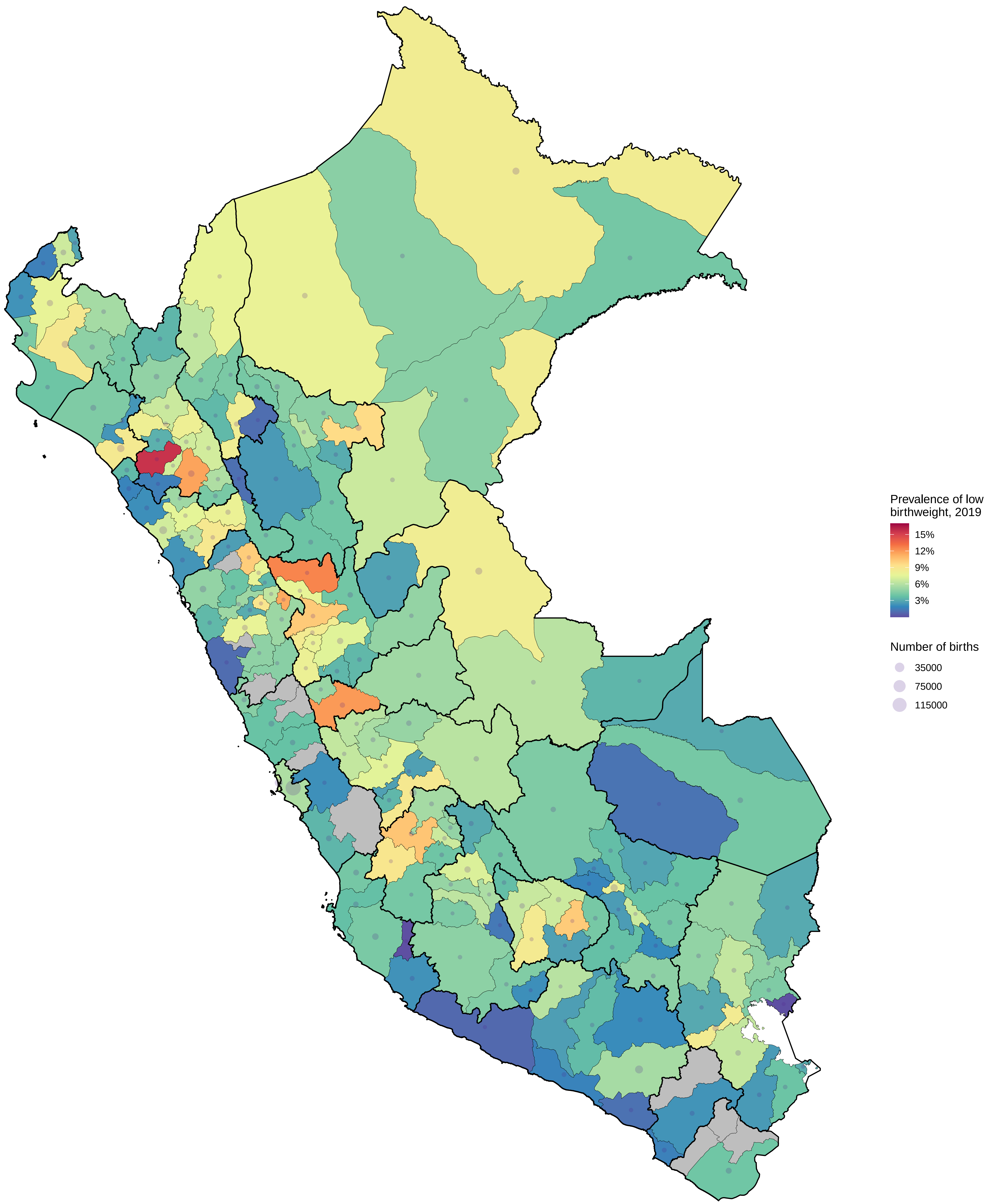

Prevalence of low birthweight, 2019
15%
12%
9%
6%
3%
Number of births
35000
75000
115000

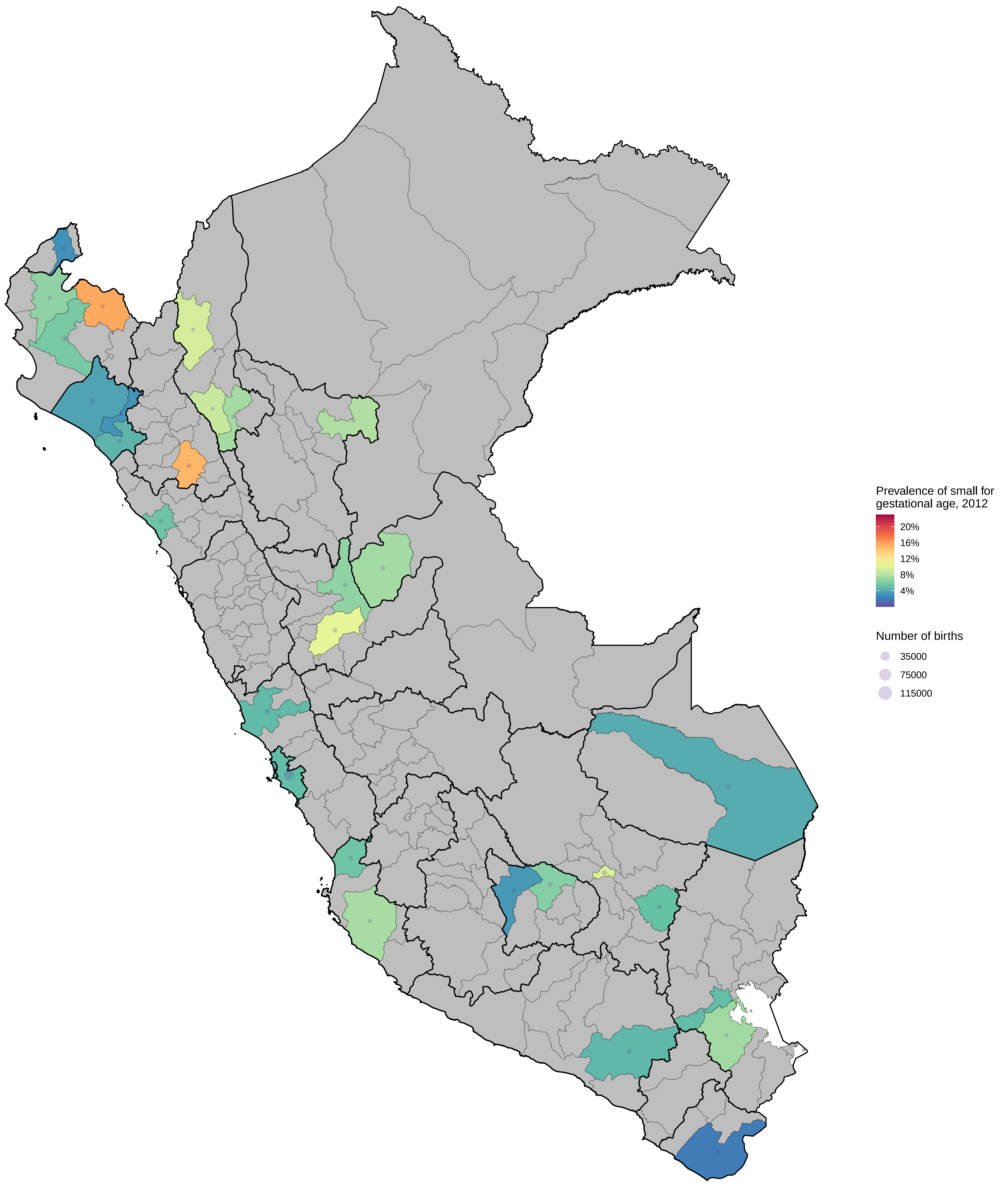

Prevalence of small for gestational age, 2012
20%
16%
12%
8%
4%
Number of births
35000
75000
115000

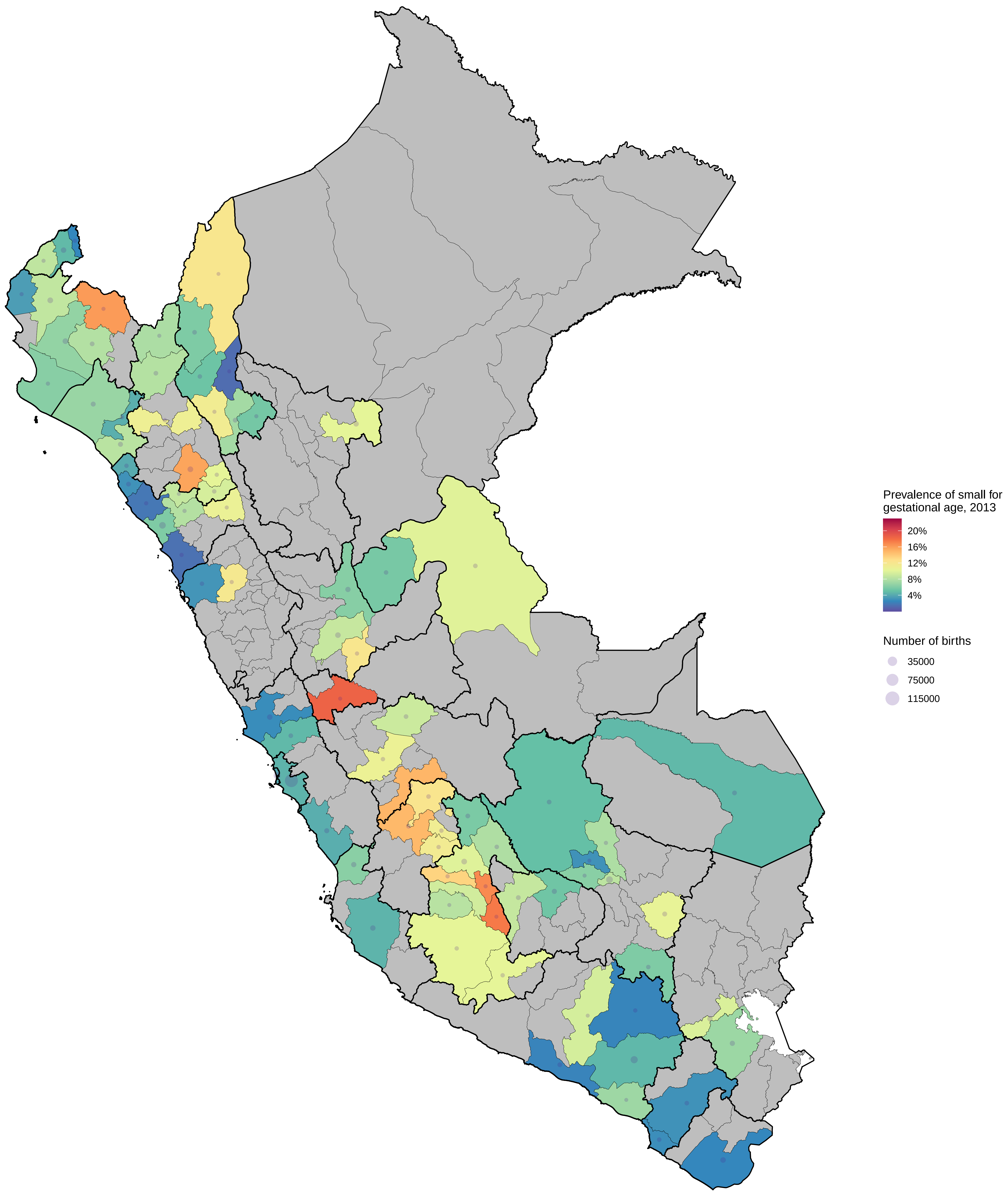

Prevalence of small for gestational age, 2013
20%
16%
12%
8%
4%
Number of births
35000
75000
115000

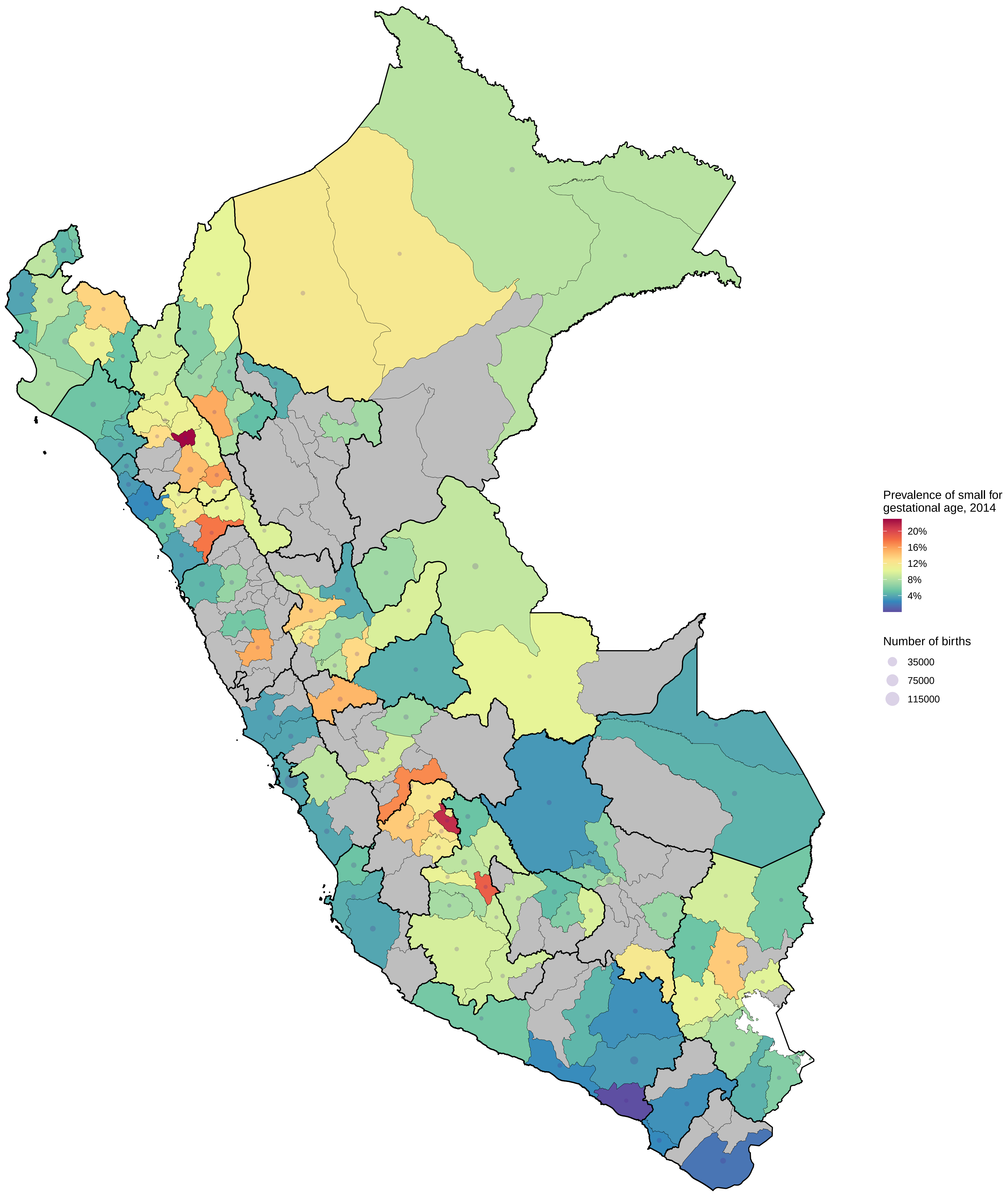

Prevalence of small for gestational age, 2014
20%
16%
12%
8%
4%
Number of births
35000
75000
115000

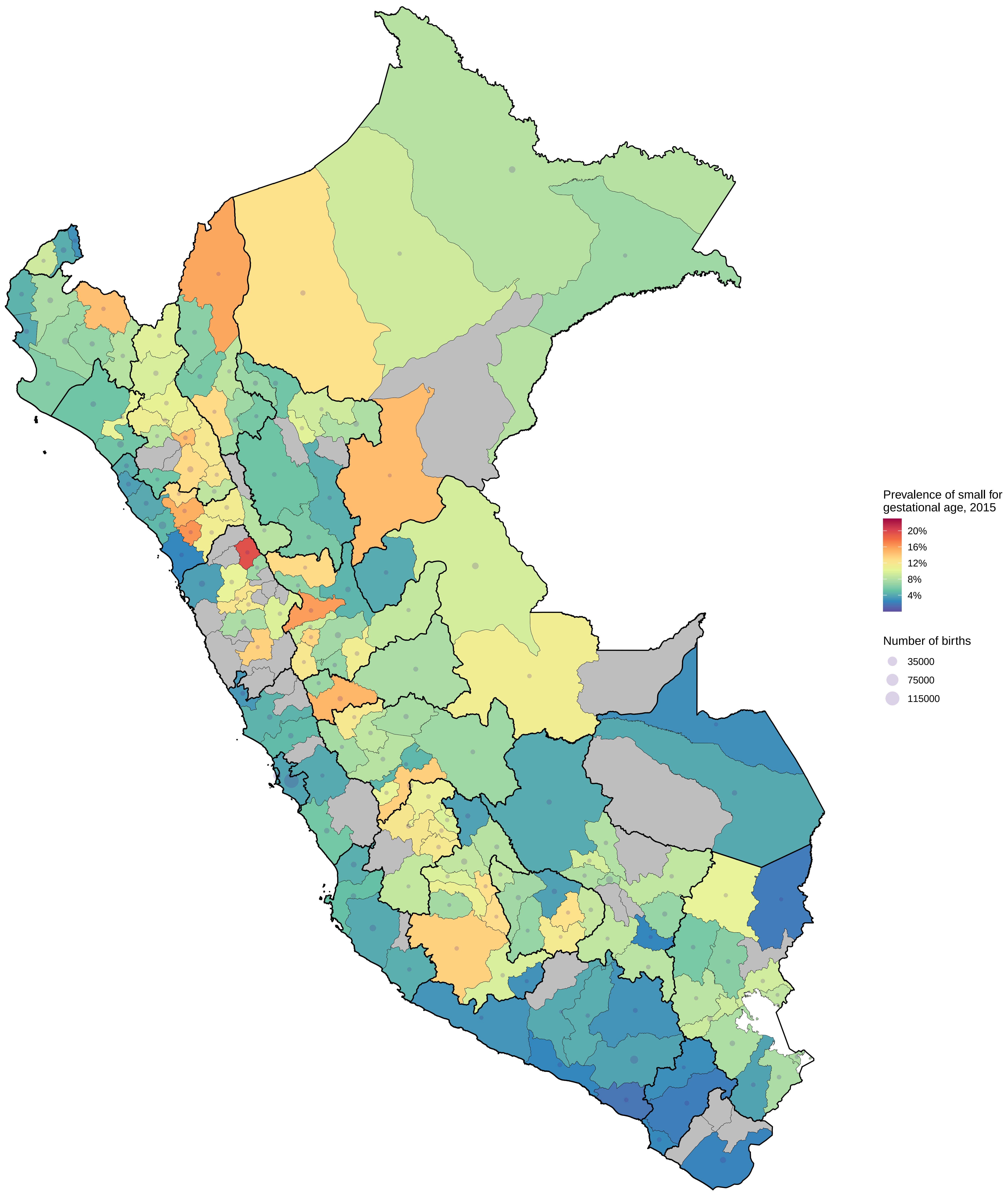
Prevalence of small for gestational age, 2015
20%
16%
12%
8%
4%
Number of births
35000
75000
115000

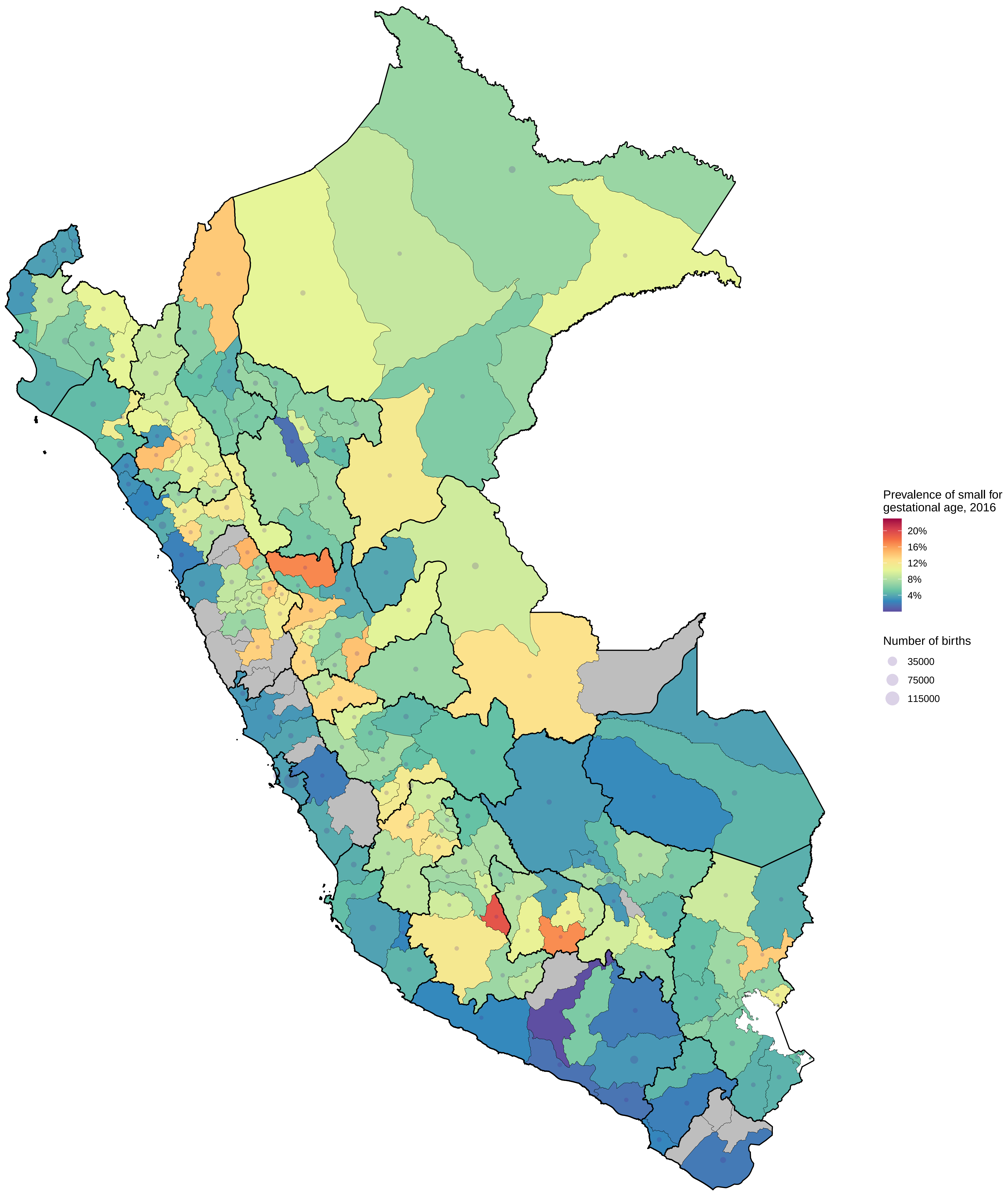

Prevalence of small for gestational age, 2016
20%
16%
12%
8%
4%
Number of births
35000
75000
115000

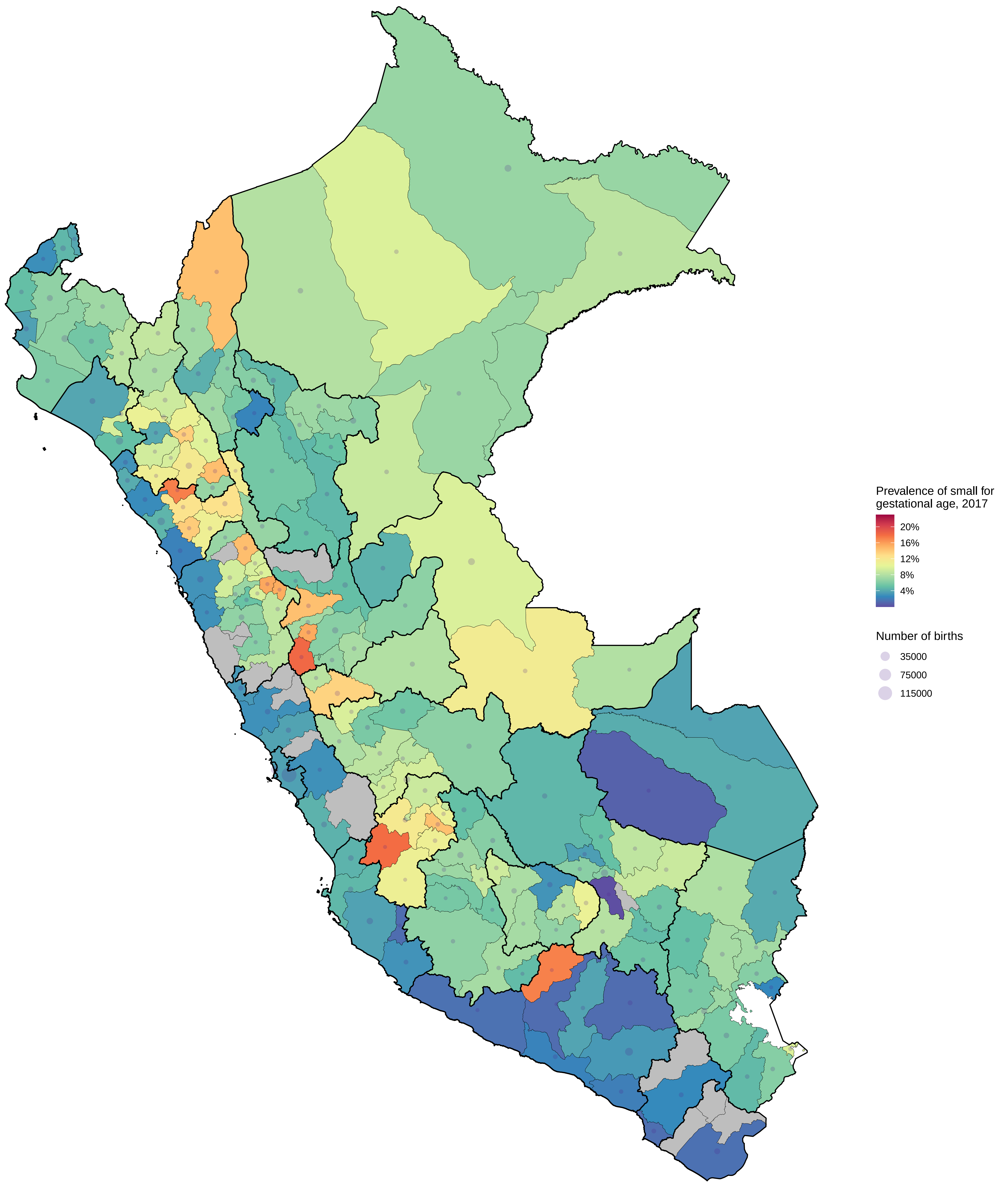

Prevalence of small for gestational age, 2017
20%
16%
12%
8%
4%
Number of births
35000
75000
115000

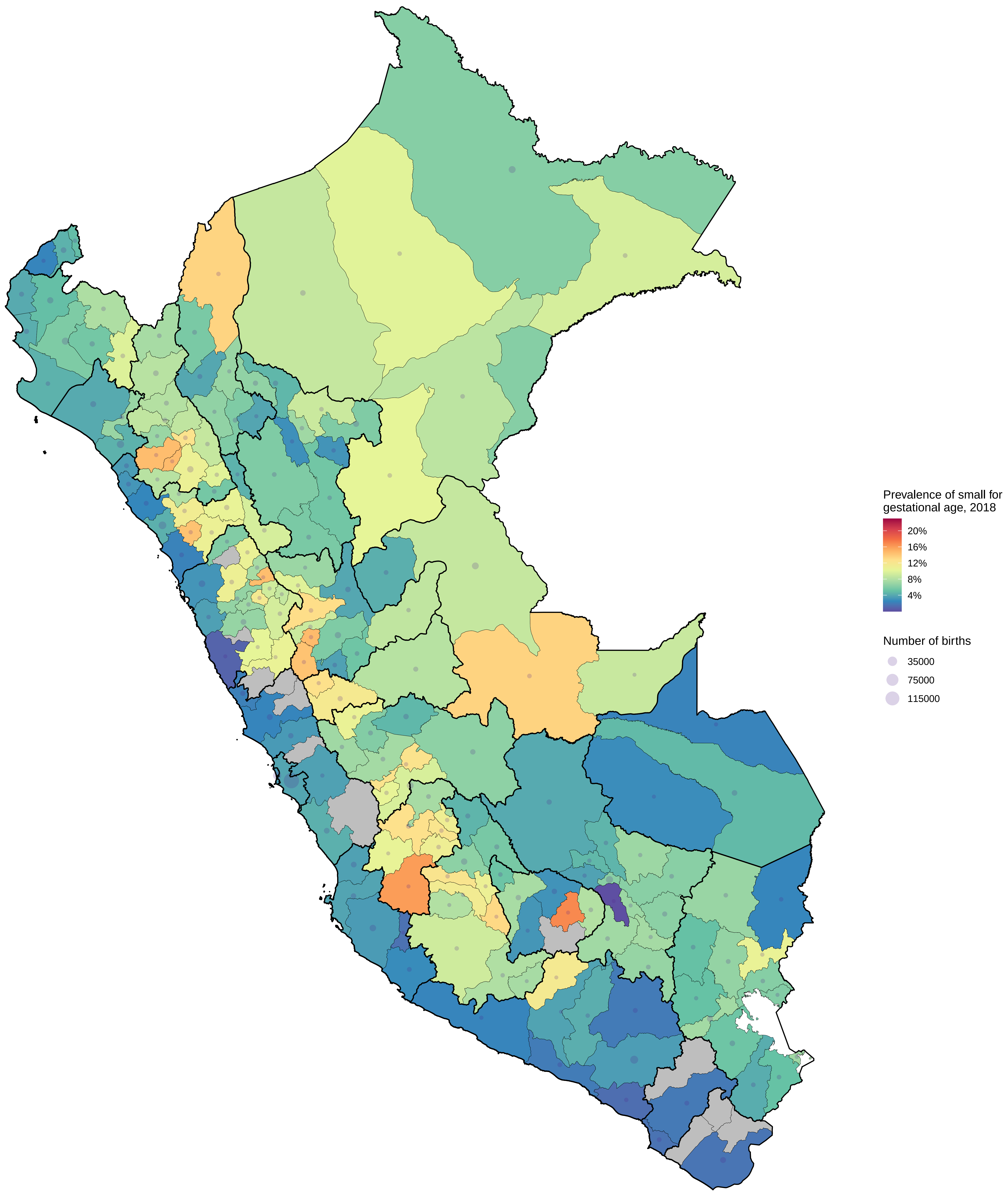
Prevalence of small for gestational age, 2018
20%
16%
12%
8%
4%
Number of births
35000
75000
115000

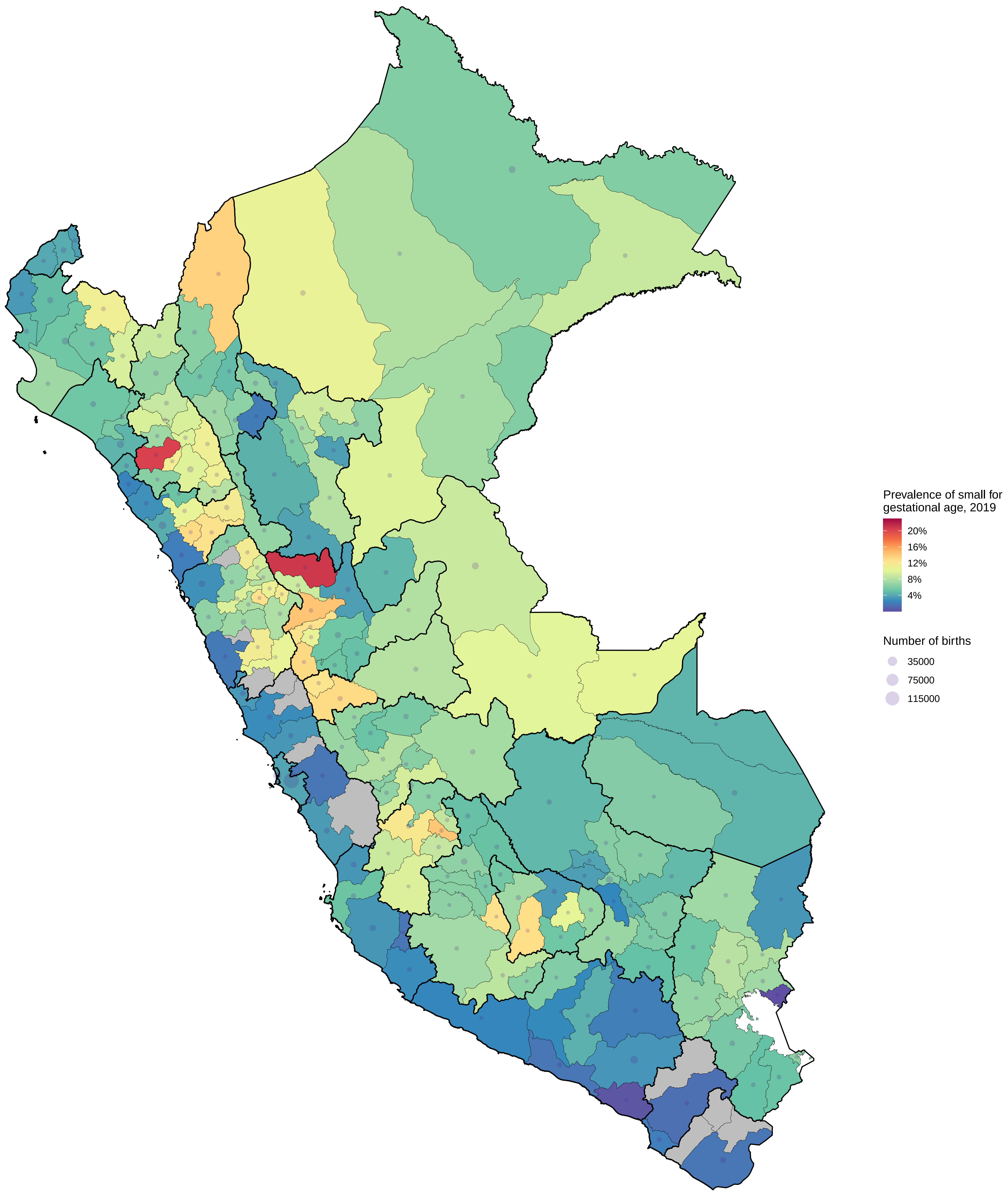
Prevalence of small for gestational age, 2019
20%
16%
12%
8%
4%
Number of births
35000
75000
115000
